# Combinatorial group testing for efficient scaling across biological applications

**DOI:** 10.1038/s41467-026-77055-5

**Published:** 2026-08-25

**Authors:** Lorenzo Talamanca, Julian Trouillon

**Affiliations:** Institute of Molecular Systems Biology, ETH Zürich, Zürich, Switzerland

**Keywords:** High-throughput screening, Software, Computational platforms and environments, Assay systems

## Abstract

Combinatorial group testing can reduce experimental costs and turnaround time by strategically pooling samples to minimize the number of measurements needed for a given experiment. Despite broad potential utility, it remains underutilized due to its intrinsic complexity and the lack of implementation tools. Here we present PoolPy, a unified end-to-end framework and web platform to benchmark, automate, and decode combinatorial group testing strategies. PoolPy tailors pooling designs to application-specific constraints, such as time, cost, or signal dilution, across experiment types. By implementing ten different pooling algorithms, which we comprehensively benchmark in silico across >100,000 conditions, we identify key design trade-offs that define pooling applicability to specific use cases. We experimentally validate PoolPy across diverse applications, including protein-ligand interaction screening, RT-qPCR viral testing and genome-wide protein-DNA interaction profiling, achieving a 60 to 93% reduction in number of measurements needed. Overall, PoolPy provides a scalable, user-friendly ecosystem to increase throughput and reduce costs across biological applications. PoolPy is available at https://poolpy.trouillonlab.org for open use.

## Abstract (web summary)

Combinatorial group testing can reduce experimental costs but remains challenging to apply due to its complexity and the lack of implementation tools. Here, the authors present PoolPy, a complete combinatorial group testing framework, enabling up to 93% cost reduction across biological assays.

## Introduction

Biomedical screening and molecular profiling experiments typically rely on the individual measurement of samples, which becomes prohibitively expensive and labor-intensive as experimental scales increase. Combinatorial group testing consists in pooling samples in pre-defined groups before testing to increase the information obtained per test, therefore significantly reducing screening costs and turnaround time^1^. These pools are specifically designed to allow the exact decoding of the pooled results, enabling the direct identification of individual samples that have the tested property. This approach is used broadly across fields, including for human infection testing to screen large populations at reduced costs^2–5^, or for software engineering^6,7^. Group testing is also increasingly used in large-scale molecular profiling assays to expand possible search spaces, such as for drug discovery^8^, CRISPR-based phenotypic screening^9–11^, or transcriptomics^12^, metabolomics^13^, proteomics^14^, and high-dimensional imaging^15^ measurements.

Despite its benefits, combinatorial group testing remains underutilized due to its complex design and decoding processes, and the lack of accessible implementation tools. In addition, multiple strategies have been proposed with varying performances and ranges of application^16^, making it challenging to select appropriate designs for specific use cases. Due to the lack of implementation tools, studies that use group testing frequently re-implement individual basic methods without comparative evaluation, requiring laborious additional steps and often resulting in suboptimal experimental designs.

Here, we present PoolPy, a web platform to perform all steps of a combinatorial group testing experiment for any application (https://poolpy.trouillonlab.org). For each use case, PoolPy generates up to 13 distinct pooling designs and provides a comprehensive set of performance metrics to enable design selection based on logistical and assay-specific constraints such as cost, turnaround time, or signal dilution limits. Using PoolPy to benchmark over 100,000 testing conditions in silico, we identify the most suited designs across different performance indicators reflecting real-world constraints. We further demonstrate the use of PoolPy across assay types using protein-ligand interaction screening, viral infection testing, and genome-wide protein-DNA interaction profiling.

## Results

### PoolPy addresses the current gap in combinatorial pooling implementation tools

**Fig. 1:**
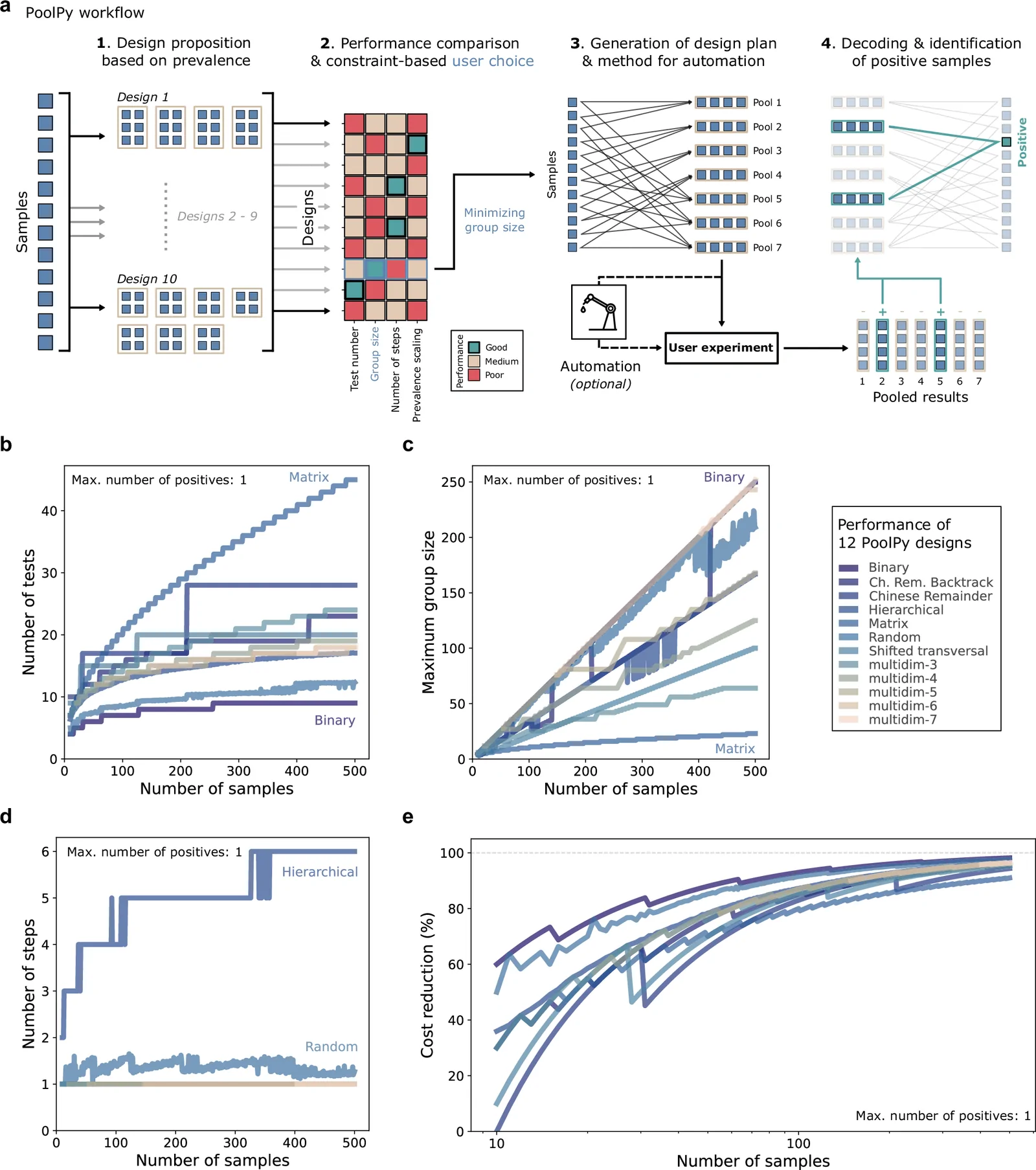
Overview of the PoolPy workflow and design performance at low prevalence. **a** The PoolPy workflow, following four major steps (left to right). **b**–**e** Comparison of number of tests (**b**), maximum group size (**c**), number of steps (**d**), and cost reduction derived from test number reduction (**e**), for PoolPy designs over 10 to 500 samples for cases with at most one positive sample. Max., maximum. Source data are provided as a Source Data file.

To enable the use of combinatorial group testing across applications, we developed PoolPy, a unified framework and web platform to compare, download, automate, and decode combinatorial group testing designs for any application (Fig. 1a). Unlike existing tools that focus on standard single-readout assays^17,18^, PoolPy provides a complete end-to-end ecosystem, including the generation of method files for robot-assisted automation and the decoding of multi-readout experiments for large-scale assays. While existing tools typically support 1-2 pooling methods of interest^17,18^, PoolPy implements 10 conceptually different strategies (Supplementary Notes 1, 2), including all the most used methods and a design introduced here that is based on the theoretical limits of information theory (Supplementary Fig. 1), enabling comprehensive benchmarking of design performances. PoolPy generates pooling designs that require less tests, less rounds of testing, and does so faster than existing tools (Supplementary Fig. 2). In addition, PoolPy implements both a standard binary decoder and a continuous decoder for compressed sensing, enabling combinatorial testing for genome-scale molecular profiling assays with complex quantitative readouts^9,10,12^. For each use case, PoolPy provides 8-13 distinct designs to enable rational selection of optimal designs based on real-world logistical constraints, including prevalence, cost, time, and signal dilution (Supplementary Note 3).

### PoolPy enables high cost reduction at low prevalence

To delineate applicability and guide user choice, we benchmarked over 100, 000 screening conditions in silico based on key performance indicators (Supplementary Table 1; full dataset available at https://doi.org/10.5281/zenodo.18660061^19^). At low prevalence, PoolPy generates designs that are highly efficient in reducing test numbers, with as little as nine tests needed to identify up to one positive sample among 500 in a single step (Fig. 1b–d), or 0.018 tests per sample. To achieve this performance, the corresponding design exploits large groups that contain up to half of all samples (Fig. 1c and Supplementary Fig. 1), which can become limiting due to signal dilution (Supplementary Note 3). In contrast, designs using smaller groups require increased test numbers (Supplementary Fig. 3), reflecting an apparent tradeoff that drives design applicability. By reducing the number of measurements needed for a given experiment, PoolPy designs enable cost reductions of 60 to 98% when compared to individual testing across different numbers of samples at low prevalence (Fig. 1e), enabling the accurate testing of large numbers of samples using a fraction of the required measurements and costs.

### Prevalence defines combinatorial pooling performance

**Fig. 2:**
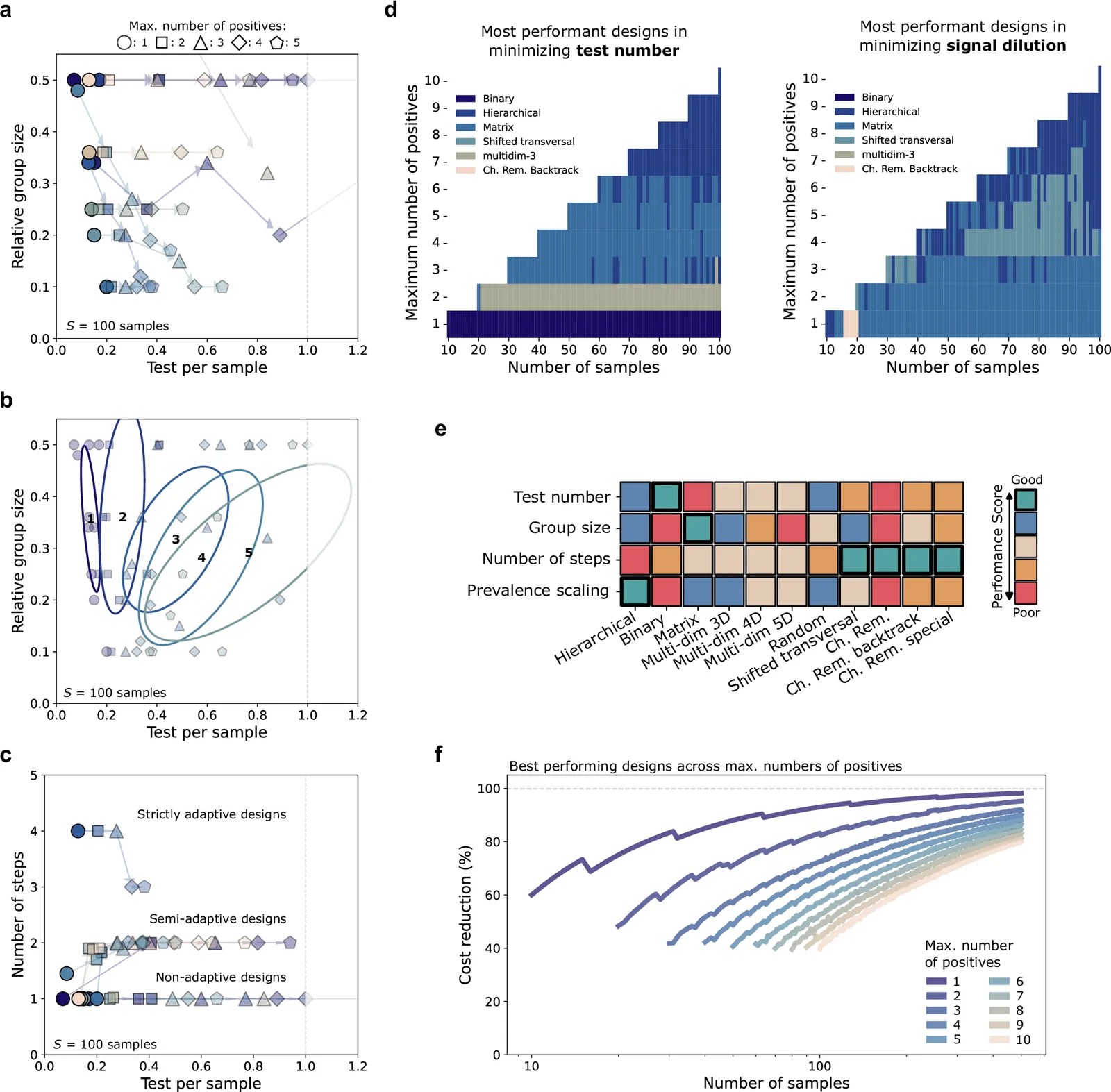
PoolPy generates optimal designs across prevalence values. **a**–**c** Relative group size (**a**,** b**) and number of steps (**c**) required by each design related to the number of tests per sample over 1–5 maximum number of positive samples (reflected by marker shape). Markers are either colored by designs (**a**,** c**) or by maximum number of positives (**b**). The number of positives also indicated with confidence ellipses over one standard deviation (**b**). **d** Heatmaps showing the best performing PoolPy designs for all combinations of 10–100 samples with at most 10% positive samples in terms of minimizing test number (left) or signal dilution (right). **e** Performance summary heatmap of PoolPy designs across four key performance indicators. **f** Cost reduction, derived from the reduction in test numbers, enabled by the best performing PoolPy design at each number of samples (10–500) and maximum number of positives (1–10) for any prevalence up to 10%. Max., maximum. Source data are provided as a Source Data file.

PoolPy shows that designs relying on small group sizes scale better with increased numbers of positive samples (Fig. 2a, b and Supplementary Fig. 3), although they then often required an additional step for validations (Fig. 2c and Supplementary Fig. 4), or 2–6 steps for adaptive designs (Supplementary Fig. 5). In general, group testing performs best at low prevalence, enabling the use of more efficient designs with lower maximum positive numbers (Fig. 2b). However, lowering this threshold increases the chance of misparametrization (drawing more positives than the defined maximum) (Supplementary Fig. 6), which can potentially increase the number of required tests, highlighting the importance of a priori prevalence estimation. PoolPy provides guidance and a tool for determining the appropriate pooling parameters to balance this tradeoff based on estimated prevalence (Supplementary Note 4).

Overall, we comprehensively assessed designs performance and showed that no single design emerges as universally optimal (Fig. 2d, e and Supplementary Fig. 7), highlighting the need for case-specific design choice based on logistical constraints and screening parameters. Specifically, six different PoolPy designs performed best in reducing either test number or signal dilution in at least one scenario across 10–100 samples with up to 10% of positives (Fig. 2d). While the largest cost reductions are achieved at low prevalence, which is consistent with the known requirement for signal sparsity in information theory^1^, PoolPy designs still enable considerable cost reductions of 40–98% across these conditions when compared to individual testing (Fig. 2f). Given the broad landscape of group testing applications, PoolPy addresses the current lack of flexible comparative frameworks to tailor combinatorial designs to specific experimental requirements.

### Diverse PoolPy designs accommodate varying signal dilution tolerance

**Fig. 3:**
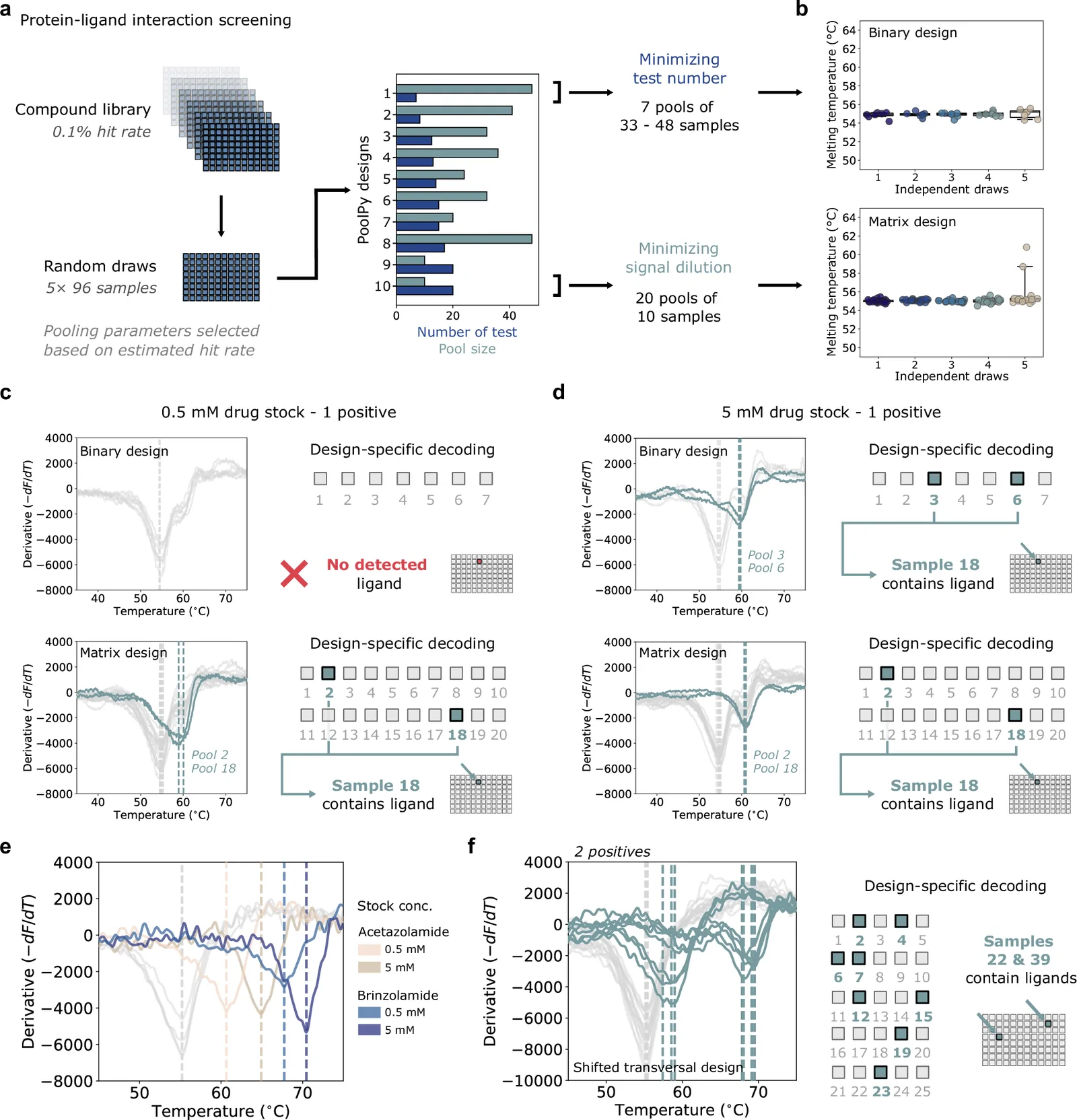
PoolPy tailors combinatorial pooling based on assay-specific signal dilution tolerance. **a** Schematic representation of protein-ligand interaction screening and the corresponding PoolPy designs. **b** Pooled ligand screening for human carbonic anhydrase II across five random draws of 96 samples containing up to one positive sample. Data are represented as box plots indicating the median (center line), 25^th^ and 75^th^ percentiles (box bounds), and 5^th^ and 95^th^ percentiles (whiskers) with all individual points shown. **c**,** d** Thermal shift assay of all pools for the draw that contained a positive (acetazolamide) sample (left) and the corresponding decoding scheme (right) for the binary design minimizing test number (top) or the matrix design minimizing signal dilution (bottom), for acetazolamide stock concentrations of 0.5 mM (**c**) or 5 mM (**d**). Positive and negative samples are shown in cyan and gray, respectively. **e** Ligand interaction for human carbonic anhydrase II across two concentrations of acetazolamide or brinzolamide. **f** Pooled ligand screening (shown as in **c**,** d**) using the shifted transversal design for human carbonic anhydrase II with two positive samples containing 0.5 mM acetazomalide and 5 mM brinzolamide, respectively. Positive and negative samples are shown in cyan and gray, respectively. Source data are provided as a Source Data file.

To evaluate PoolPy in an experimental setting, we first performed a protein-ligand interaction screening with a known interaction using thermal shift assays. We used the human carbonic anhydrase II enzyme (hCAII) and its known drug inhibitors as a model system due to their well characterized dynamics across a broad range of interaction strengths^20^. By testing two different PoolPy designs, we investigated the tradeoff between experimental throughput and signal dilution on sets of 96 samples containing up to one positive sample (Fig. 3a). Using liquid-handling automation to pool samples from PoolPy-generated automation method files, we screened ligand interactions for hCAII (Fig. 3b). To test signal dilution tolerance, we first used low ligand stock concentrations (0.5 mM). The positive samples, containing the known hCAII ligand acetazolamide, were correctly identified among 96 samples by performing 20 pooled tests using the design that minimizes signal dilution, while they were missed by the high-efficiency design using only 7 tests due to higher signal dilution from its larger pools (Fig. 3c). When increasing ligand stock concentrations to 5 mM, both designs correctly identified the positive sample (Fig. 3d). Based on the signal dilution tolerance of their specific assay, which can be obtained by measuring a serial dilution of pool sizes (Supplementary Fig. 8), users can thus select an appropriate combinatorial design. These results demonstrate that the ability of PoolPy to tailor designs to application-specific constraints is essential for robust screening.

Then, we aimed to further assess the performance of PoolPy designs in a scenario with multiple positive samples, which additionally have varying signal intensities. To that aim, we first selected two conditions, among varying concentrations of the known hCAII inhibitors acetazolamide and brinzolamide (Fig. 3e), with a large signal intensity difference (10 ^∘^C difference in melting temperature shift) that were then randomly added to a set of 96 samples to be tested. After pooling and testing the samples following the design which minimizes test number within a single testing round in these conditions, PoolPy correctly identified the two positive samples using only 25 pooled measurements (Fig. 3f), equivalent to a reduction of about 74% in used resources and cost compared to individual testing. While pools containing samples with brinzolamide showed higher signal strength than those containing only acetazolamide, as expected (Fig. 3e), the adequate pool sizes still enabled the correct identification of all positive pools, and thus all positive samples after decoding. Overall, the broad diversity of designs generated by PoolPy enables optimal group testing across different testing conditions, including varying numbers of positive samples and signal intensities.

### PoolPy enables scaling and cost reduction across prevalences

**Fig. 4:**
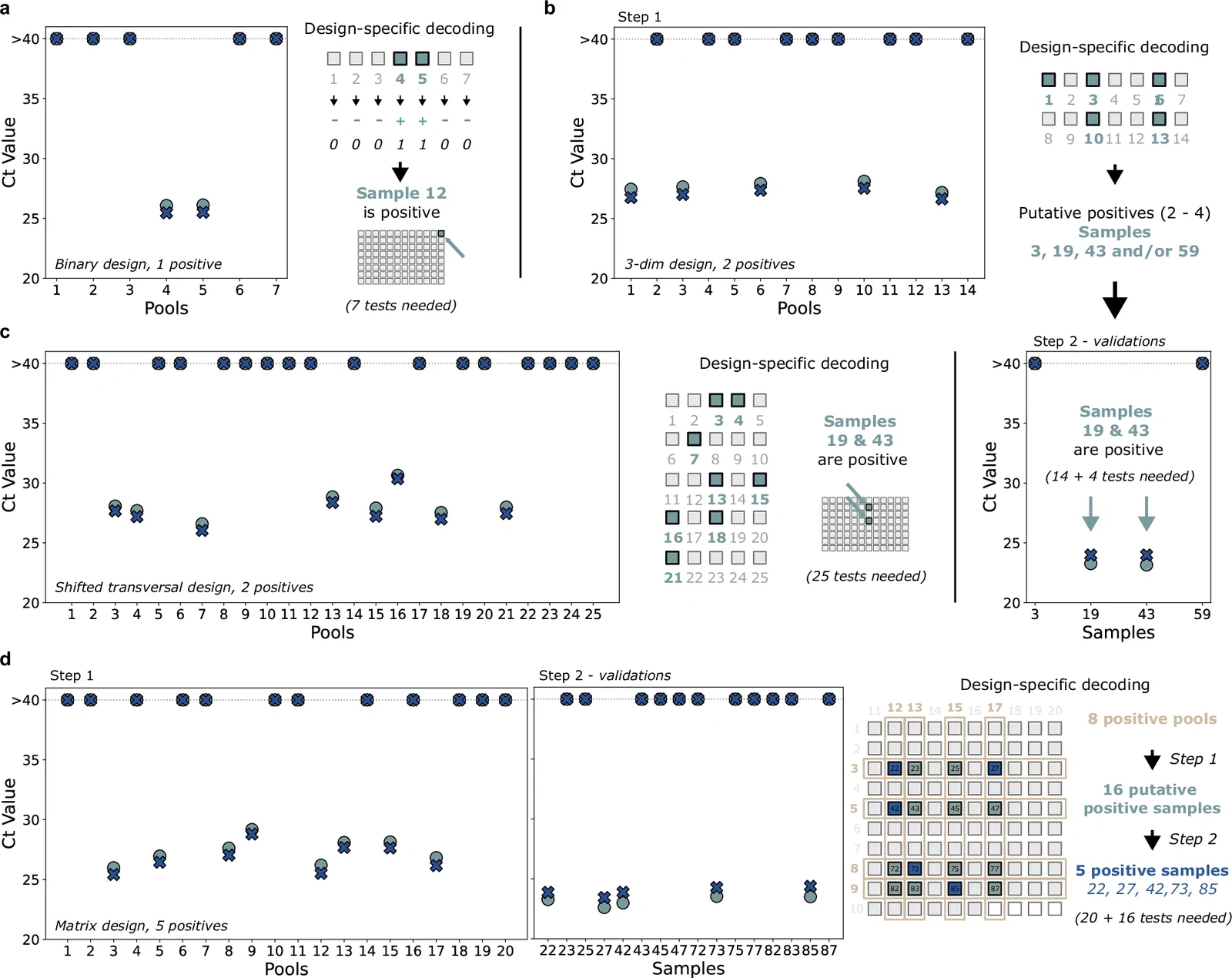
PoolPy enables substantial cost reduction across prevalence values in RT-qPCR testing. **a**–**d** SARS-CoV-2 RT-qPCR testing results for pooled assays of 96 samples containing one (**a**), two (**b**,** c**), or five (**d**) positive samples. In each case, an appropriate pooling design was chosen using PoolPy, leading to the use of the binary (**a**), 3-dim (**b**), shifted transversal (**c**), and matrix (**d**) designs. Results are shown for all pooled testing steps (**b**,** d**). Markers denote amplification of two different regions of the SARS-CoV-2 nucleocapsid RNA (N1: circle, N2: cross). Positive tests show Ct values below 40 for both regions. Tests with undetermined Ct values (above 40) are negative. Source data are provided as a Source Data file.

To illustrate the broad range of applications compatible with PoolPy, we applied combinatorial group testing to viral RT-qPCR testing. Here, we tested three different conditions, each with 96 samples, including either one, two or five samples that were randomly spiked with SARS-CoV-2 RNA. In each scenario, we used PoolPy to select the most suited pooling design that minimizes test numbers. In the case of a single positive sample, we could identify that sample by performing a single step of SARS-CoV-2 RT-qPCR testing on seven pools only and decoding the results with PoolPy (Fig. 4a), representing a cost reduction of about 93% compared to individual testing. For the set containing two positive samples, we tested the two designs that minimize test numbers either with any number of steps (Fig. 4b), or strictly within a single step (Fig. 4c). The single-step design identified the two positive samples using 25 tests, or a cost reduction of about 74%. While two steps were needed in the other case, the two positive samples were identified using only 18 tests, providing a cost reduction of 81%. Lastly, we tested a high prevalence scenario with five positive samples, reflecting a complex condition where the performance of group testing is known to diminish^1^ and where mostly adaptive designs still provide substantial cost reductions (Supplementary Fig. 7). Here, the two-step PoolPy design correctly identified all five positive samples using 36 tests (Fig. 4d), or a 62.5% cost reduction. Overall, PoolPy enables considerable scaling and cost reduction across varying test conditions and applications.

### PoolPy enables combinatorial group testing for high-throughput multi-readout assays

**Fig. 5:**
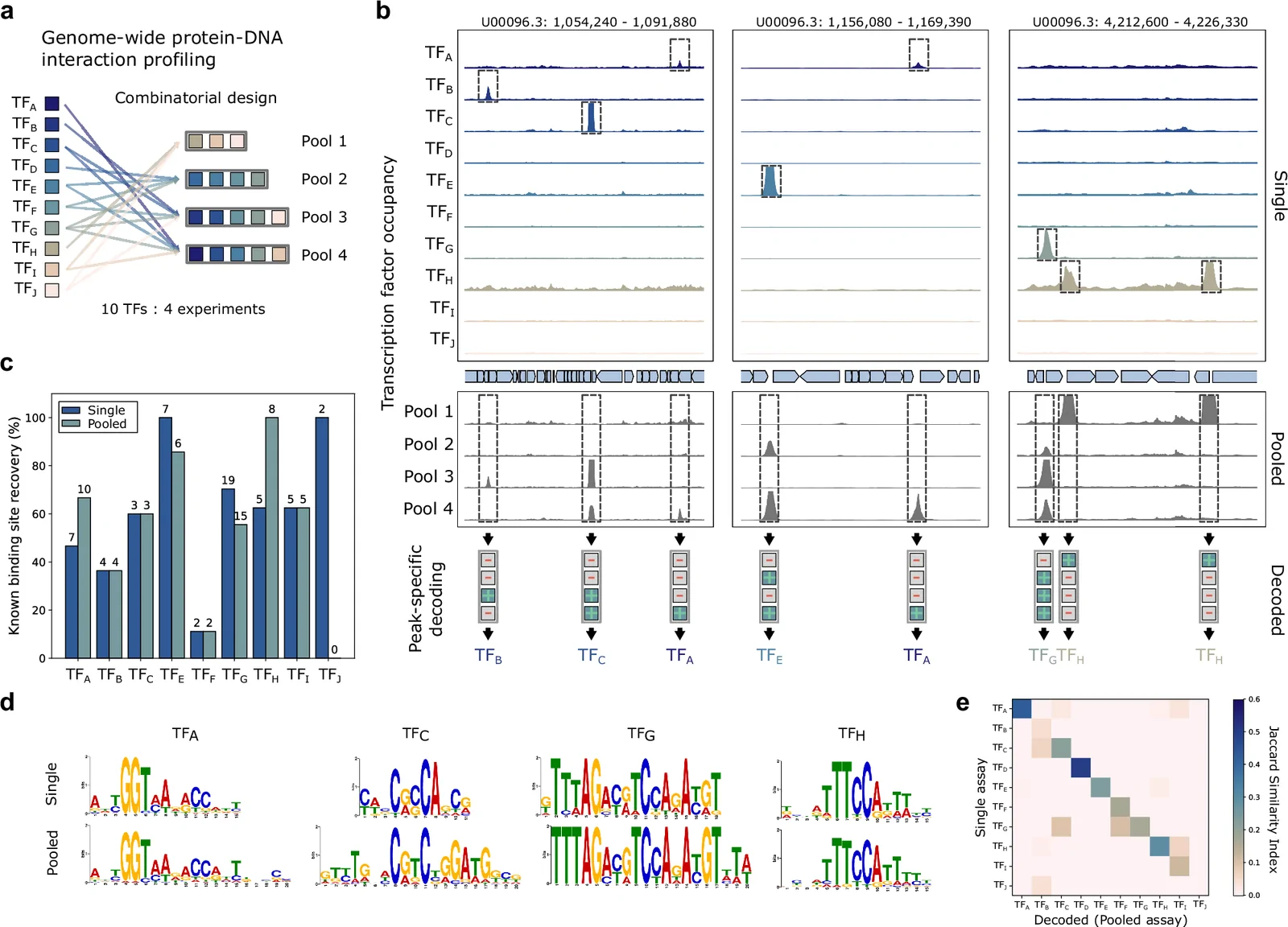
PoolPy enables combinatorial pooling for genome-wide profiling assays. **a** DAP-seq pooling design used in (**b**–**e**) to profile ten *E. coli* TFs in four assays. **b** Transcription factor occupancy (fold enrichment over negative control) tracks over three example regions for single (top) or pooled (bottom) assays. For each region, all 14 plots are scaled to the same y-axis value. +, positive; -, negative. **c** Recovery of known binding sites between standard and decoded pooled assays for the nine TFs with previously known binding sites. The total numbers of recovered binding sites are added on top of bars representing the percent recovery. **d** Identified DNA motifs across all peak regions from single or decoded pooled assays for the four TFs with significant motifs identified. **e** Similarity score matrices of all detected peaks between single and decoded pooled assays. Source data are provided as a Source Data file.

Next, we aimed to demonstrate PoolPy use on a multi-readout application by using genome-wide protein-DNA interaction profiling of ten transcription factors (TF) in the model bacterium *Escherichia coli*. Using DNA affinity purification sequencing (DAP-seq)^21^ to probe genome-wide DNA interactions of the ten TFs, we compared the use of a PoolPy design relying on four pooled tests to the individual profiling of TFs (Fig. 5a, b and Supplementary Data 1). Here, we used PoolPy in its multi-readout mode, where each genome-wide binding event is considered as an individual readout and is thus uniquely decoded. PoolPy assigns each binding event to the corresponding TF according to the binary decoding of the presence or absence of binding at that location in the pooled measurements (Fig. 5b and Supplementary Data 1). The ten decoded results from the four pooled assays showed high concordance with the results obtained with standard individual assays, correctly recovering most known binding sites (Fig. 5c), consensus DNA-binding motifs (Fig. 5d), and expected similarity levels (Fig. 5e). Overall, PoolPy correctly identified genome-wide binding sites for ten TFs using four measurements, representing a 2.5-fold increase in throughput, or a 60% cost reduction. Such an increase represents a considerable cost reduction for large experiments, whether using DAP-seq on numerous TFs, or for any other compatible profiling assays. If signal dilution is not limiting, cost reductions greater than 60% could be achieved in wider screens (Fig. 1b and Supplementary Fig. 7), enabling the exploration of vast search spaces that were previously cost-prohibitive across a diverse range of molecular readouts. These results establish PoolPy as a robust framework for scaling up screening and large-scale functional genomics while maintaining the high resolution of standard individual assays.

## Discussion

While combinatorial group testing holds potential for substantial scaling and cost reduction across numerous biological applications, it remains underutilized due to its complexity and the lack of implementation tools. Here we present PoolPy, a user-friendly tool and web app to perform all steps of a group testing experiment without requiring prior mathematical expertise. By integrating ten conceptually different pooling algorithms, PoolPy allows their comprehensive benchmarking to tailor experimental designs to user-defined constraints, including time, cost, and signal dilution. PoolPy is compatible with a wide array of applications, as illustrated by our experimental validations using thermal shift assay, RT-qPCR and DAP-seq across varying prevalence values. PoolPy enables considerable scaling and cost reduction, with 60–93% fewer measurements needed across these different assays. Overall, PoolPy is a broadly applicable, user-friendly tool that can scale up biological experiments across fields, enabling the exploration of larger search spaces at reduced costs.

By relying on sample pooling, group testing can lead to signal dilution. This depends on the specific application and can be mitigated physically, for example, by proportionally increasing the stock concentrations of tested compounds to preserve the target final concentrations in the assay. When concentrations are constrained by solubility or sample availability, characterizing the signal dilution tolerance of the assay is beneficial to choose an appropriate pooling design. To guide design selection, PoolPy provides a comprehensive comparison of maximum pool sizes across designs, enabling algorithm selection according to signal dilution, as illustrated by our experimental validations.

The choice of pooling design can also be influenced by other technical constraints, such as limited sample amounts or expected presence of false positives, such as with promiscuous compounds^22^. High rates of expected false positives should be accounted for when deciding on a maximum number of positives, as it considers both true and false positives, potentially leading to the use of less efficient designs. Here again, PoolPy provides corresponding metrics to compare design performances in terms of individual sample usage or pooling accuracy. In addition, because pooling reduces the total number of tests and relies on multi-pool validation, the final number of false positives arising from stochastic experimental noise is typically lower than in individual testing^1^.

In summary, PoolPy bridges the gap between group testing theory and laboratory execution, offering a unified framework to compare, apply, automate, and decode optimal combinatorial designs tailored to any compatible applications, including multi-readout molecular profiling assays. PoolPy is open-source, includes an accessible web interface and user instructions, and represents a flexible unified framework to integrate potential novel designs in the future.

## Methods

### Implementation of group testing designs

#### General notation

First, we define ${\mathbb{S}}=\left\{s\right\}$ as the set of *S* samples with *s* = 0, . . . , *S* − 1. Of these *S* samples, we assume that up to *D* can be positive. For the purposes of this description, we consider all positive results to be true positives and all negative results to be true negatives. The same can be formalized for the *W* pools, and their set ${\mathbb{W}}=\left\{w\right\}$ with *w* = 0, . . . , *W* − 1. In general, we represent a set as a math-bold letter, the cardinality as a capital letter, and an element as a lower-case letter. We use the notation ${{\mathbb{S}}}_{w}$ to indicate the set of samples that are grouped together in pool *w*, as well as ${{\mathbb{W}}}_{s}$ to indicate the set of pools where sample *s* is present. *S* is defined at the beginning of the problem as the number of samples to test, which should be known; *W* is dependent on the pooling strategy used and varies accordingly. For this reason, *W* can be written as a function of up to three variables: the method, the number of samples, and the maximum number of positives, *W* = *W*(method, *S*, *D*). However, we refer to the number of pools as *W* to improve readability. We also use a pool-assigning matrix *K*, which is a boolean matrix of size *S* × *W* where *K*_*sw*_ = 1 means that sample *s* is in pool *w*. According to our previous description and definitions, row *s* of *K* has a simple bijection to ${{\mathbb{W}}}_{s}$ and, conversely, column *w* has one to ${{\mathbb{S}}}_{w}$. Defining the *K* matrix, $\left\{{{\mathbb{S}}}_{w}\right\}$, or $\left\{{{\mathbb{W}}}_{s}\right\}$ is equivalent and fully characterizes the pooling strategy.

#### Matrix design

In the matrix design, all samples are ideally arranged in a square matrix, and all samples in the same row or column are combined into a single pool, yielding $W \sim 2\sqrt{S}$. While *S* cannot always be arranged in a perfect square, this has minimal impact on the effectiveness of this design. We aim to analytically determine the number of pools needed as well as to express the sets ${{\mathbb{S}}}_{w}$ and, conversely, ${{\mathbb{W}}}_{s}$ in a compact form. Let the number of rows be $A=\lceil \sqrt{S}\rceil$ and the number of columns *B* = ⌈*S*/*A*⌉. It follows that *A* − 1 ≤ *B *≤ *A*. To identify the row-derived pools, assuming the matrix is filled left-to-right and top-to-bottom, we have

1 $${{\mathbb{S}}}_{a}=\left\{s| \lfloor s/B\rfloor=a\right\}.$$

Here, the floor operation is equivalent to the computational ’integer division’. For the column pools, we define

2 $${{\mathbb{S}}}_{b}=\left\{s| s\,{{\mathrm{mod}}}\,\,B=b\right\}.$$

Finally, we can define the total number of pools and their assignment as

3 $$W=A+B\quad {\mathbb{W}}={\mathbb{A}}\cup {\mathbb{B}}\quad w\in {\mathbb{W}}=\left\{\begin{array}{l}w\in {\mathbb{A}}\quad \,{\mbox{if}}\,\quad w\le A\\ w-A\in {\mathbb{B}}\quad \,{\mbox{if}}\,\quad w\ge A\end{array}\right.$$

#### N-dimensional design

As previously proposed^5,23^, we expand the matrix formalism to any *N* dimensional matrix design, assuming that *S *≥ 2^*N*^. We can start by calculating the size of the *N*-dimensional matrix. Let $L=\lceil \root N \of {S}\rceil$ so that (*L*−1)^*N*^ < *S *≤ *L*^*N*^. Therefore, all sides are of length *L* or *L* − 1, which can be determined iteratively. Let *L*_*n*_ denote the size of dimension *n*. There is an arbitrary ordering of all the *L*_*n*_ that we break by defining *η* such that

4 $${L}_{n}=\left\{\begin{array}{ll}L\hfill\quad &n\le \eta \\ L-1\quad &n > \eta \end{array}\right.$$

By definition, if *η* = *N* − 1 then all *L*_*n*_ = *L*. We now aim to transform a one-dimensional number into a set of binary numbers to achieve a pool-assigning function. We note that each sample belongs to exactly one pool along each dimension. We can then proceed as for the matrix design. When ${L}_{n}=L\forall n\in {\mathbb{N}}| 0\le n\le N-1$, this is equivalent to a base change. If all dimensions are of the same size (*L*), passing from a single number identifying the sample to an *N-*dimensional coordinate is equivalent to a base-*L* expansion. The sample number *s* is written in base *L*, padding with zeros to *N* digits, implicitly defining ${{\mathbb{W}}}_{s}$. Formally, we define the map

5 $${{{\rm{BL}}}}\,(s)=({s}_{0},{s}_{1},...,{s}_{N})\quad | \quad {\sum}_{n}{L}^{n}\cdot {s}_{n}=s$$

Each coordinate in this *N*-dimensional space represents which pool of that dimension is the correct one for sample *s*. By construction, we know that each sample is in *N* distinct pools. The set of pools for sample *s*, with *s*_*i*_ defined implicitly from Eq. (5) is

6 $${{\mathbb{W}}}_{s}=\left\{w| w=nL+{s}_{n}\,{{{\rm{for}}}}\,n=0,...,N-1\right\}.$$

We can conversely define ${{\mathbb{S}}}_{w}$ as

7 $${{\mathbb{S}}}_{w}=\left\{s| {s}_{k}=w-Lk\right\}.$$

All these definitions require the base transformation of Eq. (5), which can be performed in parallel, though sequential transformations remain fast for typical sample sizes  < 10^4^. The above formulas assume that ${L}_{n}=L\forall n\in {\mathbb{N}}| 0\le n\le N-1$. When this assumption does not hold, the necessary base-like transformation can still be similarly defined as

8 $${{\mbox{BN}}}_{S}(s)=({s}_{0},{s}_{1},...,{s}_{N})\quad | \quad {\sum}_{n}\left({\prod }_{m=0}^{n}{L}_{m}\right)\cdot {s}_{n}=s$$

where the BN operator generalizes the ’base change’ operation to dimension *N* with *S* samples. We remind that having defined *N* and *S* uniquely defines all the *L*_*n*_ given Eq. (4). We can rewrite Eq. (6) to fit with the general case:

9 $${{\mathbb{W}}}_{s}=\left\{w| w=\left({\sum }_{m=0}^{n}{L}_{m}\right)+{s}_{n}\,{{{\rm{for}}}}\,n=0,...,N-1\right\}.$$

as well as

10 $${{\mathbb{S}}}_{w}=\left\{s| {s}_{k}=w-{\sum }_{m=0}^{k}{L}_{m}\right\}.$$

As (*S*, *N*) uniquely define $\left\{{{\mathbb{W}}}_{s}\right\}$ and $\left\{{{\mathbb{S}}}_{w}\right\}$, the *N* dimensional pooling strategy is fully characterized.

#### Binary design

The binary design can be viewed as the maximum dimensional design for every *S*, since it relies on rewriting *s* in base 2. In addition, the binary design only measures one out of the two pools of every dimension. This strategy is highly advantageous when *D* = 1, but its performance falls off quickly as soon as *D *≥ 2. We can write as before:

11 $${{{\rm{B2}}}}\,(s)=({s}_{0},{s}_{1},...,{s}_{N-1})\quad | \quad {\sum}_{{{\rm{n}}}}{2}^{n}\cdot {s}_{n}=s$$

The pools are defined as ${{\mathbb{S}}}_{w}=\left\{s| {s}_{w}=1\right\}$, giving by construction *W* = ⌈log_2_(*S*)⌉. This design minimizes the number of tests needed when there is exactly one positive sample. However, to consider the case *D* = 1, all samples need to be tested at least once. This can be done by leaving the 0th sample empty, guaranteeing to check for *D* = 1 and not only *d* = 1. This adds at most one pool, but in practice often does not, since in many cases ⌈log_2_(*S*)⌉ = ⌈log_2_(*S* + 1)⌉, especially for large *S*.

#### Random design

The random design consists in setting the number of pools *W* and the number of samples per pool *S*_*W*_, then extracting for each pool independently *S*_*W*_ samples from ${\mathbb{S}}$^24^. Once this assignment is done, the design is complete. In practice, however, using this strategy effectively requires exploring multiple configurations rather than relying on a single draw. In general, the choice of *N* and *S*_*W*_ is not trivial. Therefore, our implementation strategy tests all combinations of *N* and *S*_*W*_ such that

12 $$\begin{array}{rcl}mc&\le &{S}_{W}\le MC\\ mp&\le &W\le MP\\ mc&=&\sqrt{S}\\ MC&=&S/2\\ mp&=&{{\mbox{log}}}_{2}(S)\\ MP&=&2\sqrt{S}.\end{array}$$

Each combination of *N* and *S*_*W*_ is tested 5 times, but to find the true optimal condition, many more tests could be needed. However, we found that fluctuations between different random draws were generally much smaller than the differences observed across configurations, particularly for large *S*.

#### Shifted transversal design (STD)

For this non-adaptive design previously described^25–27^, we consider the transpose of the pool-assigning matrix, setting *M* = *K*^⊺^ to follow the notation of the original publication. We first define a closed rotation of indices, *σ*:

13 $$\sigma :{{\mathbb{R}}}^{S}\to {{\mathbb{R}}}^{S}\quad \sigma (\bar{x})=\sigma \left(({x}_{0},{x}_{1},...,{x}_{S-1})\right)=({x}_{S-1},{x}_{0},{x}_{1},...,{x}_{S-2}).$$

We can apply multiple (*m*) times the *σ* operator, denoted as *σ*^*m*^. For any prime number *q* such that *q* < *S*, we define the compressing power of *q* with respect to *S*, noted *Γ*(*q*, *S*) or *Γ*, as the smallest integer *γ* for which *q*^*γ*+1^ > *S*. The idea behind the shifted transversal design is to construct a simple initial sample-pool assignment and then the full *K* matrix by systematically shifting it with the *σ* operator. As previously described^25^, this design aims to satisfy two conditions: (i) limit the number of pools in which any pair of samples co-occur, and (ii) keep the intersection sizes between pools roughly constant, in order to maximize the information content of the design. Each layer of the shifted transversal design is composed of *q* pools, and in each layer, every sample is placed in exactly one pool. Given the definition of *M*, that is the transpose of *K*, column *i* represents the set of pools containing sample *i*. Let *C*_*ij*_ denote the *i*th column in the *j*th layer. To break symmetry, in the first (0th) layer, the first (0th) sample is assigned to the first (0th) pool, so that *C*_00_ = (1, 0, . . . , 0). The general construction is then given by

14 $${C}_{sj}={\sigma }^{{t}_{sj}}{C}_{00}\quad {t}_{sj}={\sum }_{c=0}^{\Gamma }{j}^{c}\left\lfloor \frac{s}{{q}^{c}}\right\rfloor .$$

This procedure generates, for each *j*, a submatrix with *S* columns and *q* rows. This method is able to generate *k *≤ *q* + 1 layers. Stacking the first *k* layers vertically yields the complete pooling matrix *M* of shape (*q* ⋅ *k*) × *S*. The construction above does not by itself specify the choice of *q* or the required number of layers. However, necessary conditions can be derived for the method to produce a non-adaptive (1-step) pooling design. For a given maximum number of positives *D*, one must find a prime *q* such that

15 $$D\cdot \Gamma (q,S)\le q.$$

Then, the shifted transversal design is obtained by merging the first *k* = *DΓ*(*q*, *S*) + 1 ≤ *q* + 1 layers, resulting in a design with *q* ⋅ *k* pools and guaranteeing a 1-step pooling experiment.

#### Chinese remainder design

This method, based on the Chinese Remainder theorem^7^, is conceptually similar to the shifted transversal design and also yields a 1-step pooling strategy. Let $\left\{{p}_{1}^{{e}_{1}},{p}_{2}^{{e}_{2}},...,{p}_{J}^{{e}_{J}}\right\}$ be a set of prime numbers *p* each associated with its exponent *e* such that

16 $${\prod}_{j}{p}_{j}^{{e}_{j}}\ge {S}^{D}.$$

For each *j*, we construct a pooling submatrix *M*_*j*_, and stack them vertically into the full pooling matrix *M*. As above, we take *M* = *K*^⊺^, so rows correspond to pools and columns to samples. In this strategy, *M*_*j*_ has size *t*_*j*_ × *S* with ${t}_{j}={p}_{j}^{{e}_{j}}$ and therefore *M* is a (*W* = *T*) × *S* matrix where

17 $$T={\sum}_{j}{t}_{j}={\sum}_{j}{p}_{j}^{{e}_{j}}$$

For each *M*_*j*_ we proceed row by row and identify the ones (i.e., which samples are part of that particular pool). In particular, in matrix *M*_*j*_ we have samples of row 0 ≤ *l* < *t*_*j*_ to be one if and only if column *k* is equal to *l* modulo *t*_*j*_:

18 $${\left({M}_{j}\right)}_{kl}=\left\{\begin{array}{l}1\quad l=k\,{\mbox{mod}}\,{t}_{j}\hfill\quad \\ 0\quad \,{\mbox{all other cases}}\,\quad \end{array}\right.$$

This formula uniquely defines the pooling strategy and provides a constructive strategy to build it once $\left\{{p}_{1}^{{e}_{1}},{p}_{2}^{{e}_{2}},...,{p}_{J}^{{e}_{J}}\right\}$ is know. In particular, it was shown that this construction yields a 1-step (non-adaptive) pooling design^7^. The simplest variant sets *e*_*j*_ = 1 ∀ *j* and is generally suboptimal in terms of minimizing *T* = *W*, but allows for much faster strategy generation and can be sufficient in practice^7^.

#### Chinese remainder backtrack

A different version of the Chinese Remainder method with lower *W* can be derived by allowing *e*_*j*_ ≠ 1 and minimizing *T* = *W*, which requires choosing the primes and exponents such that $\left\{{p}_{1}^{{e}_{1}},{p}_{2}^{{e}_{2}},...,{p}_{J}^{{e}_{J}}\right\}$ minimizes $T={\sum }_{j}{p}_{j}^{{e}_{j}}$. The optimal set can be determined using a backtracking approach described here. First, the maximal set of subsequent primes is determined as

19 $${{\min }_{J}}{\prod }_{j}^{J}{p}_{j}\ge {S}^{D}.$$

Once J is fixed, we search for the combinations of exponents minimizing *T* = *W* respecting Eq. (16) with the constraint that ${p}_{i}^{{e}_{i}}\le {p}_{J}\forall i$. This faster strategy is implemented in PoolPy as the Backtrack variant of the Chinese Remainder design.

#### Chinese remainder special case *D* = 2

The general formalism of the Chinese Remainder construction can be further developed for the special cases of *D* = 2 and *D* = 3 ^7^, which are based on exploiting different bases. For *D* = 2, a more efficient pooling strategy (both computationally and in terms of the number of pools) can be obtained by using base-3 representations. With *q*: = ⌈log_3_(*S*)⌉, a 1-step pooling strategy with *W* = (*q*^2^ + 5*q*)/2 can be developed. Following the base change notation adopted in the Binary design section, we have

20 $${{{\rm{B3}}}}\,(s)=({s}_{0},...,{s}_{q-1})\quad | \quad {\sum}_{{{\rm{n}}}}{3}^{n}\cdot {s}_{n}=s.$$

Analogously to the binary design, the first 3*q* columns of the pooling matrix *M* are defined by

21 $${{\mathbb{S}}}_{w}=\left\{s| {s}_{\lfloor w/3\rfloor }=w\,{\mbox{mod}}\,3\right\}.$$

These columns already suffice to identify the position of a single positive sample. However, in the case of two positives, this construction may be insufficient. To fully resolve the *d* = 2 case, an additional $$\left(\begin{array}{c}q\\ 2\end{array}\right)$$ columns are added. Let $({q}^{{\prime} },{q}^{{\prime\prime} })$ with $0\le {q}^{{\prime} } < {q}^{{\prime\prime} } < q$ denote all $$\left(\begin{array}{c}q\\ 2\end{array}\right)$$ possible pairs of natural numbers smaller than *q*. Following the notation of ref. ^7^, the additional columns of *M* are then defined as

22 $${{\mathbb{S}}}_{{q}^{{\prime} },{q}^{{\prime\prime} }}=\left\{s| {s}_{{q}^{{\prime} }}={s}_{{q}^{{\prime\prime} }}\right\}.$$

These extra columns can distinguish between two positives by giving a relation of the various digits of the two positives in base-3, allowing their unique identification.

#### Chinese remainder special case *D* = 3

The case *D* = 3 can also be treated explicitly^7^. Here, a base-2 representation is used. We define *q* as *q*: = ⌈log_2_(*S*)⌉ to describe a pooling strategy with *W* = 2*q*^2^ − 2*q*. Following the notation above we have

23 $${{{\rm{B2}}}}\,(s)=({s}_{0},...,{s}_{q-1})\quad | \quad {\sum}_{n}{2}^{n}\cdot {s}_{n}=s.$$

We again define $({q}^{{\prime} },{q}^{{\prime\prime} })$ with $0\le {q}^{{\prime} } < {q}^{{\prime\prime} } < q$ as the $$\left(\begin{array}{c}q\\ 2\end{array}\right)$$ possible pairs of natural numbers smaller than *q*. We also call $(v{\prime},{v}^{{\prime\prime} })$ the four distinct vectors such that $({v}^{{\prime} },{v}^{{\prime\prime} })\in {\left\{0,1\right\}}^{2}$. Again, we define the *K* matrix as

24 $${{\mathbb{S}}}_{{q}^{{\prime} },{q}^{{\prime\prime} },v{\prime},{v}^{{\prime\prime} }}=\left\{s| {s}_{{q}^{{\prime} }}={v}^{{\prime} },{s}_{{q}^{{\prime\prime} }}={v}^{{\prime\prime} }\right\}.$$

#### Hierarchical design

The hierarchical design is different from all the other designs implemented in PoolPy, as it is the only strictly adaptive method. This design aims to minimize the number of total experiments by iteratively isolating positive samples step by step. The core principle relies on partitioning the complete set of samples ${\mathbb{S}}$ into non-overlapping subsets ${{\mathbb{S}}}_{w}$ with the following properties:

25 $$\begin{array}{lll}{{\mathbb{S}}}_{w}\cap {{\mathbb{S}}}_{v}&=&\left\{\begin{array}{ll}{{\mathbb{S}}}_{w}\quad &\,{\mbox{if}}\,\quad w=v\\ \varnothing \quad &\,{\mbox{if}}\,\quad w\ne v\end{array}\right.\quad={{\mathbb{S}}}_{w}{\delta }_{w,v}\\ &&\bigcup\limits_{w\in {\mathbb{W}}}{{\mathbb{S}}}_{w}={\mathbb{S}}\\ &&{{\max }_{v,w}}\left(\left\vert {S}_{v}-{S}_{w}\right\vert \right)\le 1\end{array}$$

where *δ* is the Kronecker delta applied to sets. After this step, all pools are tested, and the procedure is recursively applied to each positive pool. The case of *D* = 1 is of theoretical interest to determine the optimal pool size. A possible intuition is that a binary strategy, i.e., splitting into two sets at each step, is optimal, which would require

26 $$W=2\cdot {{\mbox{log}}}_{2}(S)$$

However, assuming that all splits are constant in *k* subsets at each step, the generalized number of required tests becomes

27 $${W}_{k}=k\cdot {{\mbox{log}}}_{k}(S)=k\cdot \frac{\ln (S)}{\ln (k)}$$

To minimize the number of tests *W*_*k*_ with respect to *k*, we must minimize the objective function $\frac{k}{\ln (k)}=\ln {\left({k}^{k}\right)}^{-1}$ since the factor ln(S) does not affect minimization. Because this objective function is strictly positive for all *k* > 1, minimizing it is equivalent to maximizing its reciprocal $\ln \left({k}^{k}\right)$. We can then safely exponentiate the reciprocal without affecting the value of *k* for which the maximum occurs, yielding the final maximization objective:

28 $$o(k)={k}^{\frac{1}{k}}$$

The maximum is found when *k* = *e*. As only integer splits are feasible experimentally, this continuous optimization aligns conceptually with the well-known partition problem for maximum product. In brief, the partition problem for *N* aims to find the set of numbers such that the sum is equal to *N* and the product is maximum. Assuming that all numbers in the set are equal to *n*, their product *P* is

29 $$P={n}^{N/n}={\left({n}^{1/n}\right)}^{N}$$

Since *N* > 1, maximizing *P* is equivalent to maximizing *n*^1/*n*^ = *o*(*n*). The established integer solution to this partition problem dictates that all *n* are equal to 3 apart from one (in the case where $N\,{{\mathrm{mod}}}\,\,3=2$) or two (in the case where $N\,{{\mathrm{mod}}}\,\,3=1$), which are equal to 2. In the context of pooling, this corresponds to always dividing samples into three pools until we have 4 or 2 samples left, when they are divided in two pools. Here, we defined this strategy for the cases where each pool is tested. In theory, if the number of positives is known, up to one pool per step can be excluded from testing for further optimization. For higher *D*, the optimal splitting architecture diverges from this base case, and PoolPy instead determines the best pooling steps by exhaustive search.

### Parametrization based on estimated prevalence

In practice, the precise number of positives in a sample set is typically unknown, but the prevalence in the population can be estimated. We therefore discuss how to estimate optimal pooling strategies when the true number of positives $\bar{d}$ is drawn from a binomial probability distribution based on an estimated prevalence value. It should be noted that when working with prevalence there is always a possibility that $\bar{d}$ is higher than the used threshold $(i.e.,D < \bar{d})$. Assuming test results are correct and considering *S* samples extracted from a population with prevalence *ρ*, for any given *D* we have

30 $$\begin{array}{rcl}&&{{{{\mathcal{P}}}}}_{S}\left(\bar{d} > D\right)=1-{\sum }_{d=0}^{D}{\rho }^{d}{(1-\rho )}^{S-d}\left(\begin{array}{c}S\\ d\end{array}\right)\simeq \\ &&1-\frac{1}{\sqrt{2\pi }\sqrt{S\rho (1-\rho )}}\int_{-\infty }^{D}dx{e}^{-\frac{{(x-D)}^{2}}{2S\rho (1-\rho )}}=\\ &&1-\frac{\sqrt{S}}{\sqrt{2\pi }\sqrt{\rho (1-\rho )}}\int_{-\infty }^{D/S}dx{\prime} {e}^{-\frac{S{(x{\prime} -D/S)}^{2}}{2\rho (1-\rho )}}.\end{array}$$

Eq. (30) yields the exact or approximate probability of misparametrization (drawing more positives than the set threshold *D*) when applying a pooling strategy to *S* samples taken from a population with prevalence *ρ*. Importantly, increasing *D* is not the only way to reduce this probability for fixed *S* and *ρ*. An alternative is to split the experiment, for instance, by performing two separate pooling experiments with *S*/2 samples each instead of pooling all *S* samples together. In this case, however, a correction for the Family Wise Error Rate (FWER) is required. In general:

31 $${{{{\mathcal{P}}}}}_{S}\left(\bar{d} > D\right)=1-\left({\prod}_{{S}_{i}}\left(1-{{{{\mathcal{P}}}}}_{{S}_{i}}\left(\bar{d} > {D}_{i}\right)\right)\right)$$

with

32 $${\sum}_{i}{S}_{i}=S\quad \,{\mbox{and}}\,\quad {\sum}_{i}{D}_{i}=D.$$

In the case of an equal split into *η* parts, we find that Eq. (31) becomes

33 $${{{{\mathcal{P}}}}}_{S}\left(\bar{d} > D\right)=1-{\left(1-{{{{\mathcal{P}}}}}_{S/\eta }\left(\bar{d}\ > \ D/\eta \right)\right)}^{\eta }.$$

Taking a first-order approximation recovers the usual FWER correction:

34 $${{{{\mathcal{P}}}}}_{S}\left(\bar{d} > D\right)\simeq 1-\left(1-\eta {{{{\mathcal{P}}}}}_{S/\eta }\left(\bar{d} > D/\eta \right)\right)=\eta {{{{\mathcal{P}}}}}_{S/\eta }\left(\bar{d} > D/\eta \right).$$

Finally, we emphasize that the value of $\bar{d}$ is determined by the two system parameters *S* and *ρ*, which are either fixed or explicitly specified in the formulas above. The concept of acceptable error in this case reflects the probability that one pool contains a number of positives *d* > *D*. This will not have the same consequences for all designs, as some are more robust to error detection. While some designs, including the hierarchical and matrix designs, will typically still identify the positive samples when *d* > *D*, non-adaptive designs will often require additional tests and might even lead to inconclusive results in some cases. Therefore, it is important to tune the acceptable error as a function of the chosen pooling design in conjunction with other constraints.

### Decoder

The PoolPy decoder identifies positive samples (for binary readouts) or quantifies each sample’s signal contribution (for continuous readout) for a given pooling design and experimental readout.

#### Binary decoder

Let us assume the readout ${\mathbb{O}}$ to be a binary vector of length *W*. We define ${\mathbb{P}}$ as the set of independent combinations that would produce a specific readout given a pool assigning matrix and the maximum number of positive samples, ${\mathbb{P}}=f({\mathbb{O}},K,D)$. If the experiment has only one possible solution, the cardinality of ${\mathbb{P}}$ is *P* = 1. The first step in the decoding procedure is to take only the subset of possible positive samples. Interpreting ${{\mathbb{W}}}_{s}$ also as a binary vector we can define the following quantities:

35 $${O}_{s}={\mathbb{O}}\cdot {{\mathbb{W}}}_{s}\quad \,{\mbox{and}}\,\quad {W}_{s}=| | {{\mathbb{W}}}_{s}| |={{\mathbb{W}}}_{s}\cdot {{\mathbb{W}}}_{s}$$

for each sample *s*. We can then define

36 $${O}_{s}\le {W}_{s}\quad \forall s.$$

If *O*_*s*_ < *W*_*s*_ then sample *s* cannot be positive, as there must be at least one well where *s* is present that is not part of the readout. If less than 10^2^ combinations of up to *D* samples are possible, all combinations are tested, and the decoder returns the ones explaining the experimental readout, ${\mathbb{O}}$. In the majority of cases, especially if the pooling experiment was designed appropriately based on fair prior knowledge, this limit is not reached. If the number of possible combinations exceeds 10^2^ or the number of unique samples present across all combinations, the set of putative possible samples ${{\mathbb{S}}}_{{\mathbb{O}}}=\left\{s| {\mathbb{O}}\cdot {{\mathbb{W}}}_{s}={O}_{s}={W}_{s}\right\}$ is returned. If we assume that the maximum number of positives *D* is not known after the tests are done, the decoding procedure will generally return ${{\mathbb{S}}}_{{\mathbb{O}}}$. Overall, our decoding procedure is optimized for typical experimental conditions - 10^1^ − 10^4^ samples and up to 1 − 10 positives - and might not be optimal in other scenarios.

#### Continuous decoder

To allow continuous readout pooling experiments, we introduced a dedicated LASSO-based (least absolute shrinkage and selection operator) decoding procedure. For every unique readout ID with continuous readout ${\mathbb{O}}$, we perform a linear regression with L1 penalty (LASSO), as previously outlined^10^, estimating the best sample signal vector $\vec{\gamma }$ such that

37 $$\vec{\gamma }={{\min }_{\vec{g}}}\left(| | \vec{g}\cdot K-{\mathbb{O}}| {| }^{2}+\alpha | | \vec{g}| {| }_{1}\right)$$

PoolPy’s toolkit can also account for sample dilution effects in the decoding procedure by adapting a pool-specific correction. We also generalize this formulation for different experimental data types with an elastic net fit:

38 $$\vec{\gamma }={{\min }_{\vec{g}}}\left(| | \vec{g}\cdot K-{\mathbb{O}}| {| }^{2}+\alpha \left(\beta | | \vec{g}| {| }_{1}+(1-\beta )/2| | \vec{g}| {| }_{2}\right)\right)$$

with user-defined parameters *α* and *β*. We note that the case *β* = 1 of the elastic net is equivalent to the LASSO, and the case *β* = 0 is equivalent to the Ridge regression. Poolpy also allows to perform a parameter sensitivity analysis through a grid search of the elastic net parameters, returning all grid boxes and directly optimizing an error function; four error functions are implemented: mean square error, mean absolute error, root mean square error, and R-squared.

### In silico design benchmarking

To compare design performances across testing conditions, we used PoolPy to generate design matrices following the mathematical implementations described above. Overall, we generated matrices for 8–13 designs (all applicable designs in each scenario) for $N\in \left\{10,11,\ldots,1000\right\}$ and $D\in \left\{1,2,\ldots,\min (\lfloor \frac{N}{10}\rfloor,10)\right\}$ generating 107,795 unique pooling matrices. These results are available via Zenodo^19^ and can also be re-generated using the following PoolPy command line:


python pool_N.py –start 10 –stop 1001 –step 1 –directory <target_folder>


To validate the design matrices for each design and combination of *N* and *D* considered, we sampled one thousand possible combinations of up to *D* positives among *N* samples. The probability distribution of the actual number of positive samples *d *≤ *D* follows the probability distribution:

39 $$P(d)=\frac{\left(\begin{array}{c}N\\ d\end{array}\right)}{{\sum }_{d\le D}\left(\begin{array}{c}N\\ d\end{array}\right)}.$$

Such a distribution has been selected as it mimics the distribution of *d* when enumerating all possible instances of *d *≤ *D*. For each combination, that is equivalent to one experiment, we calculated the theoretical readout and then decoded the signal using the PoolPy binary decoder to evaluate design performance. We report in the web app and via our data deposition^19^ key summary statistics obtained from this large benchmarking of pooling designs across conditions. All summary statistics can be generated by running the following PoolPy command line:


python Calculate_metrics.py –start 10 –stop 1001 –step 1 –directory <target_folder>


### Pooling sensitivity and specificity

Since experimental assays might have sensitivity (se) and specificity (sp) below 1, we define as pooling sensitivity (pse) and specificity (psp) the probability of not having any false positive and false negatives, respectively. When decoding results without a defined maximum number of positives and obtaining a set of putative positive samples, ${{\mathbb{S}}}_{{\mathbb{O}}}$, we can calculate the pooling sensitivity and specificity as

40 $$\,{\mbox{pse}}=1-{(1-{\mbox{se}})}^{| | {\mathbb{O}}| | }\quad \,{\mbox{and}}\,\quad \,{\mbox{psp}}=1-{(1-{\mbox{sp}})}^{(W-| | {\mathbb{O}}| | )}.$$

Since *N* samples are tested, these values should not be compared directly to se and sp. Therefore, the baseline for comparisons to individual testing is the naive sensitivity (nse) and specificity (nsp):

41 $$\,{\mbox{nse}}=1-{(1-{\mbox{se}})}^{d}\quad \,{\mbox{and}}\,\quad \,{\mbox{nsp}}=1-{(1-{\mbox{sp}})}^{S-d}.$$

### Sampling from an infinite population

Another way to generate group testing designs is to consider only the known or estimated prevalence, *ρ*, assuming an infinite population^18^. In this case, pool sizes that perform best on the population with prevalence *ρ* are determined independently of the number of samples to test, rather than constraining the number of samples and the maximum number of positives. To evaluate designs from this framework, we use the number of tests per sample required by a given design, *τ*, as the main metric, assuming test results to be correct.

#### Prevalence-based two-step hierarchical design

First, we implemented a prevalence-based hierarchical design that relies on a classical two-step procedure^2,18^. This two-step design relies on the pooling of samples from the population in pools of size *M*, then re-testing all *M* samples from each positive pool. In this case, we can explicitly write

42 $$\tau (\rho,M)=\frac{1}{M}\left(1+M\left(1-{(1-\rho )}^{M}\right)\right).$$

This function can be directly optimized to obtain the best pool size at any given prevalence.

#### Prevalence-based multi-step hierarchical design

Here, we implement a hierarchical design where the number of steps is not pre-determined, but rather searched to minimize the number of tests. In this setting, each step has *m*_*l*_ pools, with the index *l* from 1 to *L* where *L* is the number of steps. The pools to be tested in the first step thus have ${M}_{0}={\prod }_{l=1}^{L}{m}_{l}$ samples. In general, a pool from step *l* contains ${M}_{l}={\prod }_{i=l+1}^{L}{m}_{i}$ samples. We define ${M}_{l}^{*}=\mathop{\prod }_{i=1}^{l}{m}_{i}$ (including for generality the 0th step with *m*_0_ = 1) such that ${M}_{l}{M}_{l}^{*}={M}_{0}$, implying ${M}_{0}^{*}=1$. We also introduce the quantity $\left\{{\pi }_{l}^{i}\right\}$ as the readout from pool *i* of step *l*. To simplify notation, we define the probability that one pool at step *l* is positive as

43 $${\psi }_{l}(\rho,{M}_{l})=1-{(1-\rho )}^{{M}_{l}}=:{\psi }_{l}.$$

We also introduce the general readout as

44 $${\pi }_{l}=\mathop{\sum}_{i}{\pi }_{l}^{i}$$

that is the number of positive pools at step *l*. At each step, at least one pool is positive, allowing to calculate the estimated number of positives on the conditional probability. The number of pools to test is the expected number of positive pools ${\langle {\pi }_{i}\rangle }_{{\pi }_{i}\ > \ 0}$ times the number of sub-pools in each of them *m*_*i*+1_, giving 〈*π*_*i*_〉 as

45 $$\langle {\pi }_{i}\rangle={M}_{i}^{*}{\psi }_{i}$$

However, by conditioning the probability on at least one pool being positive, we have to remove the probability of all pools being negative from the denominator. Explicitly, this can be formulated as

46 $$\langle \pi \rangle=\mathop{\sum}_{i}i{P}_{\pi }(i)=\frac{{\sum }_{i}i{Q}_{\pi }(i)}{{\sum }_{i}{Q}_{\pi }(i)}$$

for any general *Q* ∝ *P*. If we now condition to the knowledge that at least one of the pools (true at each step) is positive, the new probability will be proportional everywhere apart for *P*(*π* = 0) = 0. We can use the previous probability as a non-normalized probability (a *Q* function) and write

47 $${\langle \pi \rangle }_{\pi \ > \ 0}=\frac{{\sum }_{i\ > \ 0}i{P}_{\pi }(i)}{{\sum }_{i\ > \ 0}{P}_{\pi }\pi (\pi=i)}=\frac{{\sum }_{i}i{P}_{\pi }(i)}{{\sum }_{i}{P}_{\pi }(i)-{P}_{\pi }(0)}=\frac{\langle \pi \rangle }{1-{P}_{\pi }(0)}=\frac{{M}^{*}m\psi }{1-{(1-\psi )}^{{M}^{*}}}$$

noting that *P*_*π*_ is normalized, ∑_*i*_*P*_*π*_(*i*) = 1. Finally, the general formula for any number of steps is

48 $$\tau (\rho,\left\{m\right\})=\frac{1}{{M}_{0}}\left(1-{\psi }_{0}+{\psi }_{0}\left({\sum }_{l=1}^{L-1}{M}_{l+1}^{*}\frac{{\psi }_{l}}{1-{(1-{\psi }_{l})}^{{M}_{l}^{*}}}\right)\right)$$

This expression can be computationally optimized and therefore provides quick solutions for any input parameters. Furthermore, we can note that

49 $$\begin{array}{rcl}1-{(1-{\psi }_{l})}^{{M}_{l}^{*}}&=&{\left(1-\left(1-{(1-\rho )}^{{M}_{l}}\right)\right)}^{{M}_{l}^{*}}={\left({(1-\rho )}^{{M}_{l}}\right)}^{{M}_{l}^{*}}\\ &=&{(1-\rho )}^{{M}_{l}{M}_{l}^{*}}={(1-\rho )}^{{M}_{0}}={\psi }_{0}\end{array}$$

by adding in the definition of *ψ* from Eq. (43). Therefore we can re-write Eq. (47) as:

50 $$\tau (\rho,\left\{m\right\})=\frac{1}{{M}_{0}}\left(1-{\psi }_{0}+\left({\sum }_{l=1}^{L-1}{M}_{l+1}^{*}{\psi }_{l}\right)\right)$$

where we sum over all steps the expected number of positive pools ${M}_{l}^{*}{\psi }_{l}$ times the number of pools at the next step necessary for each positive step-l pool. This equivalence further validates our estimation of *τ* as identical to the fully conditional estimate that could be obtained by enumerating all possible cases at each step.

#### Prevalence-based multi-dimensional design

Here, we implement a prevalence-based multi-dimensional design, expanding from a previous prevalence-based matrix (2D) design^18^. In this case, we have a hyper-cube *D*-dimensional matrix of side *L*. Therefore, the probability that at least one sample is positive in the matrix is

51 $$P(S > 0| L,D)=1-{(1-\rho )}^{{L}^{D}}$$

where *S* is the number of positive samples given the hyper-matrix parameters *L* and *D*. For this class of designs, the positive sample can be identified in a single step if at most one sample is positive. If more than one sample is positive, we can approximate that up to *S*^*D*^ extra tests are needed, allowing to formulate realistic designs in cases of unknown and possibly underestimated prevalence. In this case, our metric becomes

52 $$\tau (L,D)={\sum }_{i\ge 2}^{L-1}\,{\mbox{bmf}}(i,\rho,{L}^{D}){i}^{D}+{\mbox{bsf}}\,(L,\rho,{L}^{D}){L}^{D}$$

where bmf and bsf are respectively the mass and survival function of *L* in a binomial probability with success probability *ρ* and *L*^*D*^ total draws. For each value of *D, τ*(*L*, *D*) can easily be optimized computationally. In this case, for the optimization we start from *D* = 2 and increase *D* until we reach ${\min }_{L}\tau (L,\bar{D}-1) > {\min }_{L}\tau (L,\bar{D}) < {\min }_{L}\tau (L,\bar{D}+1)$ then we assume $\bar{D}$ to be the best dimension and ${{\min }_{L}}\tau (L,\bar{D})$ to be the optimal test per sample.

### Comparisons with existing tools

To compare the performance of BinGroup2^18^, a group testing tool optimizing four distinct pooling designs when sampling from an infinite population, with PoolPy, which require a value for *N* and *D*, we a) selected a prevalence value *ρ*, b) optimized the groups size *γ* for BinGroup2’s design “D2”^2,18^ for *ρ*, and c) defined *N* = 10*γ* and *D* = ⌈*Nρ*⌉, enabling a comparison between the two tools. In this way, the variance in the number of positives when drawing one set of *γ* elements without replacement from a population of *N* = 10*γ* with *D* = ⌈*Nρ*⌉ positives covers 90% of the variance when drawing from an infinite population with prevalence *ρ*. For each tool across a range of prevalence values *ρ*, performance was assessed in terms of test per sample and number of steps needed by the corresponding designs, as well as run time needed by the tool to generate the designs.

### Protein-ligand screen using thermal shift assay

To simulate ligand screening from a compound library with a prevalence of 0.1% positive hits, a set of 1000 samples was generated in 20% DMSO, in which one sample was randomly spiked with acetazolamide (Sigma, #A0100000) at either 0.5 or 5 mM. Then, 96 samples were randomly selected without replacement from the total set in five independent draws, which were used as testing sets for combinatorial pooling using PoolPy designs. For each draw, two pooling designs were tested on the 96 samples, one minimizing test number and one minimizing signal dilution. In each case, the 96 samples were arranged in a stock 96-well plate and then pooled according to the two designs using a Biomek i7 liquid-handling robot (Beckman Colter) following the PoolPy-generated automation method file for the corresponding design. For the scenario with two positives, two samples out of 96 were randomly spiked with either 0.5 mM acetazolamide or 5 mM brinzolamide (Sigma, #SML0216).

Ligand interaction screening was performed using thermal shift assay with the Protein Thermal Shift Dye Kit (Thermo, #4461146) as previously described^28^. Briefly, reactions were set on ice in 20 μl containing 4 μl of human carbonic anhydrase II (Sigma, #C6165) previously resuspended in PBS at 50 μM, 5 μl of Protein Thermal Shift Buffer, 2.5 μl of Protein Thermal Shift Dye, 2 μl of 10 × PBS and 2 μl of either blank (20% DMSO) or of pooled samples. Four negative controls containing blank samples were measured on each plate. Pooled samples were measured in single technical replicates. Melt curves were recorded on a QuantStudio 3 Real-Time PCR System (Applied Biosystems) following the manufacturer’s instructions. Melting temperatures were defined by identifying the peak maximum of the first derivative as provided by the instrument’s software. Positive samples were identified as having a melting temperature greater than three standard deviations above the average of the negative control samples of their corresponding plate.

### RT-qPCR testing

SARS-CoV-2 RT-qPCR testing was performed using the Luna SARS-CoV-2 RT-qPCR Multiplex Assay Kit (NEB,#E3019S) following the manufacturer’s instructions. Sets of 96 samples were prepared in 20 μl of nuclease-free water containing 50–100 ng of human genomic DNA (Applied Biosystems, #4312660). One, two or five samples were randomly spiked with 10^3^ - 10^4^ copies of SARS-CoV-2 RNA standards (ATCC, #VR-3276SD) as positive samples. Different pooling designs were chosen from the PoolPy app, and samples were then pooled accordingly before testing. RT-qPCR reactions were assembled on ice in 20 μl containing 5 μl of samples and measured on a QuantStudio 7 Flex Real-Time PCR System (Applied Biosystems) following the manufacturer’s instructions. Each measured plate contained negative and positive controls, and each samples were measured in single technical replicates. Samples were defined as positive if both the N1 and N2 regions of the SARS-CoV-2 nucleocapsid RNA were amplified with detectable Ct (below 40), as per the manufacturer’s instructions.

### Protein purification

Protein expression for ten *E. coli* TFs (PdhR, TorR, RutR, DhaR, Mlc, GlpR, MetJ, IclR, UxuR and DgoR; Supplementary Table 2) was performed using strains from the ASKA library^29^. The expression strains were inoculated at OD_600 _= 0.1 in 100 ml of Luria-Bertani (LB) medium (10 g/L tryptone, 10 g/L sodium chloride, 5 g/L yeast extract) containing 30 *μ*g/ml chloramphenicol from overnight pre-cultures. After 4 h of growth at 37 ^∘^C, protein expression was induced with 0.5 mM of IPTG and cultures were further incubated at 20 ^∘^C for 20 h. Cells were then collected by centrifugation for 20 min at 6000 × *g*, discarding of supernatant and freezing of cell pellets at − 80 ^∘^C overnight. Pellets were resuspended in 10 ml of B-PER Complete Bacterial Protein Extraction Reagent (Thermofisher, #89821) containing protease inhibitor (Roche, #11836170001) and incubated at room temperature (21 ^∘^C) for 15 min on a rotating wheel. Lysates were then clarified by centrifugation for 20 min at 6000 × *g*, and the resulting supernatants mixed with 10 ml of IMAC buffer (50 mM Tris-HCl, 500 mM NaCl, 5 % Glycerol, pH 8) containing 20 mM of imidazole and 500 μl of pre-washed HisPur Cobalt Resin (Thermofisher, #89964) and further incubated for 1 hour at room temperature on a rotating wheel. Samples were then passed through empty gravity columns (Thermofisher, #29924) and washed twice with 10 ml of IMAC buffers containing 20 and 40 mM of imidazole each, for a total of four washes. Purified proteins were eluted in 2 ml of IMAC buffer containing 500 mM of imidazole and then dialyzed overnight at 4 ^∘^C using dialysis cassettes (Sigma, #PURX60050) in protein storage buffer (50 mM Tris, 250 mM NaCl, 50 mM KCl, 10 % Glycerol, 0.5 % Tween20, pH 7.5) at a 1:500 sample-to-buffer volume ratio. Protein quality and quantity were assessed by SDS-PAGE and using a Qubit protein assay (Thermofisher, #Q33211).

### DAP-seq

DNA affinity purification sequencing (DAP-seq) was performed as previously described^30^ with minor modifications. To generate genomic libraries, genomic DNA was extracted from *E. coli* K-12 MG1655 cells grown overnight in LB medium at 37 ^∘^C using 1 OD-ml equivalent per column with the Monarch spin gDNA extraction kit (NEB, #T3010) following the manufacturer’s instructions and then fragmented to 100–300 bp by sonication. After quality control of fragment size on an agarose gel and quantification by absorbance measurement, the genomic DNA was prepared using the NEB End Repair (NEB, #E6050) and dA Tailing (NEB, #E6053) modules with 5 μg of input DNA, and then adapter-ligated by incubation for 2 hours at room temperature with pre-annealed (2 min at 96 ^∘^C followed by 1 h at room temperature) sequencing Y adapters (Adapter A and B; Supplementary Table 3) at a 1:7.5 ratio using the NEB Blunt-TA ligase (NEB, #E0367), while purifying the resulting DNA after each step using SPRIselect beads (Beckman Colter, #B23318) following manufacturer’s instructions using bead ratios of 1.8, 1.8 and 0.9, respectivelly. Adapter-ligated libraries were then pre-amplified for 10 cycles using the Phusion High-Fidelity DNA Polymerase (NEB, #M0530) with 10 ng of template DNA per 50 μl reaction using primers P1 and P2 (Supplementary Table 3) at 0.5 μM and then cleaned up with 0.9 × SPRIselect beads, yielding final gDNA libraries used in DAP-seq.

DNA-binding reactions were done in 100 μl of DAP buffer (40 mM Tris-HCl, 150 mM KCl, 10 mM MgCl2,0.01% Triton X-100, pH 7.5) containing 50 ng of adapter-ligated DNA library, 1 μg of sheared salmon sperm DNA (Thermofisher, #15632011), 10 μl of pre-washed cobalt magnetic beads (Thermofisher, #10104D) and 5 μl of 20 μM TF protein sample for TF samples or of protein storage buffer for negative controls. For pooled TF assays, equal volumes of each individual TF proteins were first pooled from 20 *μ*M stocks according to the PoolPy design, minimizing test number (four pools for ten TFs). Each single, pooled or control reactions were performed in a single technical replicate. Reactions were incubated for 1 hour at room temperature on a rotating wheel, and beads bound to protein-DNA complexes were then washed six times by magnetic separation with 200 *μ*l of DAP buffer before resuspension in 25 μl of nuclease-free water and elution by incubation at 95 ^∘^C for 10 min. After magnetic separation, the 25 *μ*l supernatants were collected and used as templates for final amplification and barcoding by PCR using the Phusion High-Fidelity DNA Polymerase and primers P5_universal and P7_barcoded (Supplementary Table 3) at 0.5 *μ*M in 50 μl reactions. All samples were then quantified using a Qubit dsDNA BR assay (Thermofisher, #Q33266) and equal amounts of each sample were mixed into a final sequencing pool, which was then concentrated using 0.9 × SPRIselect beads and cleaned up by gel extraction using the Monarch Spin DNA Gel Extraction Kit (NEB, #T1120). After quality control and quantification using a 4150 TapeStation (Agilent), the pool was sequenced on a NextSeq2000 (Illumina) using a P2 XLEAP-SBS flowcell (Illumina, #20100987) in 2 × 50 bp paired-end mode, yielding an average of 4.8 million reads per sample. DAP-seq reads were mapped to the *E. coli* K-12 U00096.3 genome assembly using Bowtie2^31^ with default parameters. Duplicate fragments were then removed using Samtools^32^ before peak calling against negative controls using MACS3^33^ in paired-end mode and default parameters, and a minimum fold enrichment of 2. Peaks shared between samples were identified using a 50% reciprocal minimum overlap, yielding a final list of peak coordinates for each single or pooled sample. The decoding of the peak presence/absence in pooled samples was performed using the multi-readout binary decoder on the PoolPy website. Previously known binding sites obtained from RegulonDB, were^34^ considered recovered when the entire binding site was within a detected peak region.

### Statistics & reproducibility

All analyses and figures were performed in Python. No data were excluded from the analyses. No statistical method was used to predetermine sample size. The DAP-seq, thermal shift assay and RT-qPCR screens were performed in single technical replicates to reflect large-scale screening conditions. To generate independent sample draws for ligand-protein interaction assays, 96 samples were randomly selected from a set of 1000, as described in the method section. For spiking of samples in additional thermal shift assays and RT-qPCR experiments, 1-5 samples per 96-well plates were randomly spiked with selected standards. All randomized selections were done using Python. The investigators were not blinded to allocation during experiments and outcome assessment.

### Reporting summary

Further information on research design is available in the Nature Portfolio Reporting Summary linked to this article.

## Supplementary information


Supplementary Information
Description of Additional Supplementary Information
Supplementary Data 1
Reporting Summary
Transparent Peer Review file


## Source data


Source Data


## Data Availability

The full benchmarking dataset containing precomputed designs and performance comparisons generated in this study is available on Zenodo under accession 18660061^19^. The raw DAP-seq sequencing data is available in the NCBI database under the bioproject number PRJNA1425967. The remaining data are available within the Supplementary Information or Source Data files. Source data are provided in this paper.

## Code availability

The PoolPy web application and tools are available at https://poolpy.trouillonlab.org or https://trouillon-lab.github.io/PoolPy. The PoolPy code used for this study is available through GitHub at https://github.com/trouillon-lab/PoolPy and on Zenodo under accession 21836249^35^.
