# Supplementary material for: Combinatorial group testing for efficient scaling across biological applications
Source: Nat Commun. 2026 Aug 25;17:9146. doi: 10.1038/s41467-026-77055-5 (PMC13522496; doi:10.1038/s41467-026-77055-5)
Supplement: Supplementary file 1 [file 41467_2026_77055_MOESM1_ESM.pdf]

Supplementary Materials

Combinatorial group testing for efficient scaling across biological applications

Lorenzo Talamanca[1] and Julian Trouillon[1*]

[1]Institute of Molecular Systems Biology, ETH Zürich, Zürich, 8093, Switzerland.

*Corresponding author. E-mail: jtrouillon@ethz.ch

Content

- Supplementary Figures 1 - 8
- Supplementary Tables 1 - 3
- Supplementary Notes 1 - 4

Additional supplementary materials

- Supplementary Data 1

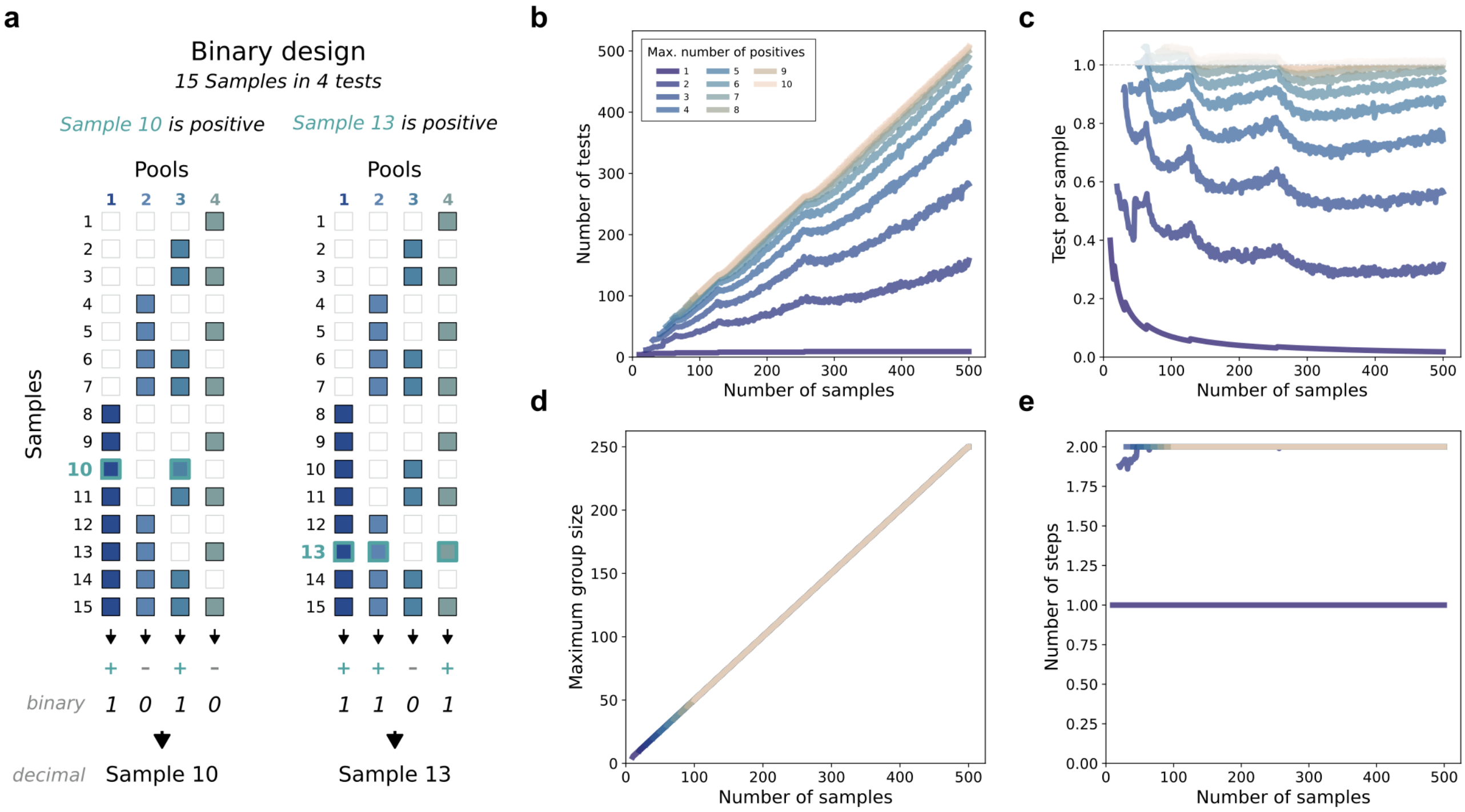

Supplementary Fig. 1: **The binary design performance decreases with increasing prevalence.** (**a**) Schematic illustration of the binary design. Two examples are shown with each a different positive sample out of 15 (shown with cyan borders). For 15 samples, the binary design makes four pools of eight samples each. The result pattern of the four pools encodes the identity of the positive sample in binary numeral system. (**b-e**) Number of total tests (**b**), number of test per sample (**c**), maximum group size (**d**) or number of steps (**e**) needed using the binary design with 1 - 10 maximum numbers of positive samples across 10 to 100 samples. Source data are provided as a Source Data file.

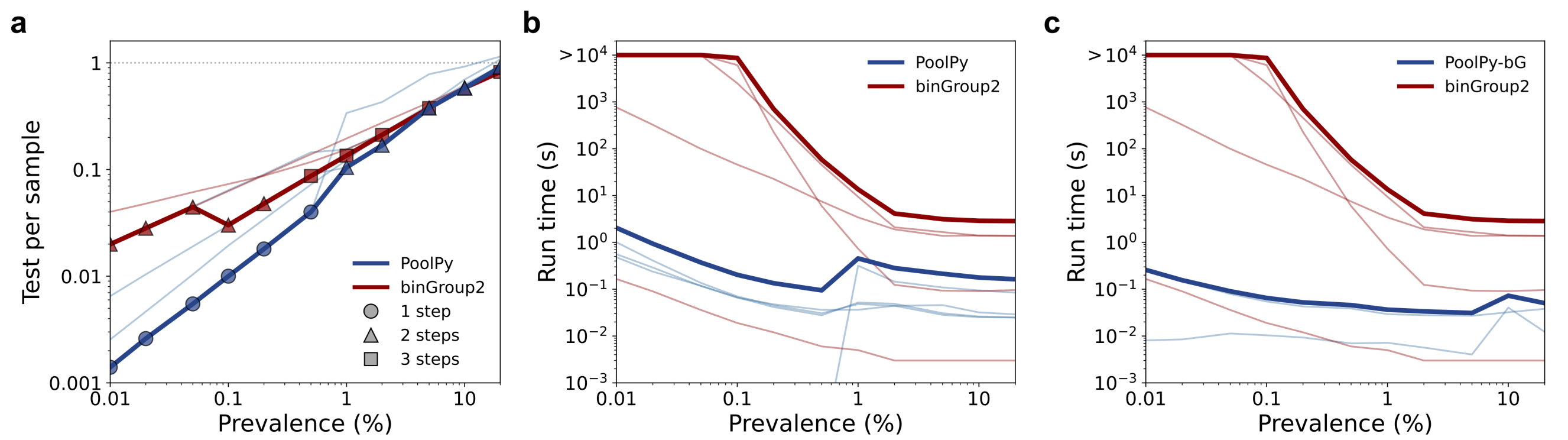

Supplementary Fig. 2: **Performance comparison of PoolPy and binGroup2.** (**a**) Test per sample needed to identify positive samples at different prevalences (0.01–20%). Individual designs are shown as faded lines and the best design of each tool is shown as a bold line. The number of steps needed by the corresponding design is indicated by the marker shape. (**b,c**) Run time needed to generate pooling designs. The bold lines denote the total run time needed to generate all individual designs shown as faded lines. Run times are shown for PoolPy's original designs (**b**), or PoolPy's implementation (PoolPy-bG) of binGroup2 prevalence-based designs (*c*). Source data are provided as a Source Data file.

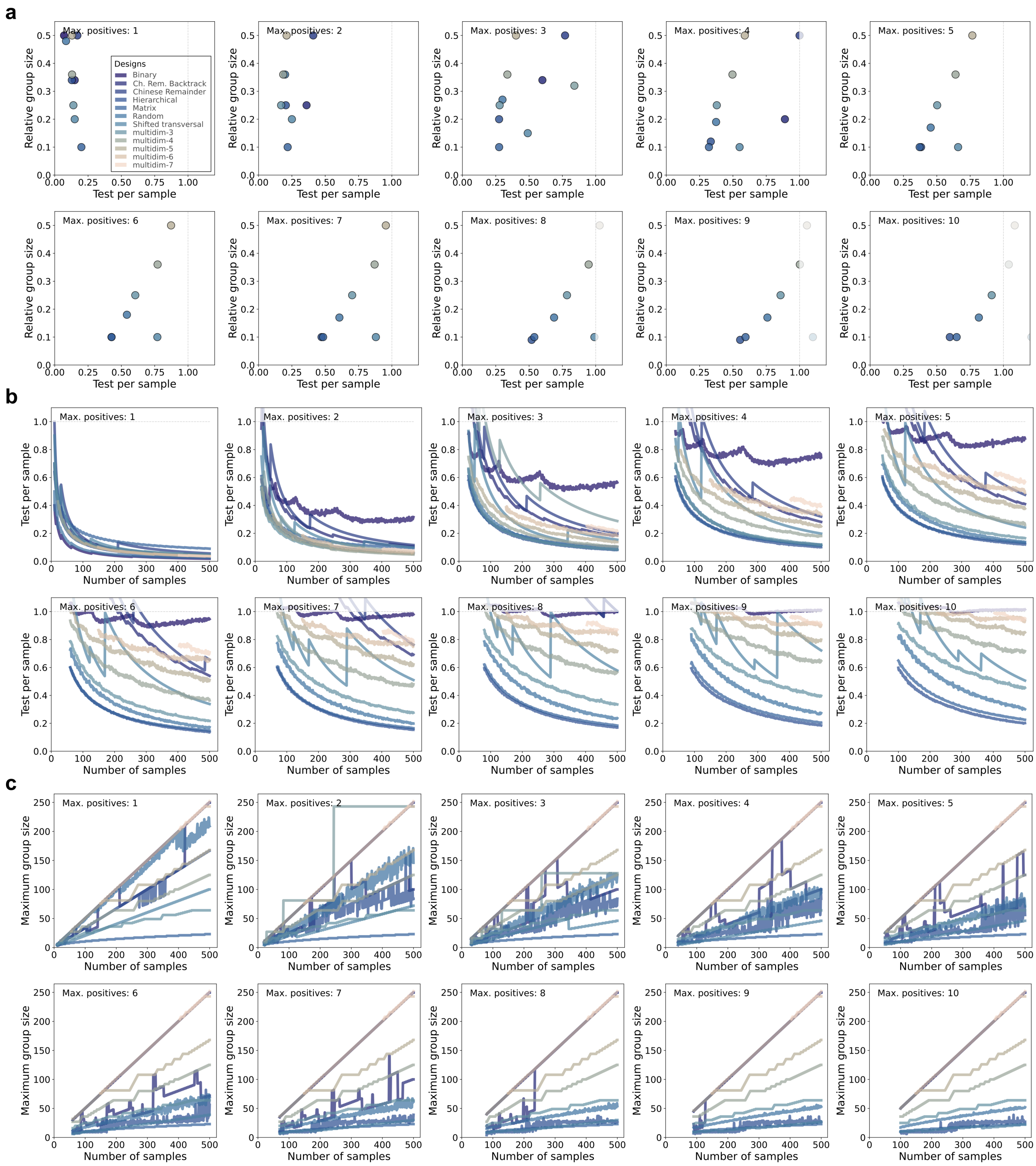

Supplementary Fig. 3: **Group testing performances and group sizes vary across prevalence values.** (**a-c**) Relation between relative group size and test per sample (**a**), and between number of samples and test per sample (**b**) or maximum group sizes (**c**) for all PoolPy designs with 1 - 10 maximum number of positive samples for either cases with 100 samples (**a**) or across varying numbers of samples (**b,c**). (**a,b**) The part where group testing becomes less efficient than individually testing each sample (above one test per sample) is grayed out. Source Data are provided as a Source Data file.

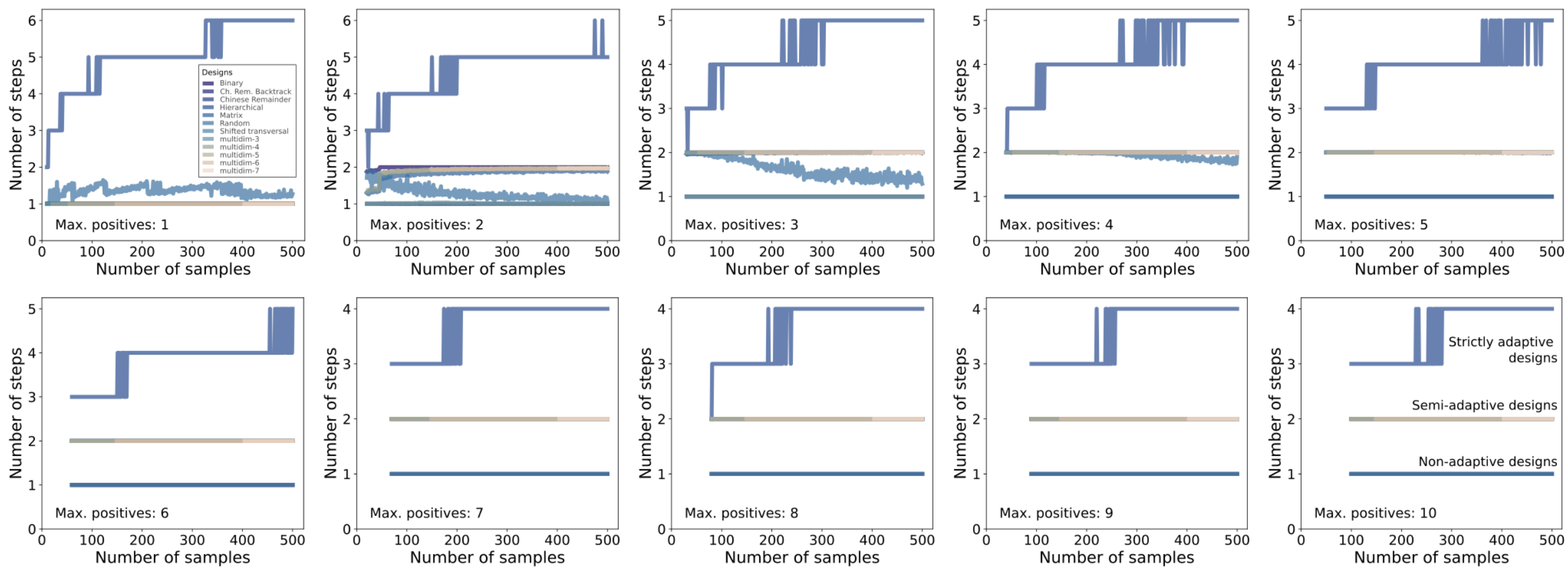

Supplementary Fig. 4: Group testing methods require varying numbers of steps. Number of steps (rounds of experiment) needed using different group testing methods to identify up to 1 - 10 positive samples across varying numbers of samples. Only methods based on the Chinese Remainder Theorem or on the shifted transversal design can identify positive samples in a single step across prevalence values (non-adaptive designs). Source data are provided as a Source Data file.

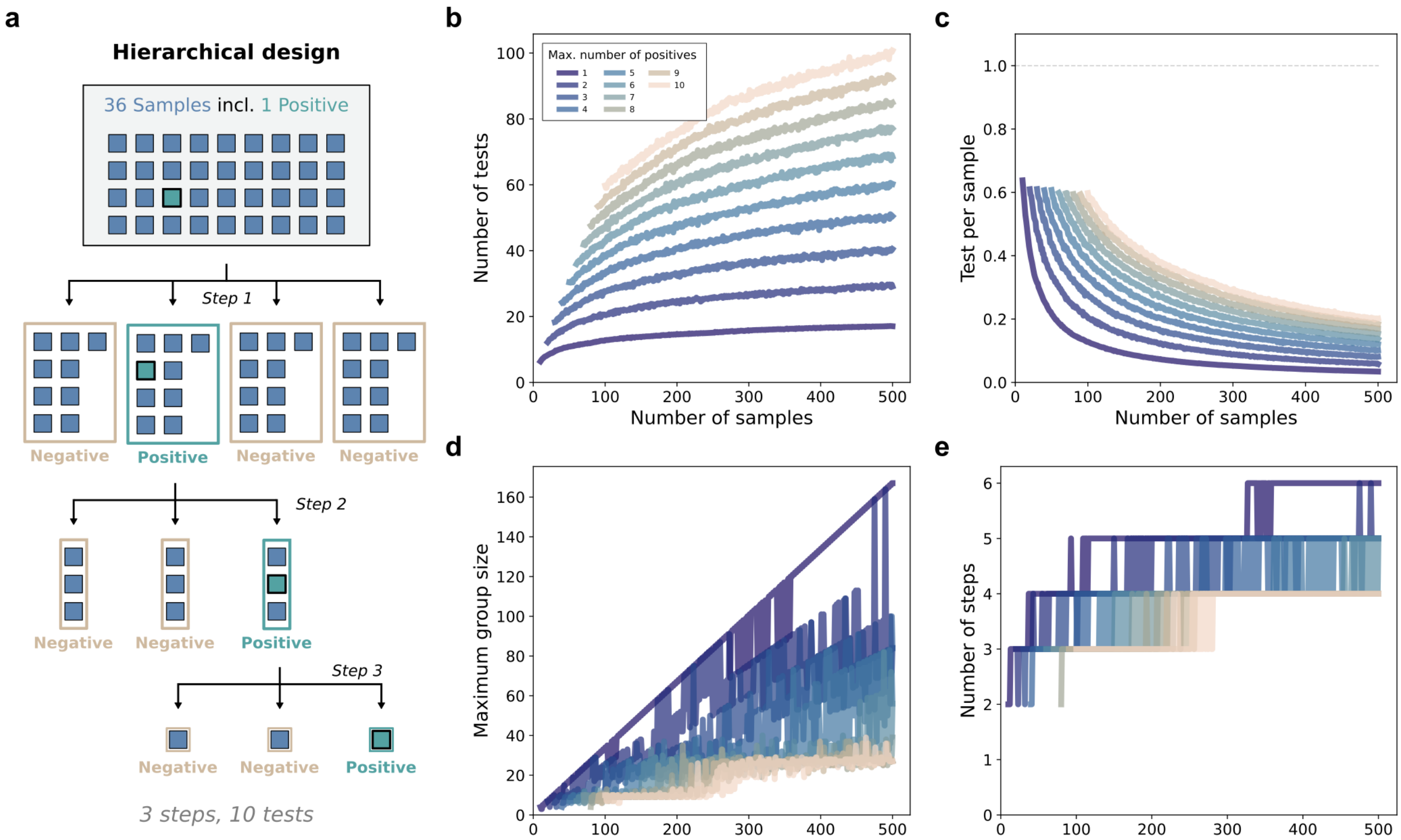

Supplementary Fig. 5: **The hierarchical method for adaptive group testing. a** Schematic illustration of the hierarchical design. In this example with 36 samples, the hierarchical design uses ten tests across three consecutive steps to identify one positive sample, shown in cyan. (**b-e**) Number of total tests (**b**), number of test per sample (**c**), maximum group size (**d**) or number of steps (**e**) needed using the hierarchical design with 1 - 10 maximum numbers of positive samples across 10 to 100 samples. Source data are provided as a Source Data file.

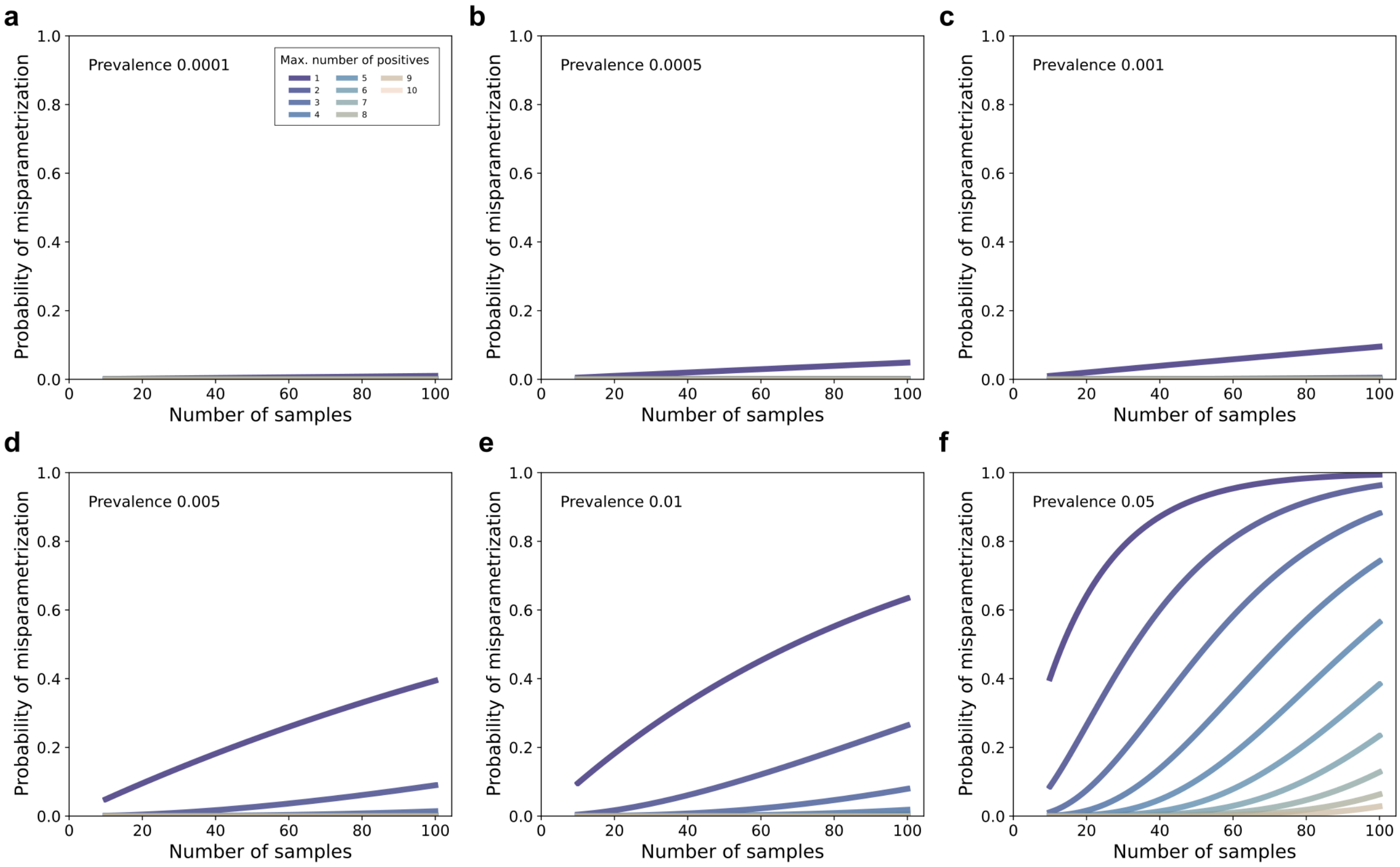

Supplementary Fig. 6: **Prevalence determines misparametrization rates based on expected number of positive samples.** (**a-f**) Probability of misparametrization (drawing more positives than the defined maximum) across six prevalence values (**a** to **f**) shown for 1 - 10 maximum numbers of expected positive samples. Source data are provided as a Source Data file.

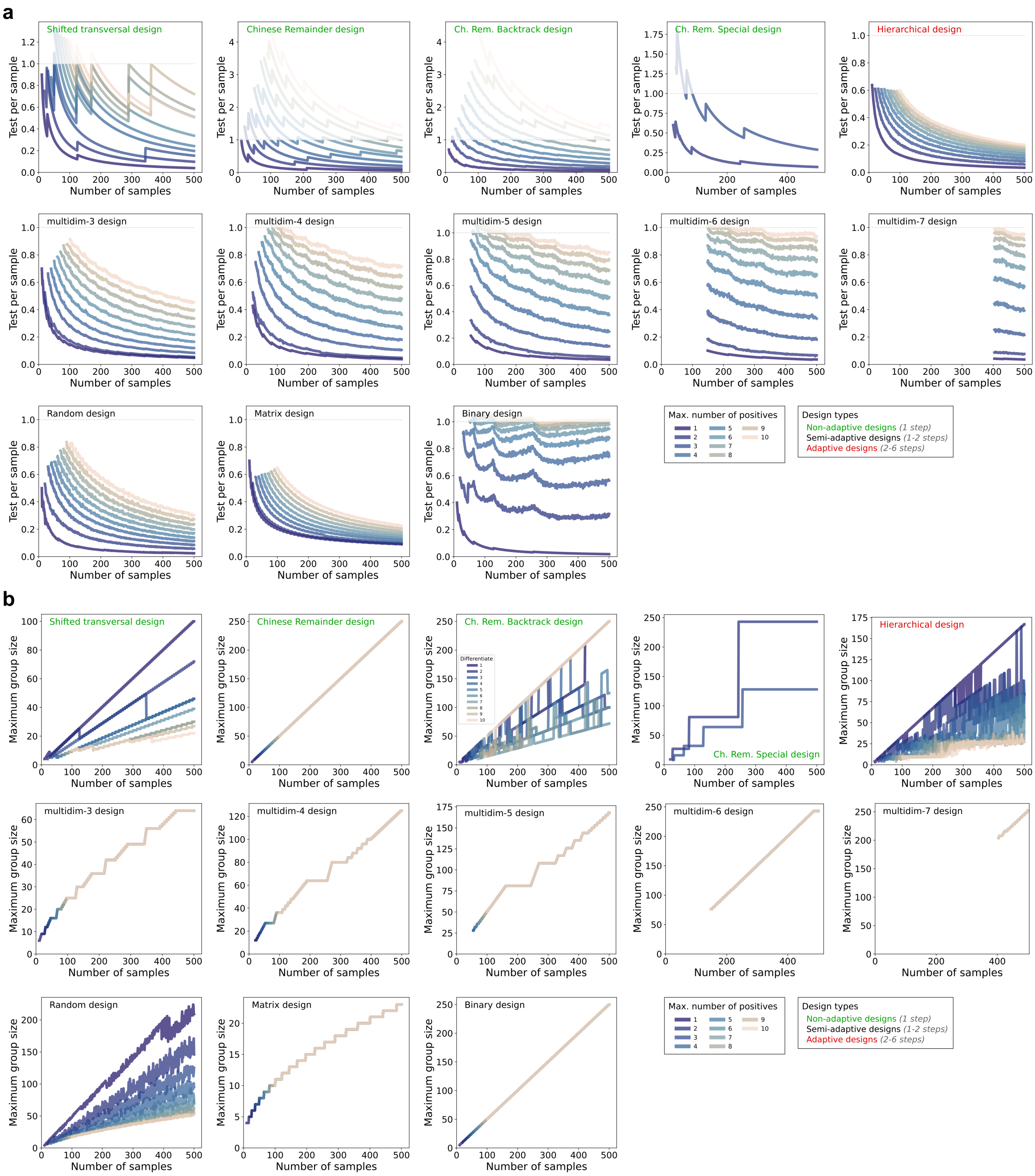

Supplementary Fig. 7: **Group testing performances across all PoolPy designs.** (**a-b**) Numbers of test per sample (**a**) and maximum group sizes (**b**) for each of the 13 possible PoolPy designs with 1 - 10 maximum numbers of positive samples across 10 to 500 samples. Source data are provided as a Source Data file.

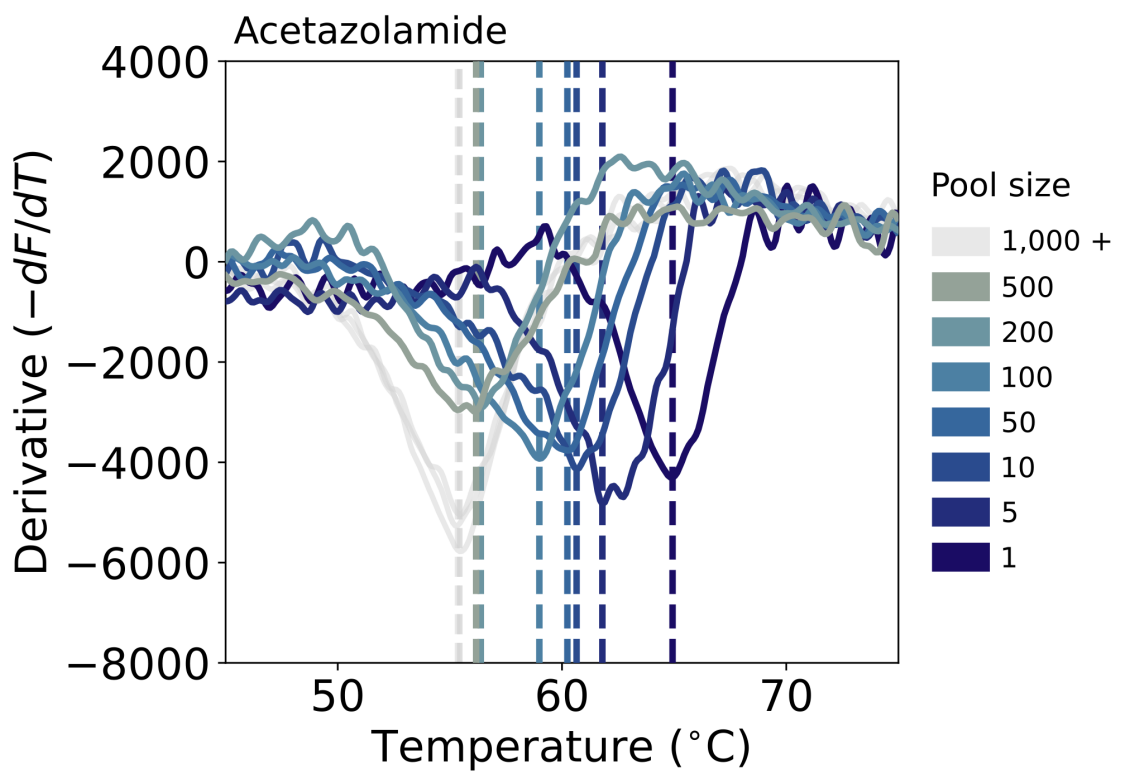

Supplementary Fig. 8: **Pool size defines signal dilution in pooled testing.** Ligand interaction screening for human carbonic anhydrase II using pools of samples that include one positive sample containing 5 mM acetazolamide, across varying numbers of sample per pool. Source data are provided as a Source Data file.

**Pooling method**	**Reducing test number**	**Minimizing group size**	**Minimizing number of steps**	**Scaling with high prevalence**	**Category**	**Ref.**
**Hierarchical**	good	good	very poor	very good	Adaptive	[1]
**Binary**	very good	very poor	poor	very poor	Semi-adaptive	
**Matrix**	very poor	very good	average	good	Semi-adaptive	[2]
**Multi-dimensional - 3**	average	good	average	good	Semi-adaptive	[3, 4]
**Multi-dimensional - 4**	average	poor	average	average	Semi-adaptive	[3, 4]
**Multi-dimensional - 5**	average	very poor	average	average	Semi-adaptive	[3, 4]
**Random**	good	average	poor	good	Semi-adaptive	[5]
**Shifted transversal**	poor	good	very good	average	Non-adaptive	[6, 7]
**Chinese Remainder**	very poor	very poor	very good	very poor	Non-adaptive	[8]
**Ch. Rem. Backtrack**	poor	average	very good	poor	Non-adaptive	[8]
**Ch. Rem. Special**	poor	poor	very good	poor	Non-adaptive	[8]

Supplementary Table 1: **Performance comparison of group testing methods supported by PoolPy.** Methods were ranked based on four criteria reflecting real-world constraints to guide user choice. The methods were classified in quintile ranks (corresponding to the five annotations in order: very poor, poor, average, good and very good) for their average value of the corresponding metric across all designs from sample set sizes of 20 to 100. For test number and group size, designs made with a maximum number of positives of one were used. For number of steps and scaling at high prevalence, the average over all designs made with maximum number of positives values of 1 - 5 was used.

TF ID	TF name	Gene ID	Known binding sites	Recovered single	Recovered pooled
A	PdhR	b0113	15	7	10
B	TorR	b0995	11	4	4
C	RutR	b1013	5	3	3
D	DhaR	b1201	0	-	-
E	Mlc	b1594	7	7	6
F	GlpR	b3423	18	2	2
G	MetJ	b3938	27	19	15
H	IclR	b4018	8	5	8
I	UxuR	b4324	8	5	5
J	DgoR	b4479	2	2	0

Supplementary Table 2: **Transcription factors binding site summary.** List of the ten *E. coli* TFs studied in this work using either single or pooled DAP-seq. The numbers of previously known binding sites as well as of recovered binding sites using either single or pooled assays are indicated.

Name	Sequence	Notes
Adaptor A	CACGACGCTCTTCCGATCT	
Adaptor B	P-GATCGGAAGAGCACACGTCTG	"P-": phosphorylation
P1	CACGACGCTCTTCCGATCT	
P2	CAGACGTGTGCTCTTCCGATC	
P5_universal	AATGATACGGCGACCACCGAGATCTACACTCTTTCCCTACACGACGCTCTTCCGATCT	
P7_barcoded	CAAGCAGAAGACGGCATACGAGATXXXXXXXXGTGACTGGAGTTCAGACGTGTGCTCTTCCGATC	"XXXXXXXX": barcode

Supplementary Table 3: **Primers used in this study.** List of adaptors and primers used in the DAP-seq protocol. Sequences are reported in the 5' to 3' direction. Primers were purchased from Microsynth AG.

Supplementary Note 1. The foundational group testing designs.

Group testing started with simple, intuitive designs (Fig. 1a). It was first introduced during World War II as a way to reduce the number of tests needed to identify soldiers with syphilis infections [9]. There, a simple two-step design was used where samples were pooled and tested in small groups, and when a group came back positive, each sample in it were tested individually in a second step (**a**). An improvement of this method in terms of number of tests was made in the hierarchical design [1], where a more progressive step-by-step approach is used (**b**). While both designs reduce the number of tests significantly, they require multiple steps, making them less attractive when quick results are needed. The Matrix design offers another intuitive approach by arranging samples in a two-dimensional grid [2]. Each row and each column of the grid is pooled and tested as a group, so that a positive result in a specific row and column identifies the corresponding positive sample in a single step (**c**). However, this design is particularly sensitive to increase in prevalence, as additional steps are necessary if more than one positive sample is present in the tested set. These designs illustrate a common trade-off in group testing: fewer testing steps, as in the matrix design, usually come at the cost of flexibility when multiple positives are present, while multi-step methods, such as the hierarchical design, handle several positives more reliably but require more rounds of testing. Some modern non-adaptive methods, however, can resolve multiple positive samples in one step efficiently across prevalence values, partially bridging this gap.

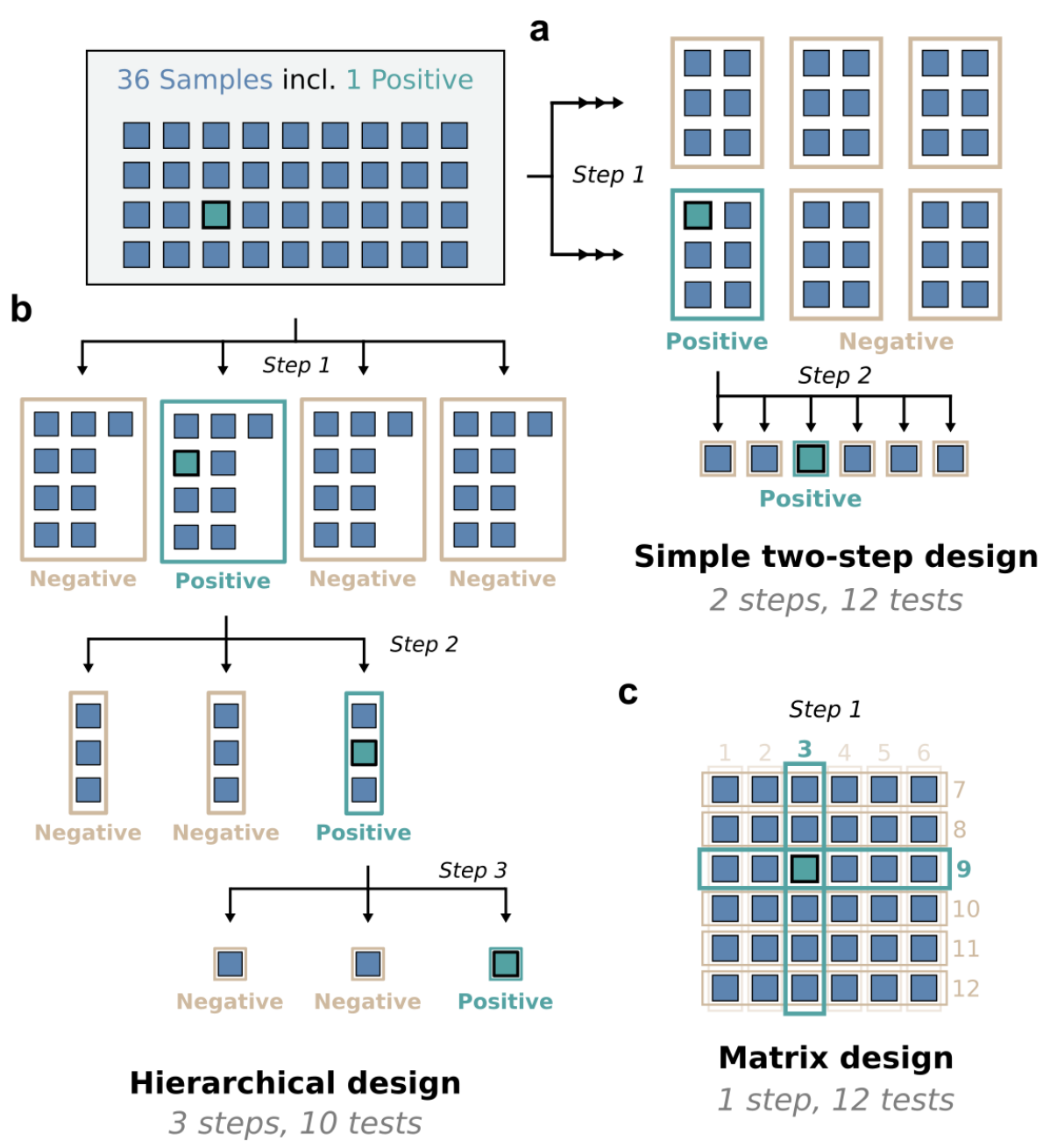

Schematic illustration of simple pooling designs. In this example, one positive sample has to be identified out of 36 (top left). Three possible designs are illustrated, using the simple two-step (**a**), hierarchical (**b**) or matrix (**c**) designs.

Supplementary Note 2. Methods implemented in PoolPy.

PoolPy supports multiple conceptually-different group testing algorithms, which can be classified based on two properties: (i) whether their designs depend on a maximum number of positives (differentiate-dependent methods) or not (differentiate-independent methods), and (ii) whether they can identify positive samples in a single step consistently (non-adaptive methods), in some cases (semi-adaptive methods), or always require multiple steps (adaptive methods). The supported methods are as follows:

Differentiate-dependent methods

These methods adapt their designs across expected prevalence values, even with fixed numbers of samples, enabling them to scale better with larger numbers of positive samples.

- **Random**: A semi-adaptive method that pools samples at random according to user-defined constraints. This method might require a second step of validation testing to resolve ambiguities.
- **Shifted Transversal Design** (STD): A non-adaptive method based on prime numbers and modular arithmetic. Each sample is assigned to pools according to shifted transversal patterns that guarantee unique identification up to a maximum number of positive samples. This method can consistently identify positive sample(s) in a single step when prevalence assumptions are met.
- **Chinese Remainder methods**: Three non-adaptive methods based on the Chinese Remainder Theorem which encode sample identities using modular congruences across multiple pools. PoolPy supports a standard variant, a backtracking variant (backtrack) that resolves ambigous cases by exploring compatible solutions and a special variant (special) optimized for 2 and 3 maximum number of positive samples. These methods can consistently identify positive sample(s) in a single step when prevalence assumptions are met.
- **Hierachical**: A strictly adaptive method that iteratively splits the set of possible positive samples. Positive pools are subdivided in subsequent steps until positive samples are identified. This method consistently requires multiple steps.

Differentiate-independent methods

These methods generate designs that do not depend on an expected prevalence value. They can show very good performances at low prevalence, but should be used with caution if multiple positive samples are expected.

- **Matrix**: A semi-adaptive method where samples are arranged in a two-dimensional grid, with each row and each column forming a pool. This method might require a second step of validation testing to resolve ambiguities with more than a single positive sample.

- **Multidimensional methods**: A set of semi-adaptive methods where samples are arranged in higher-dimensional matrices (3D, 4D, 5D, 6D and 7D are implemented in PoolPy), where each coordinate along each dimension corresponds to a pool. These methods might require a second step of validation testing to resolve ambiguities with more than a single positive sample.
- **Binary**: A semi-adaptive method where samples are assigned to pools according to a binary code, maximizing information per test. This method might require a second step of validation testing to resolve ambiguities with more than a single positive sample.

Prevalence-based designs

Additionally, PoolPy supports two prevalence-based designs, which are implementation of conceptual methods from binGroup2 [10]. Unlike other PoolPy designs, these designs do not take into account the number of samples and rather only depend on an expected prevalence value.

- **Prevalence-based hierarchical**: A strictly adaptive method that iteratively splits the set of possible positive samples. PoolPy's analytical implementation of this design finds the optimal pool size and number of steps for a given prevalence.
- **Prevalence-based multidimensional**: A set of semi-adaptive methods where samples are arranged in N-dimensional matrices, where each coordinate along each dimension corresponds to a pool. PoolPy's analytical implementation of this design finds the optimal numbers of pools and dimensions for a given prevalence.

Supplementary Note 3. The logistical constraints of group testing.

For simplicity, we refer to samples exhibiting the particular property detected by the test used as *positive*, and to their proportion within the entire set of possible samples as the *prevalence*. Across biomedical fields, various terms may be used for these two concepts; they are interchangeable and fully compatible with PoolPy.

Prevalence. The performance of combinatorial group testing designs is highly affected by prevalence. At very low prevalence, group testing can greatly reduce the number of tests, with cost reductions as high as 98%, while higher prevalence reduces its efficiency [11], with marginal reductions above a prevalence of 10%. Using sample sets that contain more positive samples than a defined threshold (the maximum number of positives) can lead to inconclusive results, mostly with non-adaptive differentiate-dependent designs. Thus, it is beneficial to estimate the prevalence in the tested population *a priori* to choose an appropriate design. To accommodate this constraint, PoolPy's Prevalence section offers a tool to estimate the chance of misparametrization (i.e. choosing a threshold too low) and choose appropriate threshold values and pooling parameters to match the expected prevalence.

Differentiate-dependent methods adapt their design based on the expected maximum number of positive samples, and typically scale well with increased prevalence. Conversely, while differentiate-independent methods show attractive performance with at most one positive sample, their performance drastically decreases with more positives, often leading to inconclusive results in these cases.

Combinatorial group testing tends to lose its efficacy at higher prevalence. Indeed, for any given prevalence ρ we can have a lower bound on the information in bits encoded in a system with N samples:

$$I \geq \binom{N}{\rho N} = \frac{N!}{(\rho N)!((1-\rho)N)!}. \tag{1}$$

This value is a lower bound as it assumes that exactly ρN samples are positive, while in experimental settings this is generally unknown. Using Stirling's approximation

$$N! \sim \sqrt{2\pi N}\left(\frac{N}{e}\right)^N \tag{2}$$

we can write assuming $\rho N \in \mathbb{N}$:

$$I(\rho) \geq \frac{\sqrt{2\pi N}\left(\frac{N}{e}\right)^N}{\sqrt{2\pi(\rho N)}\left(\frac{(\rho N)}{e}\right)^{(\rho N)}\sqrt{2\pi((1-\rho)N)}\left(\frac{((1-\rho)N)}{e}\right)^{((1-\rho)N)}}. \tag{3}$$

This expression recapitulates the limit of low prevalence $\rho = 1/N$ with $I(\rho) \geq N$ where we find methods that need $\lceil \log_2(N) \rceil$ tests. Using this formula, we can estimate the upper limit of prevalence, $\rho = 1/2$.

$$I(1/2) \geq \frac{\left(\frac{N}{e}\right)^N}{\left(\frac{(N/2)}{e}\right)^N} = 2^N. \tag{4}$$

In this case, at least $\lceil \log_2(2^N) \rceil = N$ tests are needed, rendering the combinatorial pooling approach futile. It is important to note that this information limit is reached for practical purposes before $\rho = 1/2$ prevalence.

Turnaround time. Adaptive group testing designs require multiple steps, such as the hierarchical method [1], where the outcome of one round of testing determines how the next round has to be set up. While these designs often require low overall number of tests, the total time until results are available is increased due to the multiple steps. Semi-adaptive designs can identify positive samples in a single step with low prevalence but often require a second validation step if more than one positive sample is present. Lastly, non-adaptive designs can consistently identify positive samples in a single step. All non-adaptive designs implemented in PoolPy rely on a differentiate-dependent method to allow design adaptation to higher prevalence values. In use cases where fast turnaround time is essential, non-adaptive methods that always require a single step may be preferable despite the fact that they may require more tests than adaptive designs.

Group size limit. Practical limits exist on how many individual samples can be pooled without loss of sensitivity, which mostly depend on the nature of the test being used. This is often referred to as the *signal dilution effect* in biomedical applications where the use of very large pools can increase the risk of false negatives due to increased dilution. Depending on applications, a group size limit can be defined to guide the choice of appropriate group testing designs, as some designs use relatively large group sizes. This is entirely application-specific and depends on assay sensitivity.

With increased relative group sizes, the number of times that each sample is tested can increase as well. This represents a second parameter depending on group definition that can affect applicability. In cases where the amount of sample is limiting, users can use PoolPy to choose designs that use small group sizes to reduce sample usage.

Assay type. PoolPy supports any assay that is compatible with group testing, which can typically be described as single- or multi-readout. Single-readout assays have a single binary outcome, such as infection testing (positive/negative) or ligand-target interaction screening (interacting/not interacting). Multi-readout assays yield multiple outcomes also classifiable in a binary manner. They include complex molecular profiling methods relying on omics measurements where each measured unit (e.g. each

gene, protein, binding event ...) is considered as an individual outcome. In the case of multi-readout assays, the choice of pooling design applies to all outcomes, and should thus be selected based on the most stringent pooling parameters out of all possible outcomes. For multi-readout assays with complex data, such as mass spectrometry- or sequencing-based assays, all data analysis, such as normalization or batch effect correction, should be done first following standard procedures in the corresponding field, while using the final processed results as input for the pooled decoding analysis done by PoolPy.

Supplementary Note 4. PoolPy user guide.

PoolPy is provided as a web interface (https://poolpy.trouillonlab.org) where users can design and compare group testing strategies under defined constraints. The web interface comprises five sections with distinct functions, as follows:

- **Methods Comparison**: The main section of PoolPy, where users can obtain direct comparisons of methods performance and download group testing designs for their applications based on a number of samples and a maximum number of positives.
- **Decoder**: This section provides a tool for decoding results from a group testing experiment. It takes as input the design used and the results for each group. The decoder also works for multi-readout assays when providing a corresponding readout file, for both binary or continuous readouts.
- **Prevalence**: This section provides a tool for selecting design parameters, such as an expected maximum number of positives and a number of batches, based on the estimated prevalence and a misparametrization rate threshold.
- **Automation**: This section provides a tool for generating method files for liquid-handling robots to follow the pooling scheme of a group testing design.
- **Help**: This section describes the methods and metrics used in the other sections.

It is also possible to use PoolPy locally without using the web interface, following the instructions in the PoolPy GitHub repository (https://github.com/trouillon-lab/PoolPy). Local use might be required in cases that are computationally intensive, such as to generate a design with the random method or to get a full performance summary table for cases that were not precomputed. However, the vast majority of use cases are covered by the web interface, which has precomputed designs across >100,000 conditions, and can generate design on the fly for any numbers of samples and positives for 11 out of 12 methods. Here, we provide a step-by-step guide on how to use the different sections of the PoolPy web interface to perform a group testing experiment.

PoolPy supports both single-readout assays with binary outcomes, such as diagnostic infection testing or ligand–target interaction screening, and multi-readout experiments, including mass spectrometry- or sequencing-based molecular profiling approaches. In both cases, the samples can be pooled following the generated PoolPy designs. For multi-readout assays, the highest expected prevalence value should be used to choose pooling parameters.

1. Determine pooling parameters based on prevalence (optional)

Since many group testing methods adapt their design to an expected number of positive samples, it is important to estimate the prevalence, or hit rate, among the tested samples before choosing a design. This estimated prevalence can then be used to define the pooling parameters. This can be done in the Prevalence section of the PoolPy web

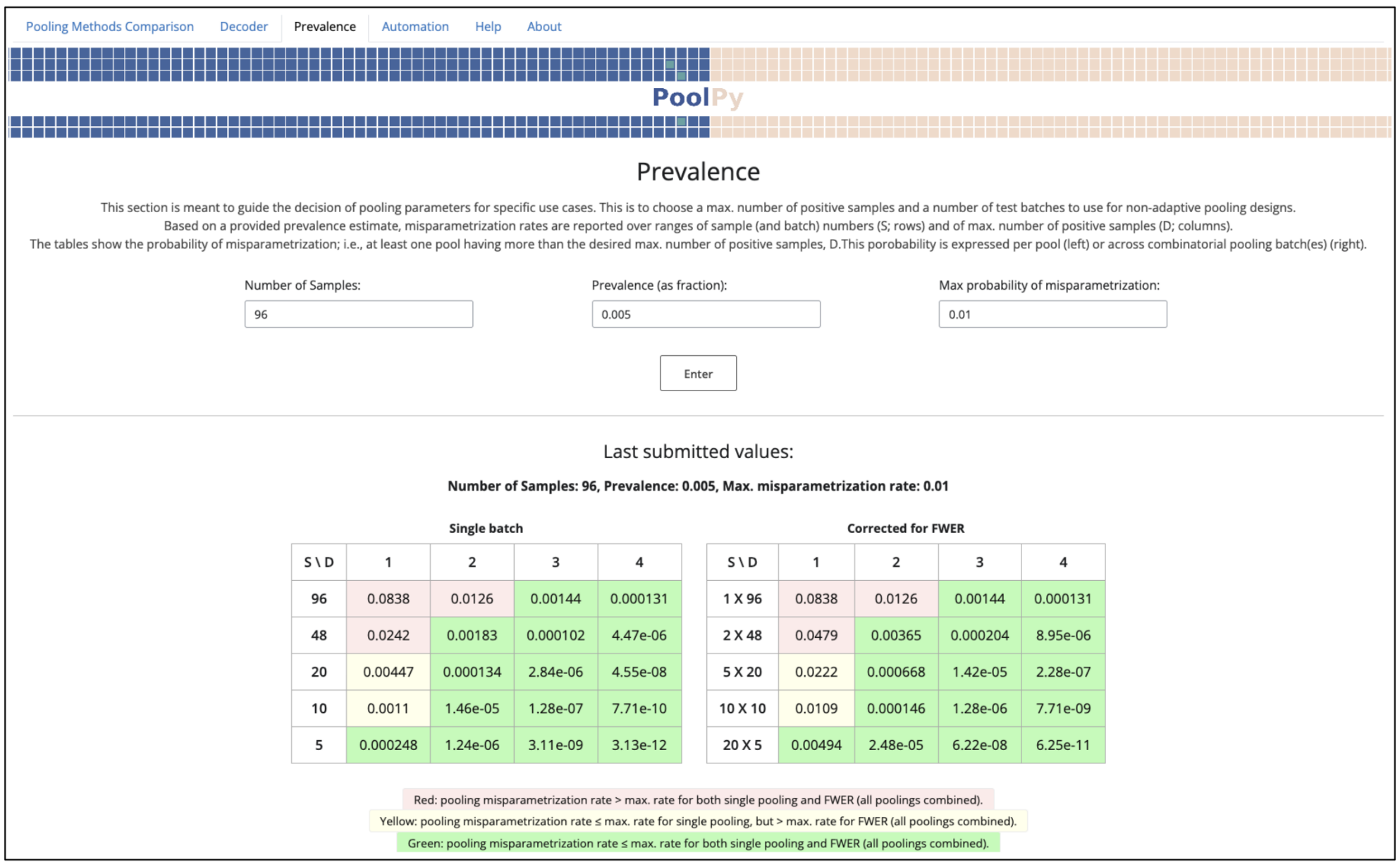

Screenshot 1: Pooling parameter determination using the PoolPy Prevalence section.

interface (Screenshot 1). There, users can input their test conditions (the number of samples, the estimated prevalence, and the maximum acceptable misparametrization rate) to get the probability of having more positive samples than the defined maximum number of positives. The consequences of which differ between methods and pooling parameters, ranging from not affecting the number of tests needed, requiring a few additional tests, to getting inconclusive results.

In the example shown in Screenshot 1, the probability of getting more positives than the threshold is shown for a case with 96 samples with an estimated prevalence of 0.5% across different numbers of batches and maximum numbers of positive samples (*differentiate*). To keep this probability below 1%, several strategies are possible in this example, such as performing a pooling experiment with all samples and a *differentiate* value of 3, or splitting the samples into two batches of 48 samples each and a *differentiate* value of 2. With this overview, users can decide on viable combinations of batch number and *differentiate* values to use to design their pooling experiment in the PoolPy Methods Comparison section.

2. Compare and choose group testing design

With a defined number of samples and maximum number of positive samples, users can compare the methods supported by PoolPy across different key performance indicators using the Methods Comparison section. In this section, precomputed performance summary tables are available for a large range of numbers of samples. In case a precomputed performance summary table is not available, specific designs can still be generated and downloaded for 11 out of 12 methods for any number of samples and positives.

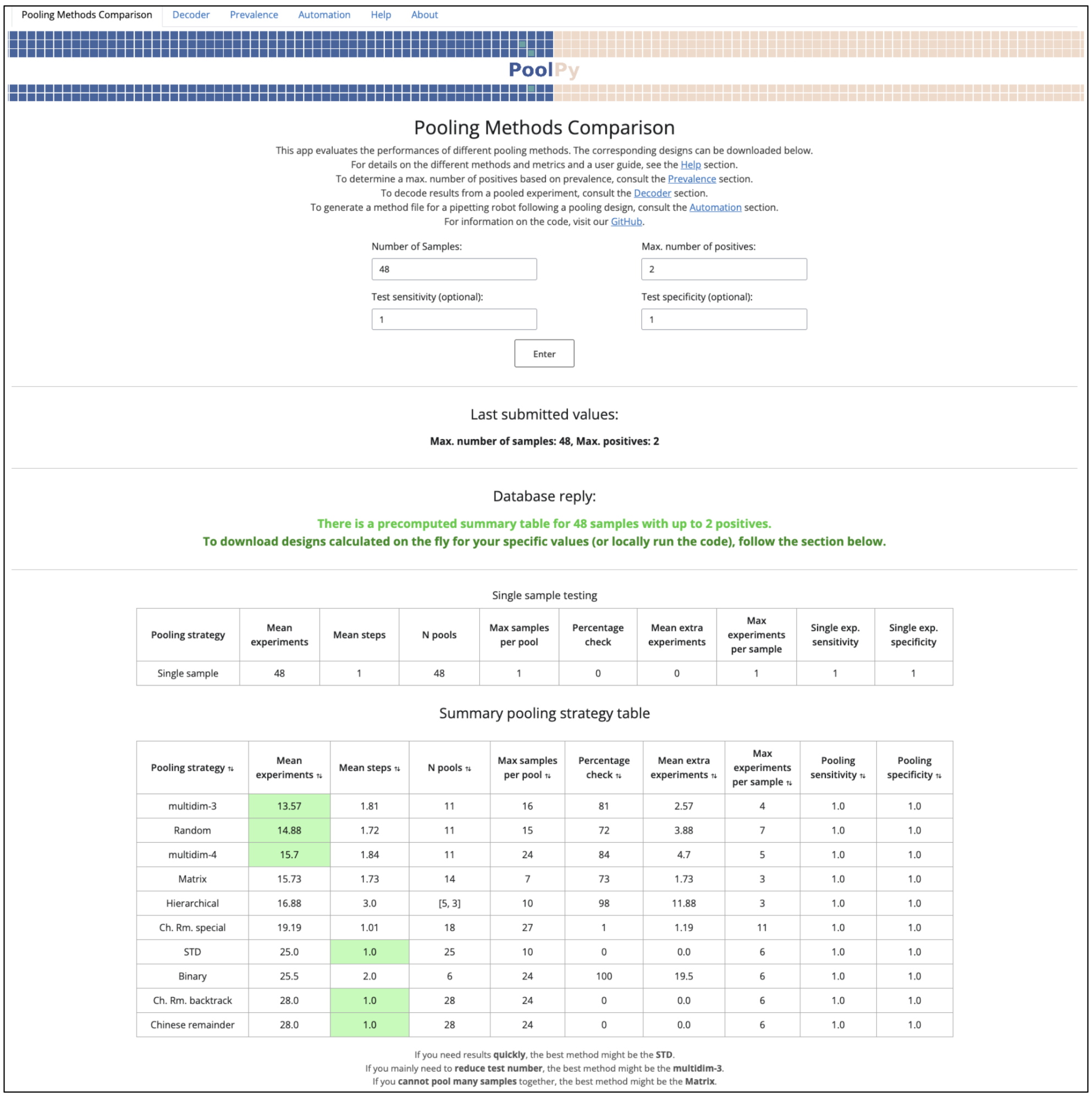

Pooling Methods Comparison | Decoder | Prevalence | Automation | Help | About

PoolPy

Pooling Methods Comparison

This app evaluates the performances of different pooling methods. The corresponding designs can be downloaded below.
For details on the different methods and metrics and a user guide, see the Help section.
To determine a max. number of positives based on prevalence, consult the Prevalence section.
To decode results from a pooled experiment, consult the Decoder section.
To generate a method file for a pipetting robot following a pooling design, consult the Automation section.
For information on the code, visit our GitHub.

Number of Samples: 48

Max. number of positives: 2

Test sensitivity (optional): 1

Test specificity (optional): 1

Enter

Last submitted values:

Max. number of samples: 48, Max. positives: 2

Database reply:

There is a precomputed summary table for 48 samples with up to 2 positives.
To download designs calculated on the fly for your specific values (or locally run the code), follow the section below.

Single sample testing

Pooling strategy	Mean experiments	Mean steps	N pools	Max samples per pool	Percentage check	Mean extra experiments	Max experiments per sample	Single exp. sensitivity	Single exp. specificity
Single sample	48	1	48	1	0	0	1	1	1

Summary pooling strategy table

Pooling strategy ⇅	Mean experiments ⇅	Mean steps ⇅	N pools ⇅	Max samples per pool ⇅	Percentage check ⇅	Mean extra experiments ⇅	Max experiments per sample ⇅	Pooling sensitivity ⇅	Pooling specificity ⇅
multidim-3	13.57	1.81	11	16	81	2.57	4	1.0	1.0
Random	14.88	1.72	11	15	72	3.88	7	1.0	1.0
multidim-4	15.7	1.84	11	24	84	4.7	5	1.0	1.0
Matrix	15.73	1.73	14	7	73	1.73	3	1.0	1.0
Hierarchical	16.88	3.0	[5, 3]	10	98	11.88	3	1.0	1.0
Ch. Rm. special	19.19	1.01	18	27	1	1.19	11	1.0	1.0
STD	25.0	1.0	25	10	0	0.0	6	1.0	1.0
Binary	25.5	2.0	6	24	100	19.5	6	1.0	1.0
Ch. Rm. backtrack	28.0	1.0	28	24	0	0.0	6	1.0	1.0
Chinese remainder	28.0	1.0	28	24	0	0.0	6	1.0	1.0

If you need results **quickly**, the best method might be the **STD**.
If you mainly need to **reduce test number**, the best method might be the **multidim-3**.
If you **cannot pool many samples** together, the best method might be the **Matrix**.

Screenshot 2: Comparing and selecting group testing design using PoolPy.

Following the suggestions from the Prevalence section above, we show an example of a method comparison for 48 samples with a *differentiate* value of 2 given by PoolPy (Screenshot 2). In these conditions, different designs are suggested based on different prioritized constraints. To minimize turnaround time, test number or sample dilution, the shifted transversal, the multidim-3 and the matrix designs are suggested, respectively. A summary table is given that details all values for these key metric. Based on these values and suggestions, users can decide on a design of choice and can directly download the corresponding design file from that section. The design file is a table indicating in which pool(s) each sample has to be added.

3. Generate automation file for pipetting robot (optional)

Although the design files can be used directly from the previous section for manual pooling of samples, PoolPy also has an Automation section allowing users to generate method files for pipetting robots to automatize the pooling of samples. In this section,

users can input a design file obtained from the Methods Comparison section (or from the PoolPy python code) and directly download method files for pipetting robots from several of the main manufacturers. The downloaded method file can then be used directly on the corresponding robot to perform the pooling of the samples following the selected design.

4. Decode pooling experiment results

After performing the sample pooling and corresponding experiment, users can decode the results of their tests on the Decoder section of PoolPy. There, users can input their design file obtained from the Methods Comparison section (or from the PoolPy python code) and write their readout, i.e. which pools were positive (or any other binary characteristic of interest for binary readouts) in their experiment, or the continuous results of each pool for quantitative decoding. The decoder then outputs the identity of the positive sample(s), or their decoded continuous values, based on the pooled results. In a successful pooling experiment, the decoder will identify the exact set of positive samples. In some cases, e.g. if the number of positive samples exceeds the defined maximum number of positives set by the user, the decoder might not be able to identify the exact set of positive samples, but will rather output a list of possible samples that contains the positive ones.

PoolPy has both a standard binary decoder and a LASSO-based continuous decoder for compressed sensing, enabling combinatorial testing for genome-scale molecular profiling assays with complex quantitative readouts. For these multi-readout assays, users can upload a readout file that contains either the list of positive pools or the continuous signal value of all pools for each individual readout (one per line). PoolPy will then decode each readout and output a result file with either the identity of the positive samples (for binary readouts) or the quantitative signal contribution of each sample (for continuous readouts) for each specified readout (e.g. each gene in transcriptomics).

Supplementary references

[1] Hou, P., Tebbs, J.M., Bilder, C.R., McMahan, C.S.: Hierarchical group testing for multiple infections. Biometrics **73**(2), 656–665 (2017)

[2] Du, D.-Z., Hwang, F.K.M.: Combinatorial group testing and its applications. World Scientific **12** (1999)

[3] Mutesa, L., Ndishimye, P., Butera, Y., Souopgui, J., Uwineza, A., Rutayisire, R., Ndoricimpaye, E.L., Musoni, E., Rujeni, N., Nyatanyi, T., *et al.*: A pooled testing strategy for identifying sars-cov-2 at low prevalence. Nature **589**(7841), 276–280 (2021)

[4] Barillot, E., Lacroix, B., Cohen, D.: Theoretical analysis of library screening using a n-dimensional pooling strategy. Nucleic Acids Research **19**(22), 6241–6247 (1991)

[5] Bruno, W.J., Knill, E., Balding, D.J., Bruce, D.C., Doggett, N.A., Sawhill, W.W., Stallings, R.L., Whittaker, C.C., Torney, D.C.: Efficient pooling designs for library screening. Genomics **26**(1), 21–30 (1995)

[6] Thierry-Mieg, N.: A new pooling strategy for high-throughput screening: the shifted transversal design. BMC bioinformatics **7**, 1–13 (2006)

[7] Xin, X., Rual, J.-F., Hirozane-Kishikawa, T., Hill, D.E., Vidal, M., Boone, C., Thierry-Mieg, N.: Shifted transversal design smart-pooling for high coverage interactome mapping. Genome research **19**(7), 1262–1269 (2009)

[8] Eppstein, D., Goodrich, M.T., Hirschberg, D.S.: Improved combinatorial group testing algorithms for real-world problem sizes. SIAM Journal on Computing **36**(5), 1360–1375 (2007)

[9] Dorfman, R.: The detection of defective members of large populations. The Annals of mathematical statistics **14**(4), 436–440 (1943)

[10] Bilder, C.R., Hitt, B.D., Biggerstaff, B.J., Tebbs, J.M., McMahan, C.S.: bingroup2: statistical tools for infection identification via group testing. The R journal **15**(4), 21 (2024)

[11] Aldridge, M., Johnson, O., Scarlett, J., *et al.*: Group testing: an information theory perspective. Foundations and Trends in Communications and Information Theory **15**(3-4), 196–392 (2019)
