# Supplementary material for: Combinatorial group testing for efficient scaling across biological applications
Source: Nat Commun. 2026 Aug 25;17:9146. doi: 10.1038/s41467-026-77055-5 (PMC13522496; doi:10.1038/s41467-026-77055-5)
Supplement: Supplementary file 2 [file 41467_2026_77055_MOESM2_ESM.pdf]

Description of Additional Supplementary Files

File Name: Supplementary Data 1
Description: This dataset contains all identified peaks for single and pooled DAP-seq experiments, as well as the pooled decoded results from PoolPy.
