# Supplementary material for: Combinatorial group testing for efficient scaling across biological applications
Source: Nat Commun. 2026 Aug 25;17:9146. doi: 10.1038/s41467-026-77055-5 (PMC13522496; doi:10.1038/s41467-026-77055-5)
Supplement: Supplementary file 3 [file 41467_2026_77055_MOESM4_ESM.pdf]

nature portfolio

Corresponding author(s): Julian Trouillon

Last updated by author(s): Aug 11, 2026

Reporting Summary

Nature Portfolio wishes to improve the reproducibility of the work that we publish. This form provides structure for consistency and transparency in reporting. For further information on Nature Portfolio policies, see our Editorial Policies and the Editorial Policy Checklist.

Statistics

For all statistical analyses, confirm that the following items are present in the figure legend, table legend, main text, or Methods section.

n/a	Confirmed	
☐	☒	The exact sample size (*n*) for each experimental group/condition, given as a discrete number and unit of measurement
☐	☒	A statement on whether measurements were taken from distinct samples or whether the same sample was measured repeatedly
☐	☒	The statistical test(s) used AND whether they are one- or two-sided<br>*Only common tests should be described solely by name; describe more complex techniques in the Methods section.*
☐	☒	A description of all covariates tested
☐	☒	A description of any assumptions or corrections, such as tests of normality and adjustment for multiple comparisons
☐	☒	A full description of the statistical parameters including central tendency (e.g. means) or other basic estimates (e.g. regression coefficient) AND variation (e.g. standard deviation) or associated estimates of uncertainty (e.g. confidence intervals)
☐	☒	For null hypothesis testing, the test statistic (e.g. *F*, *t*, *r*) with confidence intervals, effect sizes, degrees of freedom and *P* value noted<br>*Give P values as exact values whenever suitable.*
☐	☒	For Bayesian analysis, information on the choice of priors and Markov chain Monte Carlo settings
☐	☒	For hierarchical and complex designs, identification of the appropriate level for tests and full reporting of outcomes
☒	☐	Estimates of effect sizes (e.g. Cohen's *d*, Pearson's *r*), indicating how they were calculated

Our web collection on statistics for biologists contains articles on many of the points above.

Software and code

Policy information about availability of computer code

Data collection	Pooling performance data was collected using the PoolPy code available at https://github.com/trouillon-lab/PoolPy.
Data analysis	The designing of pooled assay as well as decoding of the pooled result for TSA, RT-qPCR and DAP-seq were performed on the PoolPy web app available at https://trouillon-lab.github.io/PoolPy. DAP-seq reads were mapped using Bowtie2 v2.5.2 and de-duplicated using Samtools v1.19.2. Peak calling was done using MACS3 v3.0.3. The PoolPy web application and tools are available at https://poolpy.trouillonlab.org or https://trouillon-lab.github.io/PoolPy. The PoolPy code used for this study is available through GitHub at https://github.com/trouillon-lab/PoolPy.

For manuscripts utilizing custom algorithms or software that are central to the research but not yet described in published literature, software must be made available to editors and reviewers. We strongly encourage code deposition in a community repository (e.g. GitHub). See the Nature Portfolio guidelines for submitting code & software for further information.

Data

Policy information about availability of data

All manuscripts must include a data availability statement. This statement should provide the following information, where applicable:

- Accession codes, unique identifiers, or web links for publicly available datasets
- A description of any restrictions on data availability
- For clinical datasets or third party data, please ensure that the statement adheres to our policy

The dataset containing precomputed designs and performance comparisons is available at https://doi.org/10.5281/zenodo.18660061. The raw DAP-seq sequencing

data is available in the NCBI database under the bioproject number PRJNA1425967.

Research involving human participants, their data, or biological material

Policy information about studies with human participants or human data. See also policy information about sex, gender (identity/presentation), and sexual orientation and race, ethnicity and racism.

Reporting on sex and gender	NA
Reporting on race, ethnicity, or other socially relevant groupings	NA
Population characteristics	NA
Recruitment	NA
Ethics oversight	NA

Note that full information on the approval of the study protocol must also be provided in the manuscript.

Field-specific reporting

Please select the one below that is the best fit for your research. If you are not sure, read the appropriate sections before making your selection.

[x] Life sciences [ ] Behavioural & social sciences [ ] Ecological, evolutionary & environmental sciences

For a reference copy of the document with all sections, see nature.com/documents/nr-reporting-summary-flat.pdf

Life sciences study design

All studies must disclose on these points even when the disclosure is negative.

Sample size	Sample sizes were chosen based on standards in the field. No statistical method was used to predetermine sample size.
Data exclusions	No data was excluded.
Replication	DAP-seq, TSA and RT-qPCR in vitro screens were performed in single technical replicate to reflect a typical use case in large-scale screenings.
Randomization	To generate independent sample draws for ligand-protein interaction assays, 96 samples were randomly selected from a set of 1000, as described in the method section. For spiking of samples in additional thermal shift assays and RT-qPCR experiments, 1 - 5 samples per 96-well plates were randomly spiked with selected standards. All randomized selections were done using Python.
Blinding	The investigators were not blinded to allocation during experiments and outcome assessment.

Reporting for specific materials, systems and methods

We require information from authors about some types of materials, experimental systems and methods used in many studies. Here, indicate whether each material, system or method listed is relevant to your study. If you are not sure if a list item applies to your research, read the appropriate section before selecting a response.

Materials & experimental systems

n/a	Involved in the study
[x]	[ ] Antibodies
[x]	[ ] Eukaryotic cell lines
[x]	[ ] Palaeontology and archaeology
[x]	[ ] Animals and other organisms
[x]	[ ] Clinical data
[x]	[ ] Dual use research of concern
[x]	[ ] Plants

Methods

n/a	Involved in the study
[x]	[ ] ChIP-seq
[x]	[ ] Flow cytometry
[x]	[ ] MRI-based neuroimaging

Plants

Seed stocks	NA
Novel plant genotypes	NA
Authentication	NA
