# Supplementary material for: Combinatorial group testing for efficient scaling across biological applications
Source: Nat Commun. 2026 Aug 25;17:9146. doi: 10.1038/s41467-026-77055-5 (PMC13522496; doi:10.1038/s41467-026-77055-5)
Supplement: Supplementary file 4 [file 41467_2026_77055_MOESM5_ESM.pdf]

nature portfolio

Peer Review File

Combinatorial group testing for efficient scaling across biological applications

Corresponding Author: Dr Julian Trouillon

This file contains all reviewer reports in order by version, followed by all author rebuttals in order by version.

Version 0:

Reviewer comments:

Reviewer #1

(Remarks to the Author)
Dear Editor,

First, I would like to thank the Editor for the opportunity to review the manuscript entitled “PoolPy: Automated combinatorial pooling for high-throughput molecular profiling” by L. Talamanca and J. Trouillon.

The authors aim to simplify and expand the use of combinatorial group testing in large-scale biological experiments. They introduce PoolPy, a unified framework and web platform that enables users to design, benchmark, automate, and decode pooling strategies tailored to specific experimental constraints. By integrating multiple algorithms and supporting both binary and quantitative (multi-readout) assays, PoolPy addresses challenges in method selection, design complexity, and implementation. The objective is to make group testing more accessible and efficient, reducing experimental cost and time while enabling scalable, high-throughput molecular profiling across diverse applications.

The manuscript is well written and clearly presents the concepts and methodology. PoolPy represents a valuable and potentially impactful tool for the community, particularly for scaling combinatorial screening approaches. The inclusion of experimental validation alongside the theoretical framework is a strong aspect of the work, as it effectively illustrates the practical applicability of the approach.

My evaluation will primarily focus on aspects of the manuscript that relate to my expertise in computer-aided drug design. I trust that other reviewers will provide detailed assessments of sections that fall outside this specific area.

Major points:
- The description of the ligand–protein HTS protocol would benefit from additional detail to ensure clarity and reproducibility. While the authors describe the experimental setup in general terms (e.g., screening ligand interactions for human carbonic anhydrase II using pooled designs and liquid-handling automation), some key methodological aspects remain underexplained, such as criteria for defining positive interactions. For instance, it is unclear how signal thresholds are determined or how variability between pools is handled. Providing a more explicit protocol or referencing a standardized HTS workflow would help readers better assess the robustness and transferability of the approach.
- The rationale for selecting carbonic anhydrase II (hCAII) as the model system is not given. Although it is a well-characterized enzyme with known ligands (e.g., acetazolamide, used here as a positive control), the manuscript does not explain why this particular system was chosen over others, or how representative it is of broader drug discovery applications. For example, is hCAII selected because of its strong binding signals, low noise, or suitability for pooled assays? A brief justification would help contextualize the results and clarify if the demonstrated performance is expected to generalize to more complex or less well-behaved targets.
- The ligand screening experiment relies on a simulated library in which a single compound (acetazolamide) is spiked into an otherwise inactive background. While this provides a controlled validation, it does not reflect the complexity of real compound libraries, which may contain promiscuous compounds (e.g., PAINS or aggregators) exhibiting non-specific activity. It would be important for the authors to discuss how their approach would perform in such settings, and if additional controls or filtering steps would be required to mitigate false positives arising from non-specific binders.
- While the framework is designed to identify multiple positive samples in principle, the experimental validation is limited to a single active compound. It would be valuable for the authors to demonstrate or discuss the performance of their approach in

more realistic scenarios where multiple active compounds with varying signal strengths may be present, as typically encountered in HTS campaigns.
- There is no direct comparison to standard HTS in terms of time, cost, or experimental throughput. Including such benchmarks or concrete estimates of speed-up would strengthen the practical relevance of the method for screening applications.

Minor concerns:

- Line 20. “including all most used methods” -> “including all the most used methods”
- Line 187. “to find the real optimal condition” -> “to find the true optimal condition”

(Remarks on code availability)

Reviewer #2

(Remarks to the Author)
This manuscript reports PoolPy, a combinatorial group testing framework integrating design, benchmarking, automation and decoding. The topic addresses a critical need in high throughput screening, and the overall work is solid having publishable value. However, the manuscript suffers from major flaws including unclear definition of novelty, insufficient benchmarking, incomplete validation, avoidance of limitations, and non-standard figure formatting. Major revision is needed before acceptance.
1. The claim of being "superior to existing tools" is only qualitatively stated, without quantitative performance comparisons (for example, number of tests, decoding accuracy, and runtime). Differences between methods are only mentioned in supplementary tables, and the main text does not clearly articulate the innovation and advantages of PoolPy. Quantitative comparisons against the currently prevalent mainstream tools are required to clearly delineate the performance disparities.
2. Only a very low prevalence (1%) scenario was validated; decoding accuracy under moderate to high prevalence (5%–10%) was not tested, making it difficult to assess the method’s generalizability. No comparison with traditional individual testing in terms of cost, time, sensitivity, or throughput was provided. For the protein ligand experiment, only 2 designs were tested, leaving the practical utility of the remaining 8 algorithms unverified. Data on decoding accuracy under moderate to high prevalence should be supplied.
3. The basis for choosing α=0.05 in the LASSO-based continuous decoder is not justified, and no parameter sensitivity analysis was performed. The rationale for applying compressed sensing to multi-readout decoding of DAP-seq data is not validated, and it is not explained how noise or batch effects in omics data are addressed.
4. Figure quality is poor; the images are too small and lack sufficient clarity. Adjustments are required.
5. The DAP-seq and protein-ligand screening experiments lack critical details such as reagent concentrations, reaction conditions, and the number of replicates. Implementation details for the 10 algorithms are vague, preventing reproduction of the benchmarking results.
6. Many abbreviations are used without definition at first appearance, including DAP-seq, LASSO, FWER, STD, etc. These need to be corrected.
7. For the more than 100,000 in-silico screening scenarios, no information is provided on biological replicates, technical replicates, or the statistical tests used.

(Remarks on code availability)
I have carefully read the sections of the manuscript regarding data and code availability, and visited the authors' GitHub repository (https://github.com/trouillonlab/PoolPy) and web platform (https://trouillon-lab.github.io/PoolPy/). Due to time and/or environmental constraints, I was unable to locally install and run the code. Based on the documentation and publicly available data, the code shows good potential for reproducibility. However, since I have not actually run it, I cannot confirm that it is completely bug-free or fully reproducible.

Version 1:

Reviewer comments:

Reviewer #1

(Remarks to the Author)
Thank you for answering my different points.

(Remarks on code availability)
None

Reviewer #2

(Remarks to the Author)
I carefully reviewed the revised manuscript and the authors' response letter. All my prior concerns have been fully resolved with supplementary experiments, quantitative data and detailed methodological descriptions. The manuscript presents a complete, practical PoolPy toolkit validated across diverse biological assays, with clear cost-efficiency advantages demonstrated. No further revisions are needed. I recommend acceptance.

(Remarks on code availability)
I inspected the GitHub repository and web interface of PoolPy. Complete README documents with installation guidance, example notebooks and operation tutorials are provided, which enable researchers to deploy and use the tool easily. Due to local environment constraints, I did not complete full local installation and running tests, but the comprehensive code annotations and well-organized supplementary scripts guarantee good reproducibility of all results described in this paper. This open-source tool is a valuable, accessible resource for the biological screening community.

Open Access This Peer Review File is licensed under a Creative Commons Attribution 4.0 International License, which permits use, sharing, adaptation, distribution and reproduction in any medium or format, as long as you give appropriate credit to the original author(s) and the source, provide a link to the Creative Commons license, and indicate if changes were made.
In cases where reviewers are anonymous, credit should be given to 'Anonymous Referee' and the source.
The images or other third party material in this Peer Review File are included in the article's Creative Commons license, unless indicated otherwise in a credit line to the material. If material is not included in the article's Creative Commons license and your intended use is not permitted by statutory regulation or exceeds the permitted use, you will need to obtain permission directly from the copyright holder.
To view a copy of this license, visit https://creativecommons.org/licenses/by/4.0/

We thank the reviewers for the quick turnaround time in handling our manuscript and for the constructive feedback. We are grateful to the reviewers for their careful reading and detailed comments. Following the reviewers' comments, we have made major changes to the manuscript and the code base, including the generation of new computational and experimental results, which we believe have significantly improved the overall quality and impact of the manuscript, further illustrating the large potential impact of PoolPy across fields. As part of this process, we have additionally improved phrasing and clarity throughout the main text and methods whenever possible.

Changes made to the main text are colored blue.

REVIEWER COMMENTS

Reviewer #1 (Remarks to the Author):

The authors aim to simplify and expand the use of combinatorial group testing in large-scale biological experiments. They introduce PoolPy, a unified framework and web platform that enables users to design, benchmark, automate, and decode pooling strategies tailored to specific experimental constraints. By integrating multiple algorithms and supporting both binary and quantitative (multi-readout) assays, PoolPy addresses challenges in method selection, design complexity, and implementation. The objective is to make group testing more accessible and efficient, reducing experimental cost and time while enabling scalable, high-throughput molecular profiling across diverse applications.

The manuscript is well written and clearly presents the concepts and methodology. PoolPy represents a valuable and potentially impactful tool for the community, particularly for scaling combinatorial screening approaches. The inclusion of experimental validation alongside the theoretical framework is a strong aspect of the work, as it effectively illustrates the practical applicability of the approach.

We thank the reviewer for their positive comments on our work. Particularly, we are glad to see that our goal of democratizing combinatorial group testing by making it more accessible and efficient is clear from the manuscript and well received.

My evaluation will primarily focus on aspects of the manuscript that relate to my expertise in computer-aided drug design. I trust that other reviewers will provide detailed assessments of

sections that fall outside this specific area.

Major points:

- The description of the ligand–protein HTS protocol would benefit from additional detail to ensure clarity and reproducibility. While the authors describe the experimental setup in general terms (e.g., screening ligand interactions for human carbonic anhydrase II using pooled designs and liquid-handling automation), some key methodological aspects remain underexplained, such as criteria for defining positive interactions. For instance, it is unclear how signal thresholds are determined or how variability between pools is handled. Providing a more explicit protocol or referencing a standardized HTS workflow would help readers better assess the robustness and transferability of the approach.

We thank the reviewer for identifying missing information in the ligand-protein screening method section. We have now provided additional details on missing information in the corresponding method section. As highlighted by the reviewer, of particular relevance is the criteria to define positive interactions. Each plate measured contained its own set of four negative control samples, and positive samples were identified as having a Tm greater than three standard deviations above the average of the negative control Tm value distribution for each plate. This is now described in the method section along with additional details.

- The rationale for selecting carbonic anhydrase II (hCAII) as the model system is not given. Although it is a well-characterized enzyme with known ligands (e.g., acetazolamide, used here as a positive control), the manuscript does not explain why this particular system was chosen over others, or how representative it is of broader drug discovery applications. For example, is hCAII selected because of its strong binding signals, low noise, or suitability for pooled assays? A brief justification would help contextualize the results and clarify if the demonstrated performance is expected to generalize to more complex or less well-behaved targets.

hCAII was chosen as a model for this assay because it is a well known and characterized example with multiple studies from different groups reporting thermal shift assays (TSA) experiments demonstrating its interaction with acetazolamide. Because our primary goal was to benchmark the use of combinatorial pooling, and not to validate TSA as a target discovery tool (this is already clear from work in the field), we aimed to select a reliable ground truth system to ensure that any failure to detect a positive hit is strictly due to the pooling design rather than due to the assay. Naturally, any inherent limitations of standard single-well TSA remain present in a pooled format.

However, using pooled assays can bring an additional limitation due to the dilution of samples which might affect more or less different cases based on strength of binding signal, as suggested by the reviewer. Indeed, in pooled assays each sample is often diluted, leading to a dilution of the signal from positive samples, which in some cases can be enough to bring the signal below the detection threshold. We illustrated this limitation with the thermal shift assay data using different concentrations of acetazolamide (which conceptually could also represent a lower-affinity binder), which showed that one of the two used pooling designs failed in detecting the positive sample when using 0.5 mM acetazolamide stock solution, while it succeeded with 5mM.

To further expand on this notion, we generated new TSA data on a range of different pool sizes for pools containing one positive sample (Figure R1). Along with the previous experiments, these new results give a quantitative view of the signal dilution progression in the case of hCAII-acetazolamide and provide a detailed view of which pool sizes (and thus which designs) would be suitable for this interaction when starting from a 5mM drug stock concentration. These results confirm that signal dilution increases with pool size as expected, which thus must be chosen according to the assay's specific limit of detection. If users are studying poorly behaving proteins or weak binders, they can use PoolPy to select designs that minimize signal dilution to preserve signal integrity while still achieving considerable throughput gains.

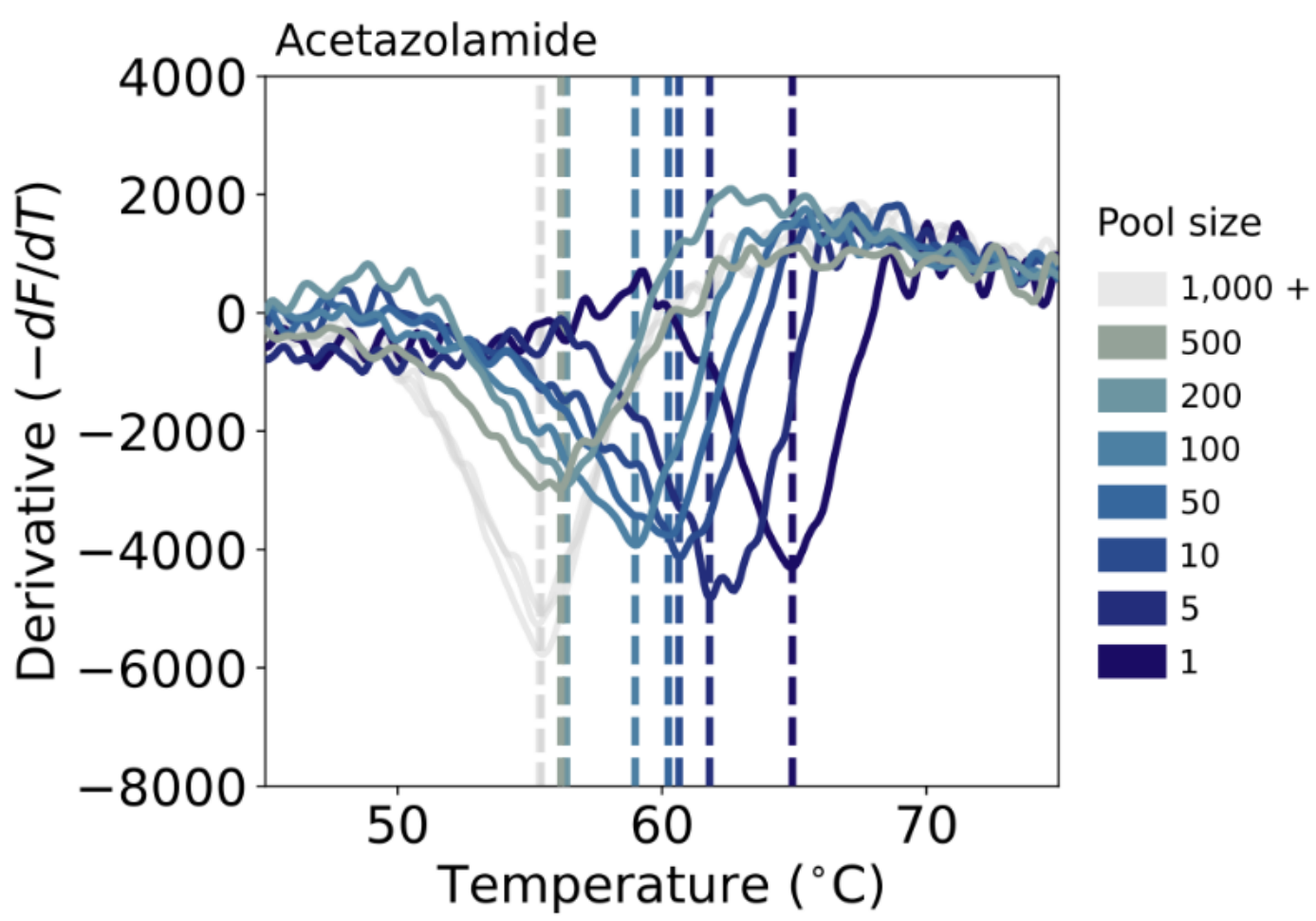

Figure R1: Pool size defines signal dilution in pooled testing. Ligand interaction screening for human carbonic anhydrase II using pools of samples that include one positive sample containing 5 mM acetazolamide, across varying numbers of sample per pool.

We are additionally discussing this aspect in the corresponding result section, with these results added to Figure S8, and in the newly added discussion section. We have also added a sentence on the choice justification for the hCAII case as suggested.

- The ligand screening experiment relies on a simulated library in which a single compound (acetazolamide) is spiked into an otherwise inactive background. While this provides a controlled validation, it does not reflect the complexity of real compound libraries, which may contain promiscuous compounds (e.g., PAINS or aggregators) exhibiting non-specific activity. It would be important for the authors to discuss how their approach would perform in such settings, and if additional controls or filtering steps would be required to mitigate false positives arising from non-specific binders.

We thank the reviewer for raising this important point. Briefly, false positives are not more problematic in pooled testing compared to single testing as long as appropriate pooling design parameters are chosen so that the number of positive samples (importantly this counts both true and false positive) does not exceed the maximum of that design. To account for that, users can adjust the maximum number of positives by also considering the expected number of false positives. However, increasing that threshold leads to pooling designs that are less efficient than

those with a lower maximum number of positives. Practically, if a sample contains a non-specific binder that yields a positive signal, this signal will be split between the different pools containing that sample, leading to the identification of that specific sample as positive, in the same way that it would be with single testing. Thus, the same precautions and filtering steps done for single testing to mitigate false positives apply to pooled testing. Strikingly, group testing can perform better than individual testing in terms of final number of false positives, as (i) less tests are needed (thus generally less "random" false positives occur for a given experiment) and (ii) for a sample to be considered positive all pools in which this sample is present have to be positive. We have now discussed this point in the newly added Discussion section.

- While the framework is designed to identify multiple positive samples in principle, the experimental validation is limited to a single active compound. It would be valuable for the authors to demonstrate or discuss the performance of their approach in more realistic scenarios where multiple active compounds with varying signal strengths may be present, as typically encountered in HTS campaigns.

We agree and have performed the following new experiments, which we believe provide comprehensive and compelling demonstrations of the ability of PoolPy's designs to work with multiple positive samples:

1. hCAII thermal shift assay screen with 2 positive samples containing the two drugs acetazolamide and brinzolamide at different concentrations (added to Figure 3)
2. SARS-COV-2 RT-qPCR testing of sets of samples containing 1, 2 or 5 positive samples with varying viral RNA loads (added to Figure 4)

1. hCAII thermal shift assay

To test scenarios with multiple active compounds that have varying signal strengths, we first performed additional TSA experiments with two drugs known to interact with hCAII: acetazolamide and brinzolamide. To specifically address the reviewer's comment about varying signal strengths, we first tested each drug individually at two different concentrations, which showed a large difference in signal intensity (Figure R2).

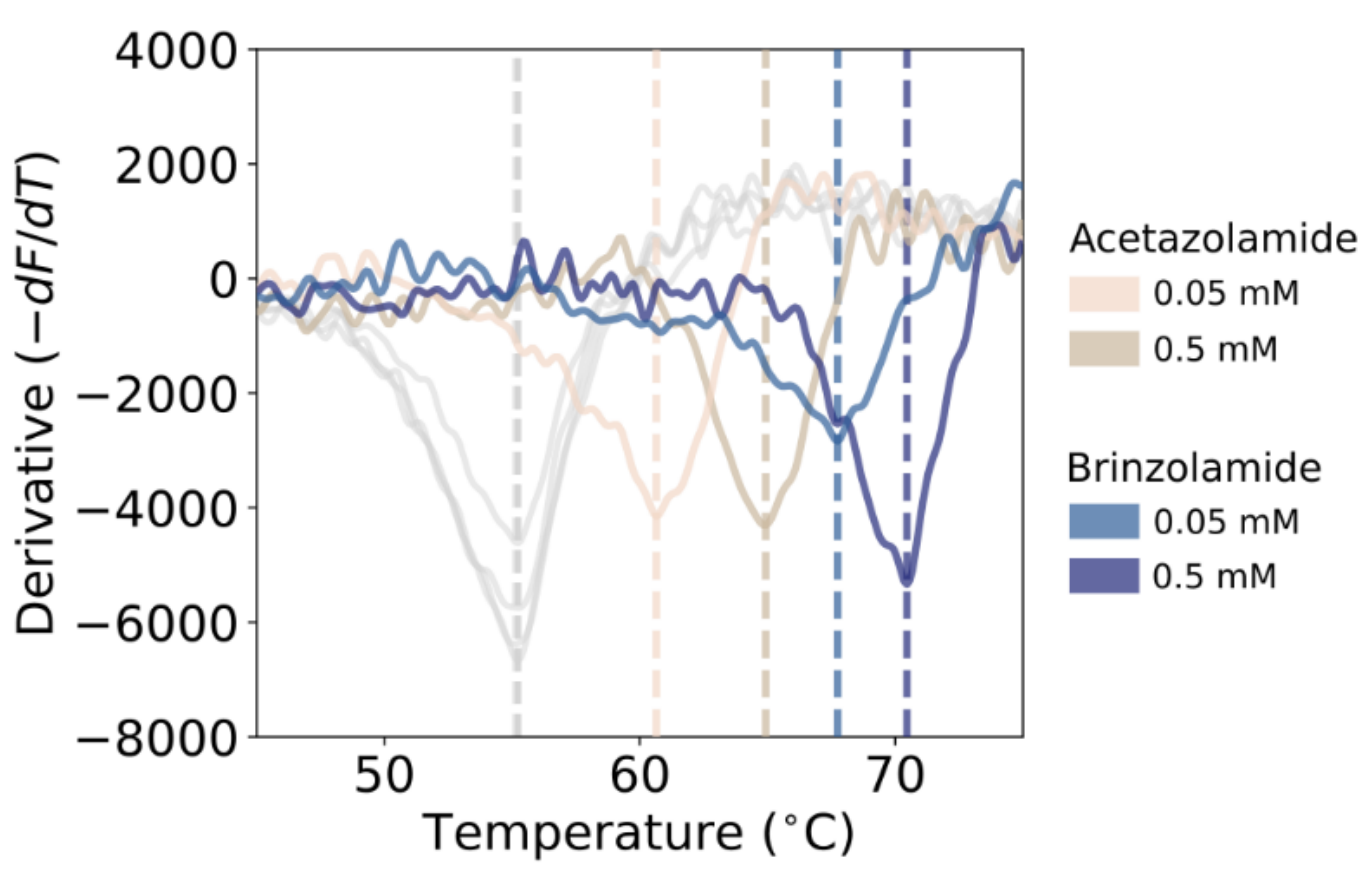

Figure R2: Acetazolamide and brinzolamide show different signal intensities in hCAII interaction assay. Ligand interaction screening for human carbonic anhydrase II with acetazolamide or brinzolamide at two different concentrations. Negative controls are shown in grey.

To illustrate a case with a large difference in signal intensities between positives, we took the two extreme cases: 0.05mM (0.5mM stock) acetazolamide and 0.5mM (5mM stock) brinzolamide, which showed a large difference in Tm of ~ 10°C (Figure R2). We then created a test scenario with 96 samples, including two samples spiked in with either 0.5mM acetazolamide or 5mM brinzolamide. We used the PoolPy web app to generate designs corresponding to this testing scenario and used PoolPy performance comparison to choose the design that minimizes the number of tests needed to identify positives in a single step. This design (the shifted transversal design) works in this scenario by pooling samples in 25 pools that each contains 19 – 20 samples. We pooled the samples accordingly and measured the 25 pools using hCAII TSA (Figure R3). After decoding the results using the PoolPy web app, the two positive samples were correctly identified. This case represents a reduction of about 4-fold (25 tests needed for 96 samples) in resource and reagent use, even with two positive samples that have very different signal intensities. Here, pools containing samples with brinzolamide showed higher Tm than those containing only acetazolamide, as expected, but pool size was still adequate to correctly identify all of them.

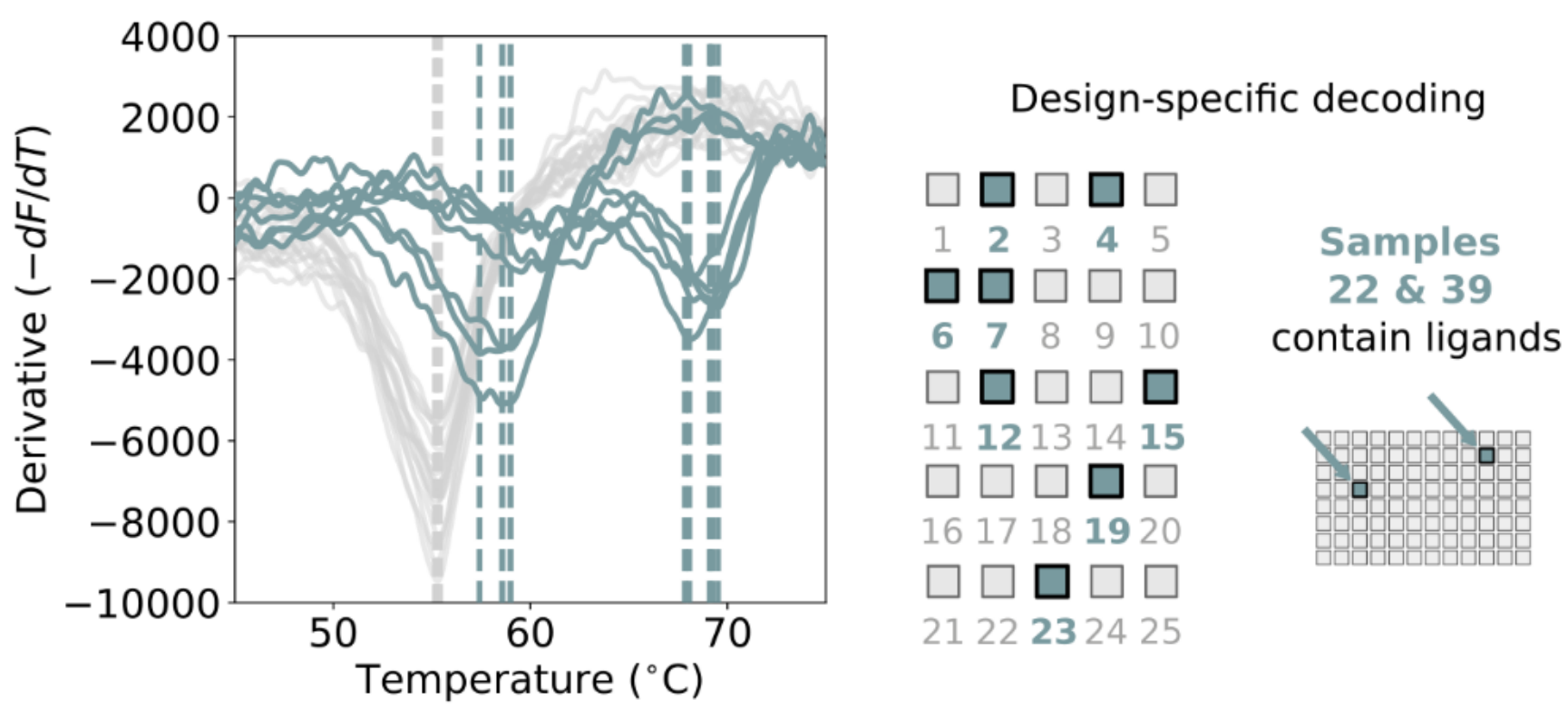

Figure R3: Pooled screen for hCAII ligands with two positive samples. Ligand interaction screening for human carbonic anhydrase II of 25 pools. Positive and negative pools are shown in cyan and grey, respectively.

This example illustrates the power of PoolPy in designing pooled tests across various testing conditions, including with multiple active compounds that have varying signal intensities. These results are now part of Figure 3, and we thank the reviewer for this suggestion which significantly strengthens the manuscript.

2. SARS-CoV-2 RT-qPCR testing

To further explore the use of PoolPy across applications, we also decided to illustrate its use on a different assay type. Because pooled testing has been used and has a large potential for human infection testing, we chose to apply it to viral RNA RT-qPCR assays. Overall, we performed four different pooled experiments, using four different designs, including 1- and 2-steps designs, and spanning sets of 96 samples that include 1, 2 or 5 positive samples which contained various amounts of viral RNA. This set of new experiments provides an additional comprehensive illustration of the use of PoolPy and the performance of its designs across various testing conditions, as detailed below.

To reflect human viral test sampling, we used samples containing human genomic DNA that were spiked or not with SARS-CoV-2 RNA standards and tested them using a commercial kit specific for SARS-CoV-2 RT-qPCR testing (NEB Luna SARS-CoV-2 RT-qPCR Multiplex Assay Kit). Multiplexed RT-qPCR tests were done following manufacturer's instructions, including provided negative and positive controls for each plate, and positive samples were defined as instructed (any amplification, i.e. a Ct value below 40, of the N1 and the N2 SARS-CoV-2 regions denotes a positive sample).

First, we tested a simple scenario with 96 samples including 1 sample that was spiked with SARS-CoV-2 RNA. As in the TSA experiment, we selected the PoolPy design that minimizes test number in these conditions, which in this scenario relies on 7 pools of up to 48 samples. By measuring the 7 pools using RT-qPCR, identifying two positive pools and decoding the results using PoolPy, we could correctly identify the positive sample among 96 (Figure R4). As in the TSA experiment with one positive, this design (the binary design) provides very high reduction in number of tests needed, with in this case only 7 measurements needed to identify one sample among 96, or a 13.7-fold reduction in cost/resource usage.

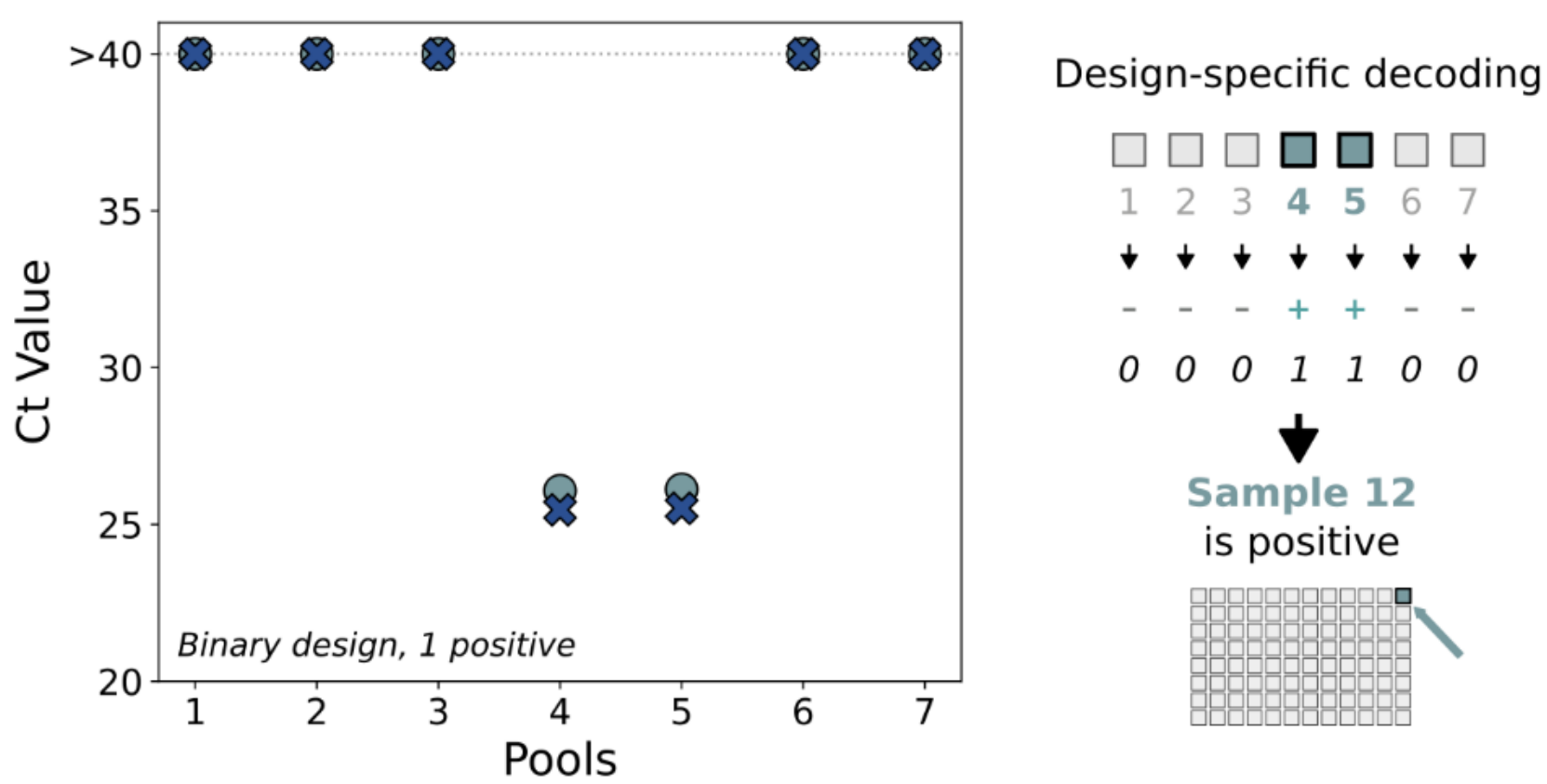

Figure R4: Pooled SARS-CoV-2 RT-qPCR testing of 96 samples with one positive. Markers denote amplification of two different regions of SARS-CoV-2 nucleocapsid RNA (N1: circle, N2: cross). Positive tests show Ct values below 40 for both regions.

Then, we tested a scenario where 2 out of 96 samples were positives. Using PoolPy to compare design performances in this scenario, we chose the design that minimizes test number in this condition (the 3-dim design), which in this case relies on 14 pools initially but requires a second validation step in 89% of cases. Since this can be a problem in cases where time is limiting or when two rounds of measurements are simply not possible, we also selected the design with the lowest number of tests while still guaranteeing to find two positives in a single step (the STD design), which in this case relies on 25 pools. In both cases, we assembled the pools and tested them using SARS-CoV-2 RT-qPCR. The STD design correctly identified the two positive samples (samples 19 and 43) from the 25 measurements (Figure R5a), representing a ~ 4-fold reduction in cost/resource usage. With 14 measurements, the 3-dim design enabled to first narrow down on four putative samples (Figure R5b). In this case, the PoolPy decoder finds that 2 to 4 positive samples are among four identified putative samples (samples 3, 19, 43, and/or 59), which thus need to be re-tested individually for confirmation. After individual testing of these four samples, we could correctly identify samples 19 and 43 as positive (Figure R5b). In this case, while two

rounds of experiments were needed, two positives could be identified out of 96 with 18 tests only, representing a ~ 5-fold reduction in cost/resource usage.

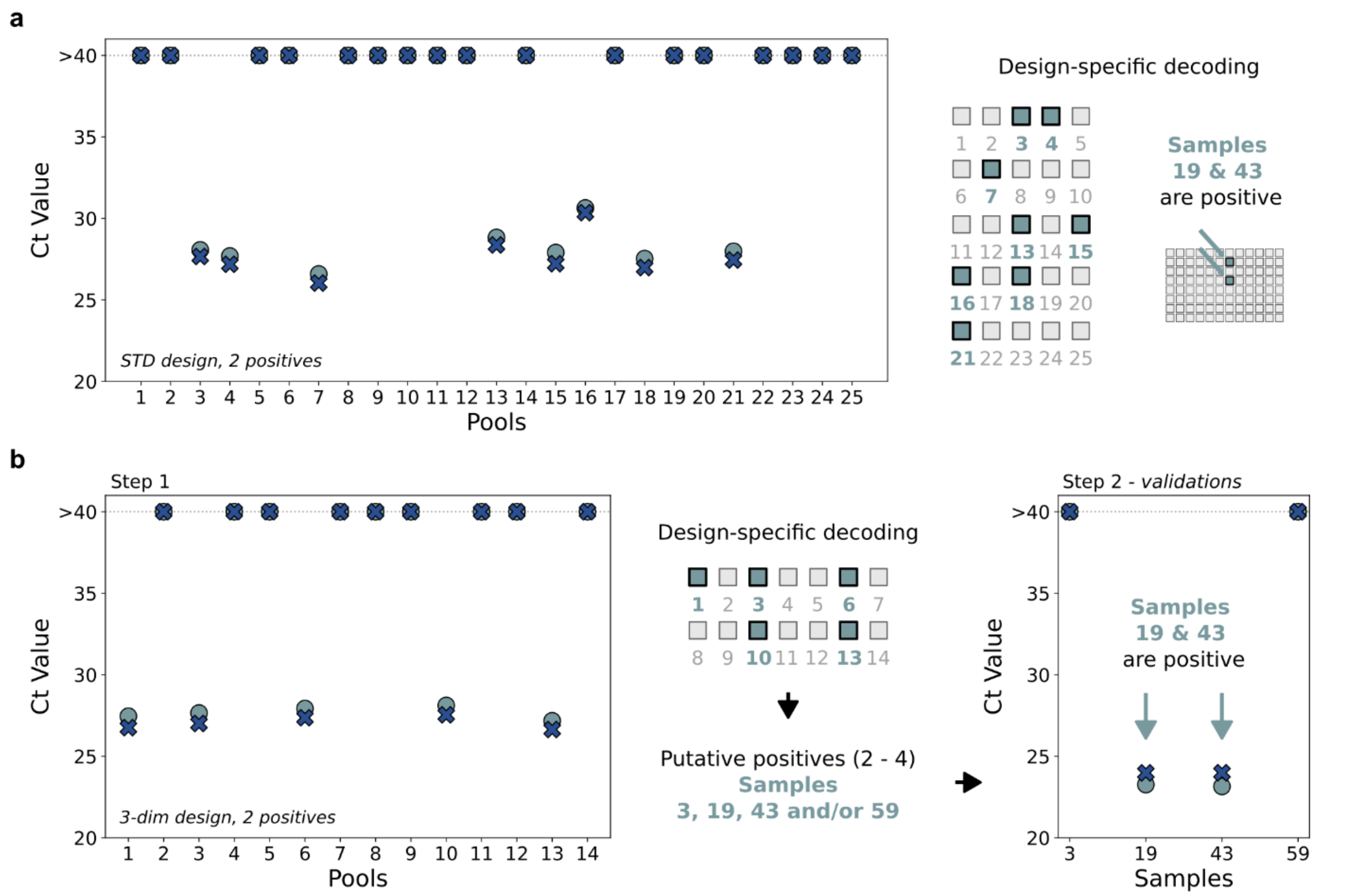

Figure R5: Pooled SARS-CoV-2 RT-qPCR testing of 96 samples with two positives. Results are shown for two pooling designs: 1-step STD design (**a**) and 2-step 3-dim design (**b**). Markers denote amplification of two different regions of SARS-CoV-2 nucleocapsid RNA (N1: circle, N2: cross). Positive tests show Ct values below 40 for both regions.

Finally, we tested a high prevalence scenario with 96 samples containing 5 positives. Here again, we selected the design with the lowest number of tests needed (in that case the matrix design), which relies on a first round of 20 pooled tests and, at this high prevalence, a second round of validation for individual tests. After pooling the samples and testing the 20 pools using SARS-CoV-2 RT-qPCR testing, the PoolPy decoder identified 16 samples as possibly positive (Figure R6a). We then individually tested these 16 samples, leading to the correct identification of all 5 positive samples (Figure R6b). While the matrix design typically performs worse than most other PoolPy designs at low prevalence, it scales well with increasing prevalence and still allows substantial test number reduction at prevalence of 5 – 10 %, where the benefits of pooled testing decrease compared to low prevalence. In this case, we could identify 5 positives within 96 samples in 36 tests, representing a 2.66-fold reduction in cost/resource usage.

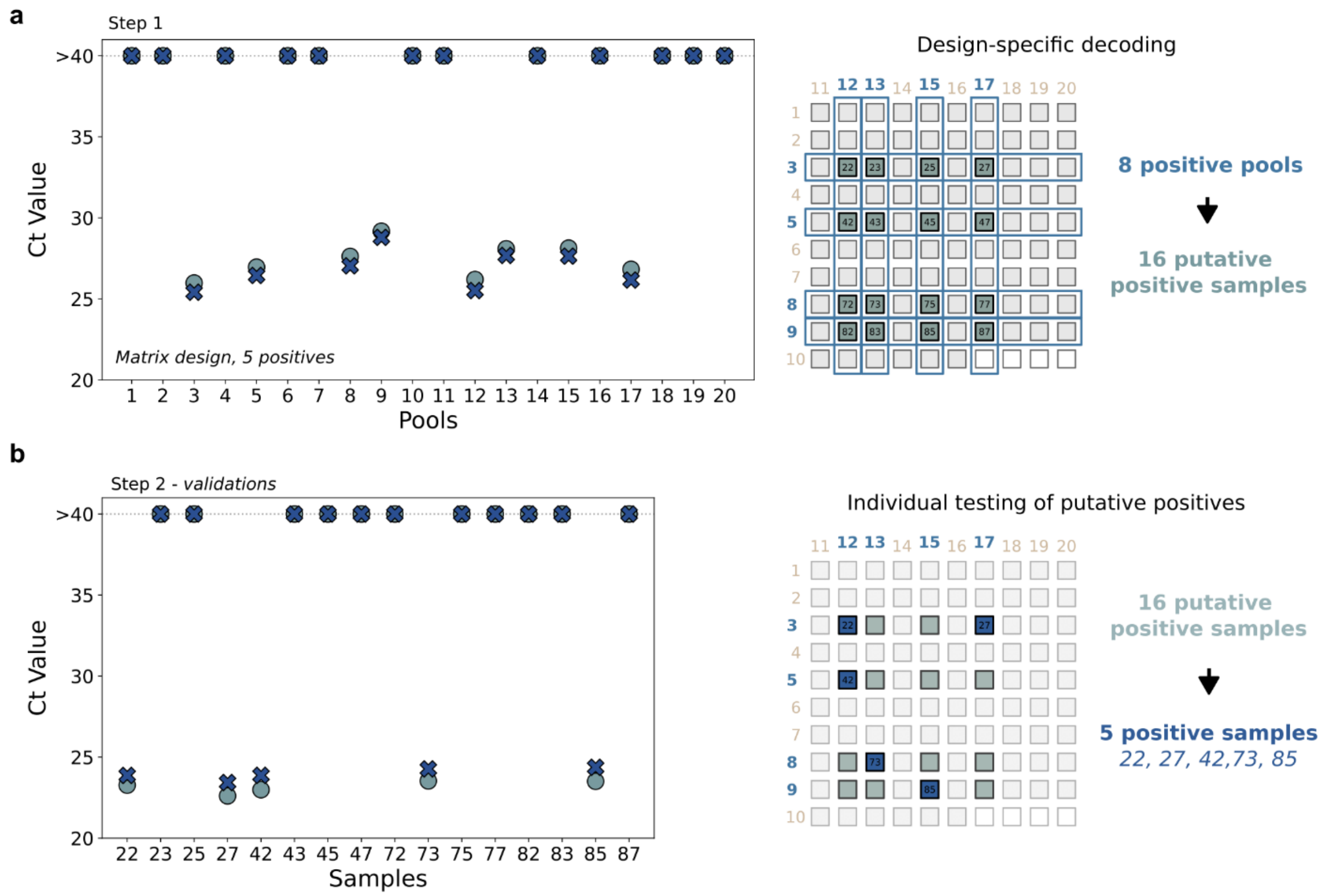

Figure R6: Pooled SARS-CoV-2 RT-qPCR testing of 96 samples with five positives. Results are shown for the step 1 (**a**) and step 2 (**b**) of pooled testing following the matrix design. Markers denote amplification of two different regions of SARS-CoV-2 nucleocapsid RNA (N1: circle, N2: cross). Positive tests show Ct values below 40 for both regions.

- There is no direct comparison to standard HTS in terms of time, cost, or experimental throughput. Including such benchmarks or concrete estimates of speed-up would strengthen the practical relevance of the method for screening applications.

We agree and we have added / made clearer such comparison in multiple places in the manuscript. Particularly, we have now quantified cost reductions for pooled assays for low prevalence cases (Figure R7a) and across prevalence values (Figure R7b). These new plots were added as parts of Figure 1 and Figure 2, respectively, and show that PoolPy designs typically enable major cost reduction by reducing the number of tests needed by ~ 40 to >98%. This is also consistent with our experimental validations, which in these specific cases ranged from 60 to 93% cost reduction by the PoolPy designs. We have also highlighted that fact in the corresponding sections. Overall, we have now put more emphasis on increased throughput and cost reduction throughout the manuscript, as well as in the abstract.

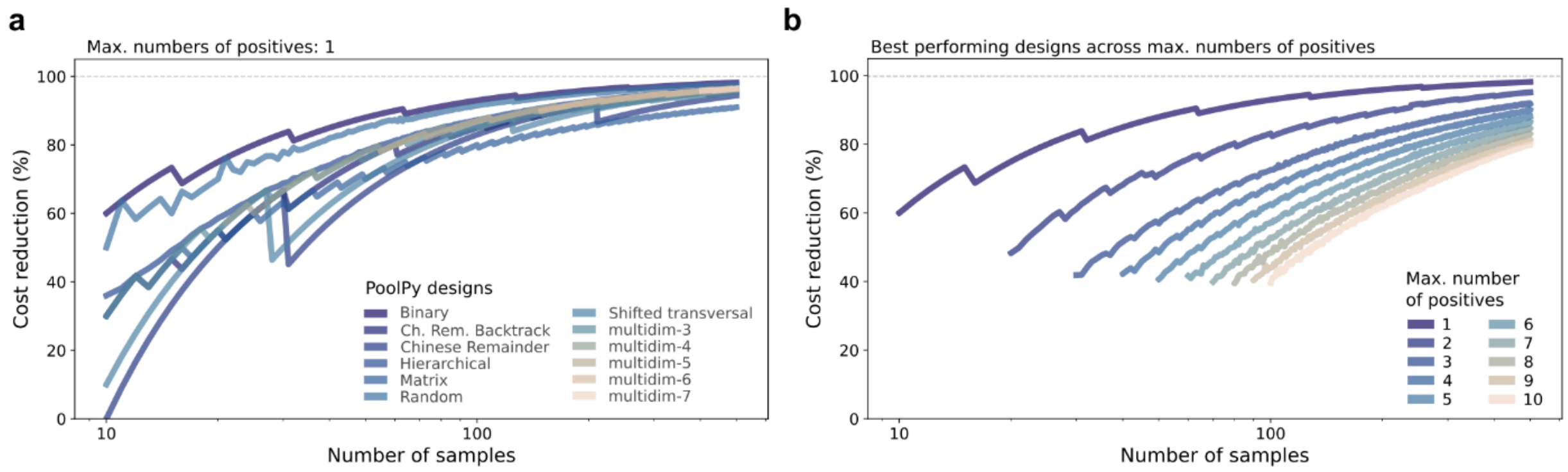

Figure R7: Cost reduction enabled by PoolPy designs. (**a**) Cost reduction, derived from the reduction in test numbers, enabled by the different PoolPy designs over 10 to 500 samples for cases with at most one positive sample. (**b**) Cost reduction, derived from the reduction in test numbers, enabled by the best performing PoolPy design at each number of samples (10 – 500) and max. number of positives (1 – 10) for any prevalence up to 10%.

Minor concerns:

- Line 20. “including all most used methods” -> “including all the most used methods”

Done.

- Line 187. “to find the real optimal condition” -> “to find the true optimal condition”

Done.

Reviewer #2 (Remarks to the Author):

This manuscript reports PoolPy, a combinatorial group testing framework integrating design, benchmarking, automation and decoding. The topic addresses a critical need in high throughput screening, and the overall work is solid having publishable value. However, the manuscript suffers from major flaws including unclear definition of novelty, insufficient benchmarking, incomplete validation, avoidance of limitations, and non-standard figure formatting. Major revision is needed before acceptance.

1. The claim of being "superior to existing tools" is only qualitatively stated, without quantitative performance comparisons (for example, number of tests, decoding accuracy, and runtime). Differences between methods are only mentioned in supplementary tables, and the main text does not clearly articulate the innovation and advantages of PoolPy. Quantitative comparisons against the currently prevalent mainstream tools are required to clearly delineate the performance disparities.

We thank the reviewer for this comment, as it is an important point. We have now dedicated the first Results section to presenting PoolPy and its superior/novel aspects, including the

performance comparison described below. In addition to being more performant in terms of cost reduction and run time (see below), PoolPy integrates far more designs and is the first tool to provide a comprehensive benchmarking of performance, enabling rational user choice based on real-world constraints. It is also the first tool to provide both a single- and a multi-readout decoder (enabling e.g. our DAP-seq example), as well as it is the first tool to support a binary and continuous readout (enabling compressed sensing approaches). Additional key functionalities include the generation of liquid-handling robot method files, a prevalence-based parameter estimation, and a user-friendly web interface with on-the-fly performance estimation and design generation over very large range of conditions. By integrating these numerous new capabilities, we believe PoolPy represents a major advancement over the fragmented tools currently available to experimentalists.

We want to highlight that the main limitation in doing a performance comparison is the near-complete lack of existing tools to perform combinatorial group testing experiments. For example, to the best of our knowledge there is currently no tool to design and decode pooled assays for a given number of samples, making a proper comprehensive quantitative comparison virtually impossible.

Most combinatorial designs are reported in technical papers that focus on the mathematical implementation and theory of a single design type and typically do not provide code or tools (e.g. Thierry-Mieg et al., 2006 - BMC Bioinfo., or Eppstein et al., 2006 - SIAM J. Comput.). Even when the focus is not on the math, studies still rarely provide ready-to-use code or tools (e.g. Mutesa et al., 2021 - Nature). Some combinatorial pooling tools exist that are highly tailored to specific applications (e.g. the NIST tool for bug testing in software engineering) but they are typically unusable outside of the intended use. Other tools exist for specific sub-tasks of pooled testing and are noteworthy, e.g. PoolTools (McLure et al., 2021 - Env. Mod. & Soft.) and the Covid Pool Tool (Datto 2020 - Duke U.), although their very limited functionalities do not allow for quantitative performance comparisons.

One existing tool with functionalities closest to PoolPy is binGroup2 (Bilder et al., 2024 - R J.), which still has major differences compared to PoolPy, such as the fact that binGroup2 works with prevalence only and ignores the actual number of samples to test. As asked by the reviewer, we have now performed a performance comparison of PoolPy with binGroup2, accommodating as best as possible these differences in usability, the results of which have been added to Figure S2 and commented in the main text.

To summarize the detailed explanations below, PoolPy covers the two types of designs implemented in binGroup2 as well as 8 additional ones, PoolPy consistently performs better than binGroup2 across any performance indicators (e.g. number of tests or number of steps) and any prevalence values, PoolPy runs significantly faster than binGroup2 and offers a user-friendly web interface (in addition to the code) while binGroup2 is strictly code-based.

A first key difference between binGroup2 and PoolPy is in the number of designs supported. binGroup2 supports two design types (hierarchical and matrix; both part of PoolPy) while PoolPy supports 10, including all the most used designs and a design introduced here. This is a major advantage because the performance of each design varies greatly between testing conditions.

Indeed, our *in silico* benchmarking showed that even within a relatively small range of number of samples, six different designs performed best in at least one condition (Figure 2d), highlighting the benefit of being able to compare design performances for each use case. The sentence describing the higher number of supported design was now moved to the first section of the results, along with the new description of the performance comparison.

It is key to note here that since binGroup2 is strictly prevalence-centric and optimizes pooling configurations under an infinite population assumption, it ignores the actual numbers of samples to be tested. On the other hand, PoolPy is designed for constrained optimization tailored to the exact number of samples to be tested and a given maximum number of positives as a threshold. Aiming for the most equitable evaluation under these mismatched paradigms, we standardized the benchmarking by matching prevalence and the two input parameters of PoolPy so that the variances in the number of drawn positive samples differ by less than 10% between the two approaches across the range of tested prevalences (see Methods for details).

In this comparison, PoolPy provided better designs than binGroup2 for both number of tests and number of steps needed (Figure R8a) for any prevalence up to 10%, and does so faster than binGroup2 (Figure R8b). At low prevalence, PoolPy provides highly efficient designs with e.g. 0.01 test per sample needed at 0.1% prevalence, or a 100-fold reduction in cost, solved in a single step, while binGroup2 provides a 31-fold reduction and requires two steps (i.e. two rounds of testing) in this case. At higher prevalence, while the difference in test per sample decreases between the two tools, PoolPy designs consistently require less rounds of testing than binGroup2.

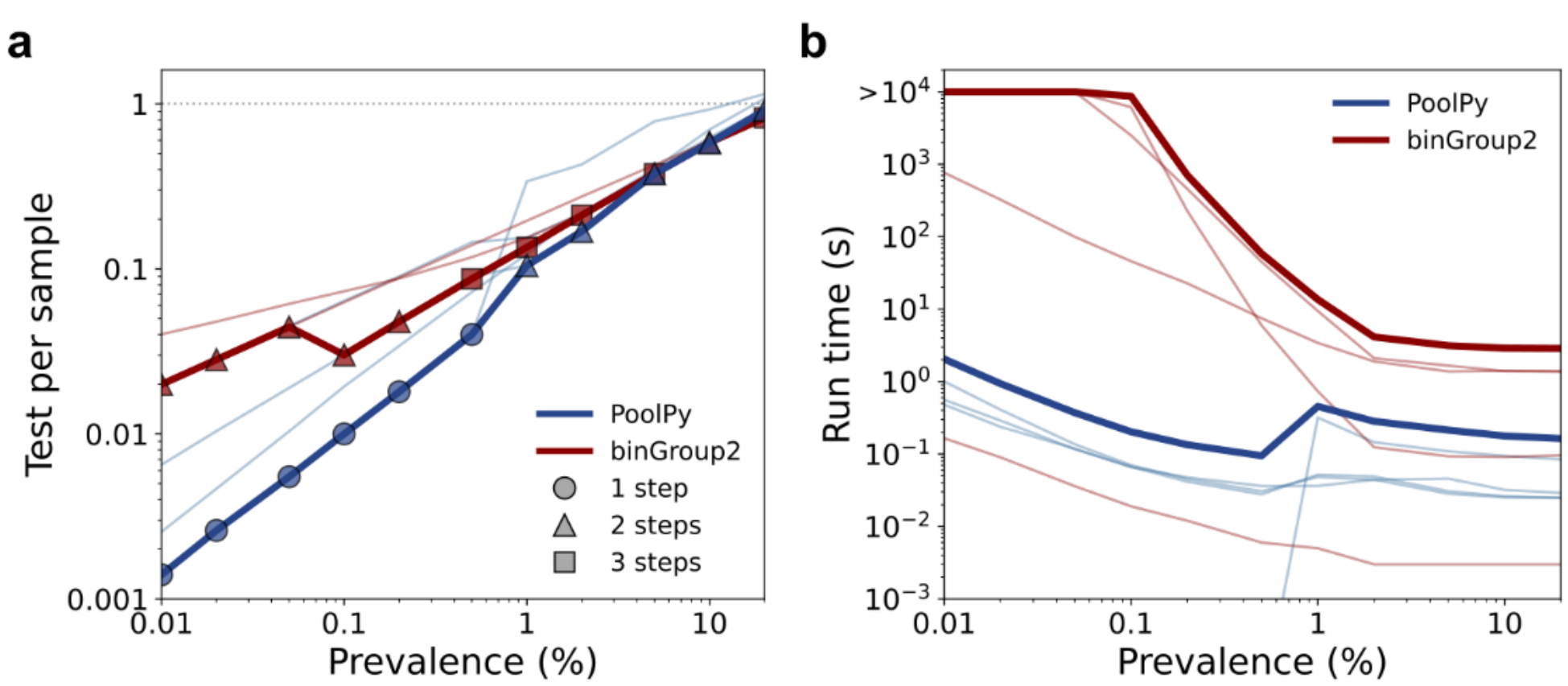

Figure R8: Performance comparison of PoolPy with binGroup2. (**a**) Test per sample needed to identify positive samples at different prevalences (0.01–20%). Individual designs are shown as faded lines and the best design of each tool is shown as a bold line. The number of steps needed by the corresponding design is indicated by the marker shape. (**b**) Run time needed to generate pooling designs. The bold lines denote the total run time needed to generate all shown designs.

Although prevalence values higher than 10% are typically not well suited for group testing due to the high number of positives, this comparison led us to investigate the binGroup2 hierarchical design which required slightly less tests than our hierarchical design when prevalence is above 10% (top right corner of Figure R8a where the binGroup2 line goes below the PoolPy line). This design is based on a pre-defined number of steps and binGroup2 iteratively tries different pool sizes to minimize the test/sample ratio at a given prevalence. While binGroup2 does so iteratively, explaining its long compute time (Figure R8b), we realized that this problem can be solved analytically. We thus have implemented a new analytical version of the binGroup2 designs, which are now also part of PoolPy. This analytical implementation dramatically decreases the time

needed to generate the binGroup2 design, <0.3 second for any prevalence vs. seconds to hours with binGroup2 (Figure R9), allowing its easy implementation across any prevalence values directly on the fly on the web platform of PoolPy.

Secondly, while binGroup2 uses a pre-determined number of steps, we realized that the optimal number of steps varies. We thus improved our version of the binGroup2 design by generating an optimal hierarchical design that finds both the optimal group size and number of steps. Since high number of steps can be limiting in the lab, we also added the option to define a user-based maximum number of steps for higher flexibility. Additionally, we have also improved their matrix design by adding a feature that finds the optimal number of dimensions for such design type (matrix designs are 2-dimension pooling designs), enabling designs with > 2 dimensions as well. Overall, we have now implemented an optimal version of the two prevalence-based designs of binGroup2, which run several orders of magnitude faster than binGroup2 and have additional functions. These prevalence-based designs are now implemented as part of the Prevalence section of the PoolPy app and described in Supplementary Note 2.

Overall, our search and comparison of comparable tools show that PoolPy performs significantly better than other tools in terms of (i) number and type of supported designs, (ii) design performance to reduce cost, (iii) run time and (iv) usability. This is now highlighted in the main text and Figure S2.

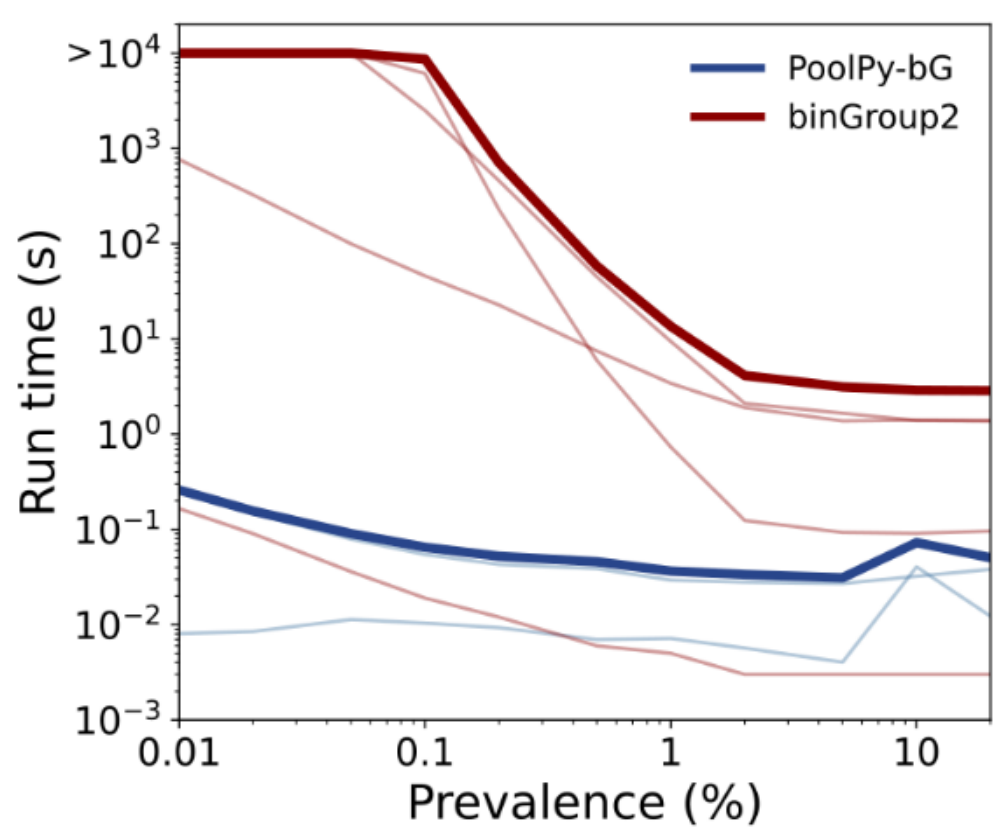

Figure R9: Run time comparison of PoolPy's implementation of binGroup2 designs with binGroup2. Run time needed to generate the binGroup2 pooling designs across a range of prevalence values (0.01-20%). The bold lines denote the total run time needed to generate all shown designs.

2. Only a very low prevalence (1%) scenario was validated; decoding accuracy under moderate to high prevalence (5%–10%) was not tested, making it difficult to assess the method's generalizability. No comparison with traditional individual testing in terms of cost, time, sensitivity, or throughput was provided. For the protein ligand experiment, only 2 designs were tested, leaving the practical utility of the remaining 8 algorithms unverified. Data on decoding accuracy under moderate to high prevalence should be supplied.

As described in our answer to reviewer 1 above, we have now performed several new experiments to comprehensively show the performance of PoolPy designs at higher prevalence values. These include new hCAII thermal shift assays with two different drugs and new SARS-COV-2 RT-qPCR testing with prevalences of ~1, 2 and 5%. In all cases, the pooled designs

allowed to correctly identify all positive samples, even when they exhibited varying signal intensities, in much fewer tests (see above for details and Figures R1 – R6).

Concerning the comparison to individual testing, we have now added quantitative comparisons in the main text and figures 1 and 2 (Figure R7), as well as throughout the manuscript for experimental data, as described in the corresponding response to reviewer 1 above.

In each use case, we indeed use one or two of the proposed PoolPy designs. That is intended to reflect the typical use of PoolPy. As any user would, we select the designs that are most suited to a specific use case (specific number of samples and prevalence) to reduce test number (or signal dilution). That was one of the key reasons to integrate an easy way to compare design performance within PoolPy: different designs will be optimal to use for different use cases. In our experimental examples, we thus use PoolPy to compare the theoretical performances of each design for a specific use case and then select the most suited design to use based on that comparison (such comparison can easily be obtained on the main page of the web app). As part of our new experimental results, we have now illustrated the use of four different designs across different use cases. As shown in Figure 2, the most suited design to use depends on the specific constraints one is trying to address (e.g. reducing test number, or reducing signal dilution etc..) and vary widely between screening conditions (total number and samples and maximum number of positives).

3. The basis for choosing α=0.05 in the LASSO-based continuous decoder is not justified, and no parameter sensitivity analysis was performed. The rationale for applying compressed sensing to multi-readout decoding of DAP-seq data is not validated, and it is not explained how noise or batch effects in omics data are addressed.

We agree with the reviewer and have modified the continuous decoder accordingly. Therefore, we have now updated our continuous decoder to allow user-adjustable parameters, including alpha.

In fact, our continuous decoder does not only implement the LASSO-based approach but an elastic net approach (which contains LASSO and ridge regression), integrating the two parameters alpha and l1_ratio. As suggested by the reviewer, we have now added the option to perform a parameter grid search over ranges of these two parameters to perform a parameter sensitivity analysis. This new PoolPy grid search function decodes data across combinations of alpha and l1_ratio, and implements four different error functions for optimization (mean square error, mean absolute error, root mean square error and r-squared) that can be chosen by the user. This is now described in the corresponding method section and on the PoolPy github.

To help users navigating these functions, we have now added an example use of this grid search as part of the ready-to-use 'decode_interface.ipynb' decoder notebook on the PoolPy github. This notebook, along with the 'pool_interface.ipynb' notebook for pooling design, provides an intermediate user experience between the fully command-based and the user-friendly web app uses of PoolPy. Using these notebooks, which also contain example pooling sets and synthetic data, users with basic Python knowledge can use all PoolPy functions locally while getting visual

representations of their data and results. There, we added an example use case of the elastic net decoder and the grid search function, where we assessed the performance of the continuous decoder in reconstructing data across a range of l1_ratio and alpha, generating a correlation heatmap to identify the best suited parameters (Figure R10). Users can easily re-run this example or substitute their own data to perform such analysis.

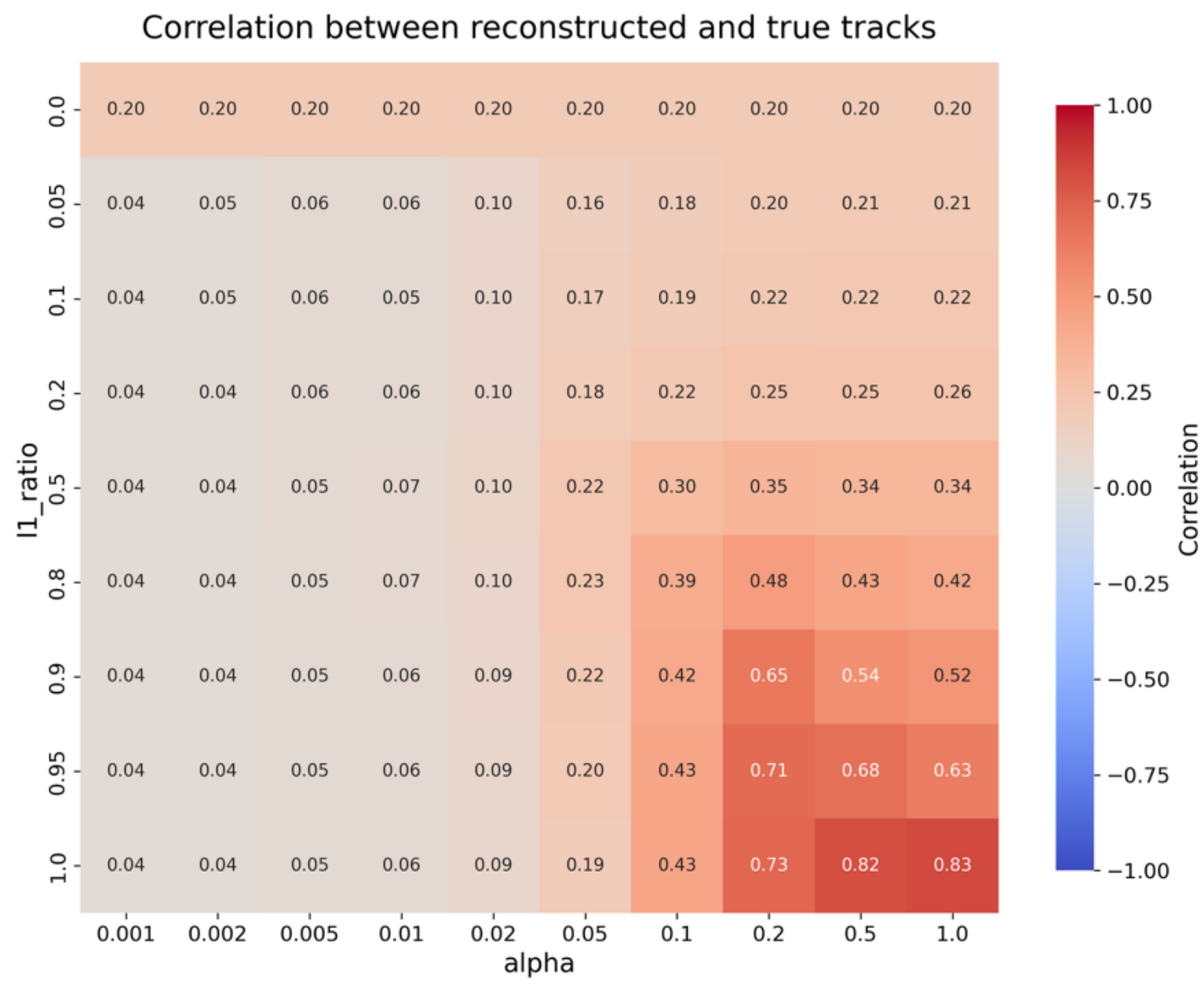

Figure R10 : PoolPy parameter grid search for continuous decoding. Heatmap illustrating the correlation between true data and signals reconstructed by PoolPy across ranges of l1_ratio and alpha. Synthetic data containing 1,000 samples with 100 readouts each was used and pooled using the 3-dim design before decoding evaluation by the PoolPy continuous decoder.

Concerning DAP-seq decoding, it is important to note the distinction between (i) the single/multi-readout and (ii) the binary/continuous aspects of our decoder. Indeed, our decoder is flexible and, in addition to binary single readout, allows for multi-readout decoding of either binary or continuous signals. Since the standard output of DAP-seq is binary, i.e. statistically enriched peak regions presence/absence, our DAP-seq data was decoded in binary multi-readout mode. We have modified the corresponding section to make that point clearer. While we added the continuous decoder as an additional feature for specific applications, most data types (including omics data) can be analyzed in a binary manner, whether single- or multi-readout assays, using assay-specific thresholds, e.g. statistically significant / non-significant.

Concerning noise and batch effect in omics data, PoolPy is designed to work with processed results as readout. Therefore, noise reduction, background subtraction, normalization or other batch-effect corrections are handled entirely by standard upstream bioinformatics pipelines in an application-specific manner (e.g., peak enrichment analysis against background control via MACS3 for DAP-seq) prior to the pooled decoding analysis done by PoolPy. We have now added information on this aspect in Supplementary Note 3.

4. Figure quality is poor; the images are too small and lack sufficient clarity. Adjustments are required.

As part of our major re-working of the manuscript, all main figures were reformatted. Through this process, we have also increased size as clarity wherever possible. Specifically, Figure 1 is now split into two figures to illustrate the cases where (i) up to one positive sample is present or (ii) more than one positive sample is expected. In this process, most plots were enlarged and modified for clarity. Additionally, the thermal shift assay figure, which had the smallest plots and font sizes, was now fully re-worked to both integrate the new TSA data and enlarge and increase clarity of existing plots. A new figure has also been created for the RT-qPCR data with clarity and size in mind as well. The smallest font sizes and plots were also enlarged in the DAP-seq figure.

5. The DAP-seq and protein-ligand screening experiments lack critical details such as reagent concentrations, reaction conditions, and the number of replicates. Implementation details for the 10 algorithms are vague, preventing reproduction of the benchmarking results.

We have now added additional information regarding technical details in all experimental method sections. We have added all information regarding typical technical details (e.g. reagent concentrations, reaction conditions, replicate conditions etc…) anywhere we identified it as missing. If anything is still missing/unclear, we would gladly provide additional information as it is critical to us that the method section is comprehensive.

The implementations of the 10 algorithms are described as part of the 7-page long “Implementation of group testing designs” Methods section, each design detailed in its own sub-section. They are all fully integrated into PoolPy and can be used individually or all together at once directly on the web app, or using a single command as described on the github. Concerning the procedure of benchmarking using the implemented algorithms, key information was indeed missing and we have now added a new method paragraph dedicated to this benchmarking (“*In silico* design benchmarking”), which provides detailed information on the procedure used, including how to reproduce our results and which commands would re-create our benchmarking dataset.

6. Many abbreviations are used without definition at first appearance, including DAP-seq, LASSO, FWER, STD, etc. These need to be corrected.

We have defined abbreviations at first appearance.

7. For the more than 100,000 in-silico screening scenarios, no information is provided on biological replicates, technical replicates, or the statistical tests used.

As described in an answer above, we have now added a new method paragraph explaining the procedure used for the *in silico* benchmarking.

Reviewer #2 (Remarks on code availability):

I have carefully read the sections of the manuscript regarding data and code availability, and

visited the authors' GitHub repository (https://github.com/trouillonlab/PoolPy) and web platform (https://trouillon-lab.github.io/PoolPy/). Due to time and/or environmental constraints, I was unable to locally install and run the code. Based on the documentation and publicly available data, the code shows good potential for reproducibility. However, since I have not actually run it, I cannot confirm that it is completely bug-free or fully reproducible.

We thank the reviewer for their positive comment. On our side, we have also asked colleagues to test installing and using PoolPy, which worked in their hands. As part of addressing other comments above, we now also describe which commands would re-create our benchmarking dataset, further facilitating reproducibility.

This is critical to us, and that is also why we do not provide PoolPy simply as a package but we also developed a user-friendly web app. This app allows users to easily and in a few seconds make design comparisons and perform all needed steps to a successful pooled experiment, even without coding or math expertise.
